# Evolution and phylogeny of the deep-sea isopod families Desmosomatidae Sars, 1897 and Nannoniscidae Hansen, 1916  (Isopoda: Asellota)

**DOI:** 10.1007/s13127-021-00509-9

**Published:** 2021-10-13

**Authors:** Saskia Brix, Christoph Held, Stefanie Kaiser, Robert M. Jennings, Amy Driskell, Angelika Brandt

**Affiliations:** 1 University of Hamburg, German Centre for Marine Biodiversity Research (DZMB), Martin-Luther-King-Platz 3, 20146 Hamburg, Germany; 2 Alfred Wegener Institute Helmholtz Centre for Polar and Marine Research, Am Alten Hafen 26, 27568 Bremerhaven, Germany; 3 Department of Invertebrate Zoology and Hydrobiology, University of Łódź, Banacha St. 12/16, 90-237 Łódź, Poland; 4 Zoological Museum, University of Hamburg, Martin-Luther-King-Platz 3, 20146 Hamburg, Germany; 5 Biology Department, Temple University, 1900 N. 12th Street, Philadelphia, PA 19122 USA; 6 Laboratories of Analytical Biology, Smithsonian Institution, National Museum of Natural History, 10th St. at Constitution Ave, Washington, DC 20530 USA; 7 Senckenberg Research Institute and Natural History Museum, Senckenberganlage 25, 60325 Frankfurt am Main, Germany; 8 Institute for Ecology, Evolution and Diversity, Goethe-University of Frankfurt, FB 15, Max-von-Laue-Str. 13, 60439 Frankfurt am Main, Germany

**Keywords:** Atlantic Ocean, Abyssal, Molecular phylogeny, Taxonomy, Henningian method

## Abstract

In the deep sea, the phylogeny and biogeography of only a few taxa have been well studied. Although more than 200 species in 32 genera have been described for the asellote isopod families Desmosomatidae Sars, 1897 and Nannoniscidae Hansen, 1916 from all ocean basins, their phylogenetic relationships are not completely understood. There is little doubt about the close relationship of these families, but the taxonomic position of a number of genera is so far unknown. Based on a combined morphological phylogeny using the Hennigian method with a dataset of 107 described species and a molecular phylogeny based on three markers (COI, 16S, and 18S) with 75 species (most new to science), we could separate Desmosomatidae and Nannoniscidae as separate families. However, we could not support the concept of the subfamilies Eugerdellatinae Hessler, 1970 and Desmosomatinae Hessler, 1970. Most genera of both families were well supported, but several genera appear as para- or even polyphyletic. Within both families, convergent evolution and analogies caused difficulty in defining apomorphies for phylogenetic reconstructions and this is reflected in the results of the concatenated molecular tree. There is no biogeographic pattern in the distribution as the genera occur over the entire Atlantic and Pacific Ocean, showing no specific phylogeographical pattern. Poor resolution at deep desmosomatid nodes may reflect the long evolutionary history of the family and rapid evolutionary radiations.

**Supplementary Information:**

The online version contains supplementary material available at 10.1007/s13127-021-00509-9.

## Introduction

Despite earlier assumptions of a vast homogeneous environment, the deep sea (i.e., areas below the shelf break of around 200 m) encompasses a high diversity of benthic habitats and related fauna. However, with less than 1% of the deep-sea floor being explored, and most of this sampling concentrated in the Northern Hemisphere, it is probably also one of the least known ecosystems (Gage & Tyler, 1991; Stuart et al., 2008; Ramirez Llodra et al., 2010). In the absence of major biogeographic or physical barriers (compared to shelf environments) as well as several past anoxic events that caused extinction of at least parts of the deep-sea fauna (White, 1988), the deep sea’s high diversity is quite remarkable. Nevertheless, knowledge and understanding of the mechanisms and drivers of population divergence and speciation in the deep sea remain scarce (Rex & Etter, 2010).

Although fossils are apparently lacking prior to the Late Cretaceous, there are several lines of evidence arguing for recurring recolonization of the deep sea from shelf habitats (Kawagata et al., 2005; Thuy et al., 2014; Yasuhara et al., 2009) and it seems likely that most of the contemporary deep-sea fauna evolved from ancestors entering the deep sea after the late Cretaceous/Paleocene anoxic events (99–56 mya). However, based on biogeographic as well as molecular data, there is also evidence that at least some of the deep-sea fauna may have survived past anoxia in situ (e.g., as demonstrated for some isopod and echinoderm lineages; Lins et al., 2012; Thuy et al., 2014; Wilson, 1998, 1999).

To date, the phylogeny and biogeography of few faunal taxa have been studied well enough across bathymetric and geographic gradients. Among these, isopods are probably one of the best-known groups, and therefore represent an ideal model to study phylogenetic patterns and underlying processes in a deep-sea context (Brandt et al., 2007; Hessler & Thistle, 1975; Hessler et al., 1979; Kussakin, 1973; Osborn, 2009; Raupach et al., 2004, 2009; Wilson, 1999). Isopods in the asellote superfamily Janiroidea are an especially dominant and diverse faunal taxon comprising of several families, which have probably long thrived in the deep sea and which exhibit distinct morphological adaptations to deep-sea conditions (such as lack of eyes; e.g., Brandt, 1992; Hessler et al., 1979; Lins et al., 2012; Wilson, 1998, 2017). Phylogenetic patterns found within the “munnopsoid radiation” (containing the isopod families Munnopsidae Lilljeborg, 1864, Macrostylidae Hansen, 1916, Desmosomatidae Sars, 1897, and Nannoniscidae Hansen, 1916 among others) suggest an ancient invasion, probably during the early Permian, 232–314 mya (Lins et al., 2012), and subsequent radiation in the deep sea.

With more than 200 species in 32 genera known from all oceanic basins and a large proportion of species still waiting to be described, the Desmosomatidae and Nannoniscidae are particularly diverse and widespread. Although they are predominantly deep-sea taxa, several species have known occurrences on polar and temperate shelves (e.g., Brix & Svavarsson, 2010; Brix et al., 2015; Kaiser et al., 2009; Schiecke & Fresi, 1969; Schiecke & Modigh-Tota, 1976), whereas other species have only been reported from hadal depths (> 6000 m; Jennings et al., 2020).

Since the first description of a species of Nannoniscidae, *Nannoniscus oblongus* Sars, 1870, and its classification into the Desmosomatidae by Sars (1897), there has been little doubt about the close relationship of both families (Wägele, 1989). However, morphology-based concepts to thoroughly understand phylogenetic relationships between and within Desmosomatidae and Nannoniscidae are limited (e.g., Vanhöffen, 1914; Hessler, 1970; Siebenaller & Hessler, 1977; 1981; Svavarsson, 1984; Wägele, 1989, Kaiser & Brix, 2007; Wilson, 2008). In fact, it has been discussed whether both families should be combined into one, as strong apomorphies to separate both families were missing (Siebenaller & Hessler, 1977). For example, some genera, such as *Thaumastosoma* Hessler, 1970, *Ketosoma* Kaiser & Brix, 2018, and *Pseudomesus* Hansen, 1916, cannot be unambiguously assigned to either of the families, as they possess both nannoniscid and desmosomatid characters (Gurjanova, 1933; Hansen, 1916; Kaiser & Brix, 2007, 2007; Kaiser et al., 2018; Siebenaller & Hessler, 1977; Svavarsson, 1984; Wägele, 1989; Wilson, 2008). Furthermore, some characters have been revealed as inadequate to define family membership. For the Nannoniscidae, Wilson (2008) discussed the positioning of setae on either the coxa or tergite as a weak character for family assignment, as it is variable and plesiomorphic within the Janiroidea. Furthermore, the mandible subdistal tooth, considered as a synapomorphy for Nannoniscidae, is reduced in the nannoniscid genera *Thaumastosoma*, *Austroniscus* Vanhöffen, 1914 and *Exiliniscus* Siebenaller & Hessler, 1981 species, and it is also present in the Macrostylidae (Wilson, 2008). In the desmosomatid genera *Desmosoma* G.O. Sars, 1864, *Eugerda* Meinert, 1890 and *Mirabilicoxa* Hessler, 1970, on the other hand, composed setae on pereopod I (a desmosomatid synapomorphy) are reduced, whereas in the nannoniscid genus *Rapaniscus* Siebenaller & Hessler, 1981, composed setae are present. The composed (unequally bifid) seta is understood as strong seta with sensory function (see Hessler, 1970; Fig. 2b, p. 9).

Additionally, within-family relationships are not fully resolved by morphological means. For the Desmosomatidae, Hessler (1970) erected the two subfamilies Eugerdellatinae and Desmosomatinae using the shape of the first pereopod as the main character. Here, particularly the position of the genus *Torwolia* Hessler, 1970, is not entirely clear due to the peculiar subchelate condition of pereopod I (Hessler, 1970; but see Brix, 2007). In their morphological phylogenetic analyses, Riehl et al. (2014) used a number of characters that had not previously been considered to infer the phylogenetic relationships between the two families, including the male spermathecal duct position and position of the coxae of pereopods V-VII. Using these characters for desmosomatids and nannoniscids would imply that for each species both sexes are described. In the majority of species, this is not the case. In some genera, the sexual dimorphism can be strong as observed for *Mirabilicoxa* Hessler, 1970 (Golovan, 2018; Jennings et al., 2020).

Molecular studies to date have investigated relationships of Desmosomatidae and Nannoniscidae to other families within the munnopsoid clade with a limited taxon sampling (Lins et al., 2012; Raupach et al., 2004, 2009). Raupach (2004) found desmosomatids and nannoniscids to be monophyletic, together representing the sister-group of Macrostylidae. Raupach et al. (2009) placed the Desmosomatidae as the sister-group to the Nannoniscidae in their 50% majority rule consensus tree, while their strict consensus tree was inconclusive. Lins et al. (2012) included sequences from Raupach et al. (2004, 2009) and found the Nannoniscidae after the Macrostylidae appearing most basal in their tree with the Desmosomatidae forming the sister-group of a branch including Ischnomesidae Hansen, 1916, Janirellidae Menzies, 1956, Mesosignidae Schultz, 1969, and *Xostylus* Menzies, 1962 (Janiroidea *incertae sedis*). Furthermore, Brix et al. (2015, 2018, 2020), Kaiser et al. (2018), and Jennings et al. (2020) documented the phylogeny of a subset of taxa within Desmosomatidae and Nannoniscidae with material from different deep-sea regions of the world (South Atlantic, North Atlantic, North Pacific, and Central Pacific respectively) using molecular species delimitation. Yet, so far, no thorough systematic phylogenetic investigation of both families exists that includes most of the known supra-specific taxa. In particular, no sequence data of the “problematic” genera *Pseudomesus*, *Thaumastosoma*, or *Torwolia* have been included in a molecular phylogeny of the two families. Deciphering the phylogenetic position of these genera could lead to a re-evaluation and possibly new interpretation of characters used in the morphological phylogenetic literature to define Desmosomatidae and Nannoniscidae.

We comprehensively sampled the deep-sea families Desmosomatidae and Nannoniscidae to generate multilocus molecular (COI, 16S, and 18S) and morphological phylogenies, as well as a reanalysis of morphological characters to assess relationships between and within both families. Here, the question remains if the two families Desmosomatidae and Nannoniscidae can be separated, and if so, can valuable apomorphies to delimit them be identified? Some phenotypic features may have evolved independently more than once within the two families, likely driven by similarity of environmental settings and thus natural selection (e.g., Osborn, 2009). Recently it has been suggested that some cases of convergent evolution of phenotypic traits may have a genetic basis (Stern, 2013). Thus, putative cases of morphological homoplasy in this study may be the consequence of parallel genetic changes.

**Fig. 1:**
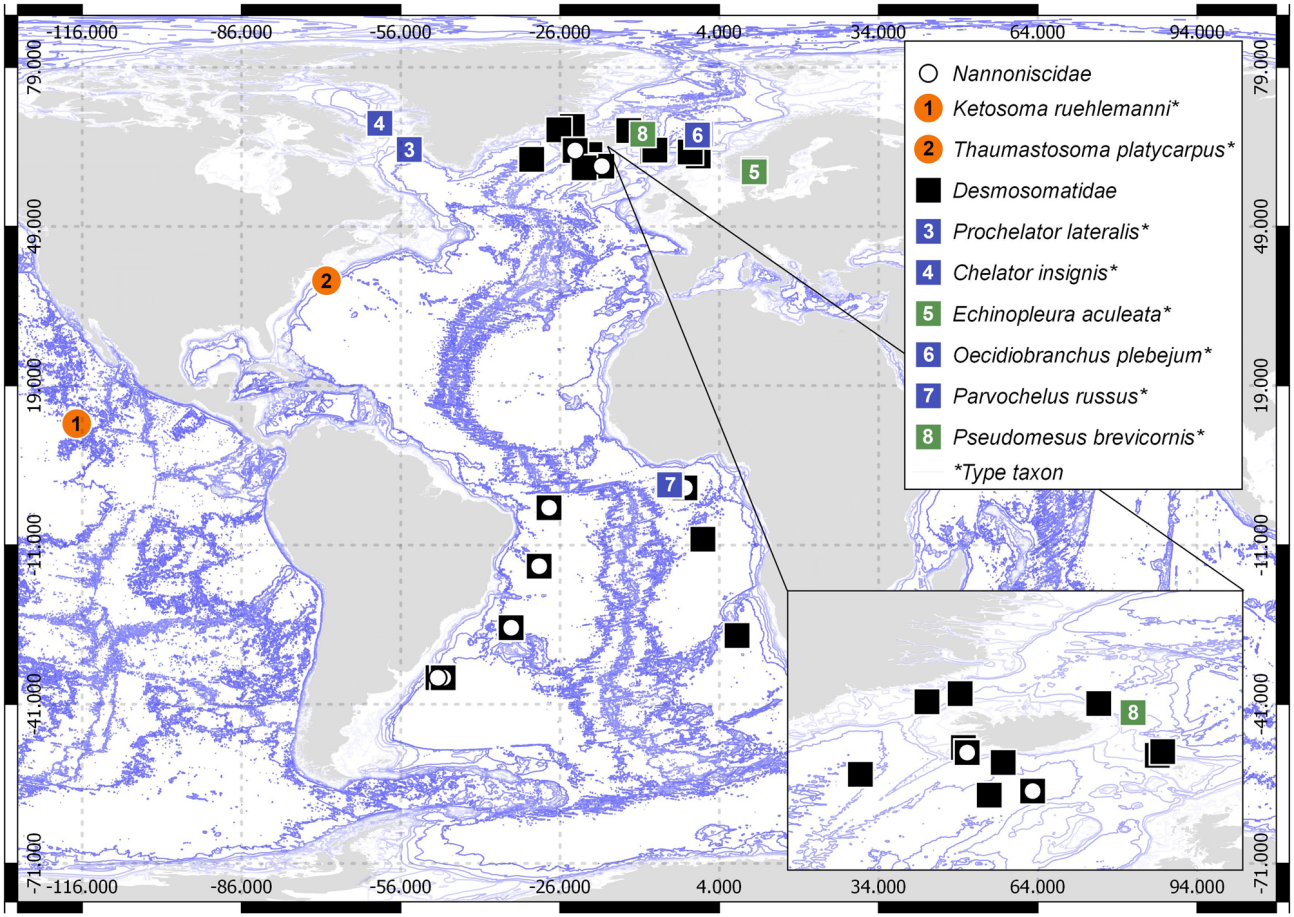
World map indicating sampling spots for the molecular dataset. White circles indicate nannoniscids in the samples, black squares desmosomatids in the samples. Orange dots with numbers indicate nannoniscid genera where sequences of the type species are available, green and blue squares with numbers indicate desmosomatid genera where sequences of the type species are available and included in the mirrored trees (see Fig. 8)

More specifically, we aimed to assess the monophyly of genera and subfamilies within Desmosomatidae and Nannoniscidae and to elucidate the systematic position of “problematic” genera (i.e., *Thaumastosoma*, *Pseudomesus*, and *Torwolia* in particular). Our data set comprises over 300 specimens collected from 14 ocean basins spanning the entire Atlantic Ocean and parts of the Pacific Ocean (Fig. 1). Hence, the large scope of this work allows the possibility of estimating divergence times between clades and diversification rates within them, to determine if they are regionally isolated within regions of the Atlantic, and possibly linked to historical forces (Eilertsen & Malaquias, 2015).

By applying multiple molecular and morphological approaches, we shed light on the diversity and phylogenetic relationships in two important isopod families, which should help to increase our understanding of mechanisms and drivers of evolutionary processes in the deep sea.

## Material and methods

### Genetics

Specimens for molecular analysis were obtained from seven cruises on which material was preserved in 96% ethanol to facilitate DNA extraction and amplification: DIVA-2 (M63/2 in 2005) and -3 (M72/1 in 2009), IceAGE-1 and -2 (M85/3 in 2011 and POS456 in 2013), the Vema-Transit cruise (S0237 in 2015), and ANDEEP-3 (PS 67/ANT XXII/3 in 2005) (Fig. 1, Table 1).

**Table 1:** List of all voucher specimens including information about BoLD field ID, morphological determination, Ocean Basin occurrence, and Genbank Accession number. More detailed information is available in the BoLD datasets linked to this study

				Genbank Accession number		
**Field ID**	**Taxonomy**	**Expedition**	**Ocean Basin**	**COI**	**18S**	**16S**
D2D001	*Mirabilicoxa* sp.	DIVA-2	CAP		MZ128360	
D2D003	*Chelator rugosus*	DIVA-2	CAP	KJ578686	KJ578678	KJ578667
D2D006	cf. *Mirabilicoxa*	DIVA-2	CAP		MZ128306	
D2D012	*Chelator rugosus*	DIVA-2	CAP	KJ578684		KJ578668
D2D020	*Eugerda* sp.	DIVA-2	GUI	MZ151154	MZ128357	
D2D022	cf. *Eugerda*	DIVA-2	GUI	MZ151099		
D2D023	*Chelator aequabilis*	DIVA-2	GUI	KJ578689		KJ578662
D2D029	cf. *Momedossa*	DIVA-2	GUI		MZ128361	
D2D031	*Parvochelus russus*	DIVA-2	GUI	KJ578695		KJ578671
D2D035	*Parvochelus russus*	DIVA-2	GUI	KJ578696		
D2D037	*Eugerda* sp.	DIVA-2	ANG		MZ128342	
D2D039	*Eugerdella theodori*	DIVA-2	GUI	MZ151102		
D2D041	*Eugerdella theodori*	DIVA-2	GUI	MZ151164		
D2D042	cf. *Mirabilicoxa*	DIVA-2	GUI	MZ151076	MZ128287	
D2D043	*Eugerdella theodori*	DIVA-2	GUI	MZ151096		
D2D044	*Parvochelus russus*	DIVA-2	GUI	KJ578697		KJ578672
D2D045	*Eugerdella theodori*	DIVA-2	GUI	MZ151089		
D2D048	cf. *Whoia*	DIVA-2	GUI	MZ151157	MZ128359	
D2D050	*Eugerdella theodori*	DIVA-2	GUI	KJ578699		KJ578673
D2D051	*Chelator aequabilis*	DIVA-2	GUI	KJ578690	KJ578675	KJ578663
D2D052	*Eugerda* sp.	DIVA-2	GUI		MZ128329	
D2D053	*Eugerdella huberti*	DIVA-2	GUI	HQ214677	KJ578682	HQ214679
D2D055	*Eugerdella* cf. *huberti*	DIVA-2	GUI	MZ151119		
D2D058	*Eugerdella theodori*	DIVA-2	GUI	MZ151115		
D2D061	*Parvochelus russus*	DIVA-2	GUI		MZ128303	
D2D062	*Eugerdella theodori*	DIVA-2	GUI	MZ151129		
D2D063	*Eugerdella theodori*	DIVA-2	GUI	MZ383786	KJ578680	
D2D064	*Eugerdella theodori*	DIVA-2	GUI	MZ383787	KJ578679	
D2D065	*Eugerdella huberti*	DIVA-2	GUI	HQ214678		
D2D074	*Eugerda* sp.	DIVA-2	GUI	MZ151162		
D2N004	*Nannoniscus* sp.	DIVA-2	GUI		MZ128300	
D2N008	*Exiliniscus* sp.	DIVA-2	GUI	MZ151092	MZ128301	
D2N011	*Nannoniscus* sp.	DIVA-2	GUI	MZ151148	MZ128350	
D2N013	*Whoia* sp.	DIVA-2	GUI	MZ151124	MZ128328	
D3D001	*Rapaniscus* sp.	DIVA-3	ARG		MZ128345	
D3D002	*Rapaniscus sp.*	DIVA-3	ARG	MZ151114		
D3D003	*Austroniscus* sp.	DIVA-3	ARG	MZ151090		MZ128190
D3D005	*Chelator* sp.	DIVA-3	ARG			MZ128222
D3D006	*Disparella* sp.	DIVA-3	ARG		MZ128341	
D3D007	*Familia nova*	DIVA-3	ARG			MZ128267
D3D008	*Rapaniscus* sp.	DIVA-3	ARG	MZ151163		
D3D009	*Austroniscus* sp.	DIVA-3	ARG		MZ128299	MZ128192
D3D012	cf. *Desmosoma*	DIVA-3	ARG			MZ128273
D3D013	*Familia nova*	DIVA-3	ARG			MZ128184
D3D018	cf. *Nannoniscoides*	DIVA-3	ARG		MZ128308	
D3D019	*Rapaniscus* sp.	DIVA-3	ARG	MZ151146		
D3D020	*Disparella* sp.	DIVA-3	ARG			MZ128176
D3D030	*Austroniscus* sp.	DIVA-3	ARG	MZ151128	MZ128333	MZ128240
D3D035	cf. *Desmosoma*	DIVA-3	ARG			MZ128171
D3D038	cf. *Mirabilicoxa*	DIVA-3	ARG	MZ151137		
D3D043	*Mirabilicoxa* sp.	DIVA-3	ARG			MZ128228
D3D045	*Mirabilicoxa* sp.	DIVA-3	ARG	MZ151159		
D3D047	cf. *Desmosoma*	DIVA-3	ARG	MZ151079		
D3D051	*Austroniscus* sp.	DIVA-3	ARG	MZ151108	MZ128315	MZ128210
D3D053	*Austroniscus* sp.	DIVA-3	ARG		MZ128349	
D3D054	*Nannoniscus* sp.	DIVA-3	ARG	MZ383788	MZ379978	
D3D055	cf. *Eugerdella* cf. *cornuta*	DIVA-3	ARG		MZ128358	MZ128280
D3D060	*Ketosoma werneri*	DIVA-3	ARG	MF040893	KY951738	
D3D061	cf. *Parvochelus*	DIVA-3	ARG		MZ128305	
D3D063	*Regabellator* sp.	DIVA-3	ARG	MZ151088	MZ128297	MZ128187
D3D064	*Thaumastosoma diva*	DIVA-3	ARG		KY951739	KY951731
D3D066	*Mirabilicoxa* sp.	DIVA-3	ARG		MZ128313	MZ128209
D3D067	cf*. Eugerdella* cf. *cornuta*	DIVA-3	ARG		MZ128307	MZ128200
D3D068	*Mirabilicoxa* sp.	DIVA-3	ARG	MZ151081	MZ128293	
D3D069	cf. *Desmosoma*	DIVA-3	ARG		MZ128304	MZ128196
D3D070	*Mirabilicoxa* sp.	DIVA-3	ARG		MZ128296	MZ128186
D3D071	*Mirabilicoxa* sp.	DIVA-3	ARG	MZ151101	MZ128309	MZ128204
D3D072	*Mirabilicoxa* sp.	DIVA-3	ARG		MZ128347	MZ128263
D3D073	cf. *Desmosoma*	DIVA-3	BRA		MZ128312	MZ128208
D3D074	*Eugerdella* sp.	DIVA-3	BRA		MZ128348	MZ128266
D3D081	*Rapaniscus* sp.	DIVA-3	BRA	MZ151104		MZ128206
D3D082	*Disparella* sp.	DIVA-3	BRA	MZ128370	MZ128391	MZ128376
D3D083	*Exiliniscus* sp.	DIVA-3	BRA		MZ128324	MZ128232
D3D086	*Mirabilicoxa* sp.	DIVA-3	BRA	MZ151116		MZ128224
D3D088	*Pseudomesus* sp.	DIVA-3	BRA	MZ151080	MZ128292	MZ128174
D3D099	*Prochelator* sp.	DIVA-3	BRA	MZ151082	MZ128294	MZ128175
D3D100	*Disparella* sp.	DIVA-3	BRA	MZ128364	MZ128387	MZ128372
D3D104	*Mirabilicoxa* sp.	DIVA-3	BRA	MZ128368		MZ128374
D3D105	*Mirabilicoxa* sp.	DIVA-3	BRA	MZ128363	MZ128386	MZ128371
D3D106	*Mirabilicoxa* sp.	DIVA-3	BRA	MZ151136	MZ128339	MZ128251
D3D108	*Chelator* sp.	DIVA-3	BRA	MZ151135		MZ128249
D3D110	*Whoia* sp.	DIVA-3	BRA	MZ151077	MZ128288	
D3D111	*Eugerdella* sp.	DIVA-3	BRA		MZ128352	MZ128270
D3D112	cf. *Eugerda*	DIVA-3	BRA			MZ128230
D3D113	cf. *Desmosoma*	DIVA-3	BRA	MZ151120		
D3D115	*Eugerdella* sp.	DIVA-3	BRA	MZ151107		
D3D116	*Eugerdella* sp.	DIVA-3	BRA	MZ151110	MZ128316	MZ128213
D3D117	*Eugerdella* sp.	DIVA-3	BRA	MZ151142	MZ128344	MZ128259
D3D118	*Prochelator* sp.	DIVA-3	BRA		MZ128335	MZ128243
D3D121	*Chelator* sp.	DIVA-3	BRA	MZ151144	MZ128346	MZ128261
D3D123	*Prochelator* sp.	DIVA-3	BRA	MZ151121		
D3D125	*Exiliniscus* sp.	DIVA-3	BRA			MZ128215
D3D126	*Mirabilicoxa* sp.	DIVA-3	BRA	MZ151160		
D3D130	*Eugerdella* sp.	DIVA-3	BRA	MZ151134		
D3D138	cf. *Desmosoma*	DIVA-3	BRA	MZ151151		
D3D140	*Exiliniscus* sp.	DIVA-3	BRA	MZ151123	MZ128327	MZ128234
D3D141	*Exiliniscus* sp.	DIVA-3	BRA	MZ151143		
D3D142	*Hebefustis* sp.	DIVA-3	BRA	MZ151106		
D3D143	*Prochelator* sp.	DIVA-3	BRA		MZ128340	MZ128252
D3D146	*Regabellator* sp.	DIVA-3	BRA		MZ128290	MZ128172
D3D148	*Mirabilicoxa* sp.	DIVA-3	BRA	MZ151097		
D3D149	cf. *Eugerda*	DIVA-3	BRA		MZ128310	MZ128205
D3D150	*Mirabilicoxa* sp.	DIVA-3	BRA		MZ128355	MZ128274
D3D152	*Mirabilicoxa* sp.	DIVA-3	BRA	MZ128369	MZ128390	MZ128375
D3D153	*Mirabilicoxa* sp.	DIVA-3	BRA	MZ151130	MZ128334	MZ128241
D3D154	*Chelator* sp.	DIVA-3	BRA	MZ151139	MZ128343	MZ128255
D3D155	*Mirabilicoxa* sp.	DIVA-3	BRA		MZ128336	MZ128244
D3D156	*Parvochelus russus*	DIVA-3	BRA	KJ578694		MZ128197
D3D157	*Parvochelus russus*	DIVA-3	BRA	KJ578698	KJ578674	MZ128278
D3D158	*Disparella* sp.	DIVA-3	BRA	MZ128367	MZ128389	MZ128373
D3D159	*Eugerdella* sp.	DIVA-3	BRA		MZ128289	MZ128169
D3D160	*Eugerdella* sp.	DIVA-3	BRA			MZ128242
D3D161	*Eugerdella* sp.	DIVA-3	BRA		MZ128320	MZ128220
D3D163	*Eugerdella* sp.	DIVA-3	BRA		MZ128318	MZ128218
D3D165	*Eugerdella* sp.	DIVA-3	BRA	MZ151152	MZ128354	MZ128272
D3D166	*Eugerda* sp.	DIVA-3	BRA		MZ128314	
D3D168	*Exiliniscus* sp.	DIVA-3	BRA	MZ151103	MZ128311	
D3D169	*Exiliniscus* sp.	DIVA-3	BRA	MZ151132	MZ128338	MZ128245
D3D170	*Exiliniscus* sp.	DIVA-3	BRA	MZ151149	MZ128351	MZ128269
D3D171	*Exiliniscus* sp.	DIVA-3	BRA	MZ151150	MZ128353	MZ128271
IA2Desm01	*Oecidiobranchus otu3*	IceAGE2	NCH	MZ383789		MG895881
IA2Desm02	*Oecidiobranchus* cf. *nanseni*	IceAGE2	NCH	MG831409		MG895894
IA2Desm03	*Oecidiobranchus otu3*	IceAGE2	FIR	MG831399		MG895880
IDesm001	*Mirabilicoxa* sp.	IceAGE1	ICE	MZ151078		MZ128168
IDesm002	*Mirabilicoxa* sp.	IceAGE1	ICE			MZ128256
IDesm003	*Mirabilicoxa* sp.	IceAGE1	ICE	MZ151094	MZ128302	MZ128194
IDesm004	*Mirabilicoxa *cf*. similis*	IceAGE1	ICE	MZ151125		MZ128235
IDesm008	*Eugerda* cf. *reticulata*	IceAGE1	ICE			MZ128216
IDesm010	*Thaumastosoma* cf. *platycarpus*	IceAGE1	ICE	MF040897	KY951740	KY951735
IDesm012	*Thaumastosoma* cf. *platycarpus*	IceAGE1	ICE	MF040896		KY951734
IDesm013	*Mirabilicoxa *cf*. acuminata*	IceAGE1	ICE	MZ151117		MZ128225
IDesm014	*Chelator insignis*	IceAGE1	ICE	KJ710289	KJ630816	KJ630813
IDesm015	*Chelator insignis*	IceAGE1	ICE	KJ710302	KJ630817	KJ937325
IDesm016	*Pseudomesus* cf. *brevicornis*	IceAGE1	ICE	MZ151165		MZ128285
IDesm017	*Mirabilicoxa* sp.	IceAGE1	ICE	MZ151109		MZ128212
IDesm018	*Mirabilicoxa* cf. *longispina*	IceAGE1	ICE	MZ151127		MZ128237
IDesm019	*Mirabilicoxa* cf. *gracilipes*	IceAGE1	ICE	MZ151155		MZ128277
IDesm022	cf. *Mirabilicoxa*	IceAGE1	ICE	MZ151156		MZ128279
IDesm023	cf. *Mirabilicoxa*	IceAGE1	ICE	MZ151095		MZ128195
IDesm024	cf. *Mirabilicoxa*	IceAGE1	ICE	MZ151093		MZ128193
IDesm028	*Mirabilicoxa* sp.	IceAGE1	ICE	MZ151161		MZ128282
IDesm030	*Eugerda* cf. *reticulata*	IceAGE1	ICE			MZ128202
IDesm032	cf. *Mirabilicoxa*	IceAGE1	ICE	MZ151138		MZ128254
IDesm033	*Chelator insignis*	IceAGE1	ICE			MZ379981
IDesm034	*Prochelator lateralis*	IceAGE1	ICE			MZ351257
IDesm035	*Chelator insignis*	IceAGE1	ICE	KJ710278	KJ630818	KJ630812
IDesm038	*Chelator insignis*	IceAGE1	ICE	KJ710294		KJ630811
IDesm039	*Chelator* cf. *insignis*	IceAGE1	ICE			KJ937311
IDesm041	*Thaumastosoma *cf*. platycarpus*	IceAGE1	ICE	MF040895		KY951733
IDesm042	*Chelator vulgaris*	IceAGE1	ICE	KJ710288	KJ630819	MZ379982
IDesm045	*Thaumastosoma* cf. *platycarpus*	IceAGE1	ICE	MF040894		KY951732
IDesm046	*Thaumastosoma* cf. *platycarpus*	IceAGE1	ICE	MF040898		KY951736
IDesm047	*Eugerdella* cf. *armata*	IceAGE1	ICE	MZ151084		MZ128180
IDesm049	*Eugerda *cf*. reticulata*	IceAGE1	ICE			MZ128276
IDesm052	*Eugerda *sp. 2	IceAGE1	ICE	MZ151085		MZ128181
IDesm054	*Chelator insignis*	IceAGE1	REY	KJ710304		KJ630808
IDesm057	*Echinopleura aculeata*	IceAGE1	REY			MZ128182
IDesm058	*Chelator insignis*	IceAGE1	ICE	KJ710306	KJ630820	KJ630815
IDesm075	*Oecidiobranchus* cf. *nanseni*	IceAGE1	IRM	MG831406		MG895890
IDesm078	*Eugerda* cf. *tenuimana*	IceAGE1	IRM	MZ151158		MZ128281
IDesm082	*Pseudomesus* sp.	IceAGE1	DEN			MZ128260
IDesm083	*Pseudomesus* sp.	IceAGE1	DEN			MZ128199
IDesm085	*Eugerda *sp. 3	IceAGE1	DEN	MZ151141		MZ128258
IDesm095	*Chelator insignis*	IceAGE1	ICE	KJ710284	KJ630822	KJ937317
IDesm100	*Chelator insignis*	IceAGE1	ICE	KJ710285	KJ630823	KJ937318
IDesm115	*Chelator insignis*	IceAGE1	ICE	KJ710312	MZ379979	KJ937333
IDesm131	*Chelator insignis*	IceAGE1	ICE		KJ630824	KJ937312
IDesm132	*Mirabilicoxa* sp.	IceAGE1	IRM			MZ128170
IDesm133	*Mirabilicoxa* sp.	IceAGE1	IRM			MZ128250
IDesm136	*Chelator insignis*	IceAGE1	REY	KJ710283	MZ379980	KJ937316
IDesm158	*Oecidiobranchus* cf. *plebejum*	IceAGE1	NOR	MG831394		MG895874
IDesm161	*Oecidiobranchus* cf. *plebejum*	IceAGE1	NOR	MG831392	MG936645	MG895872
IDesm162	*Oecidiobranchus* cf. *plebejum*	IceAGE1	NOR	MG831391	MG936644	MG895871
IDesm170	*Pseudomesus brevicornis*	IceAGE1	NOR			MZ128198
IDesm173	*Echinopleura aculeata*	IceAGE1	REY	MZ151113	MZ128319	MZ128219
IDesm180	*Chelator insignis*	IceAGE1	ICE	KJ937306	KJ630826	MZ379983
IDesm183	*Chelator insignis*	IceAGE1	REY	KJ937308	KJ630828	MZ379984
IDesm187	*Mirabilicoxa* sp.	IceAGE1	DEN			MZ128178
IDesm190	*Thaumastosoma platycarpus*	IceAGE1	ICE			MZ128226
IDesm191	*Prochelator lateralis*	IceAGE1	REY	MZ151140		MZ128257
IDesm192	*Prochelator lateralis*	IceAGE1	REY			MZ128284
IDesm193	*Pseudomesus brevicornis*	IceAGE1	ICE	MZ151083		MZ128177
IDesm195	*Pseudomesus brevicornis*	IceAGE1	ICE			MZ128229
IDesm204	*Chelator insignis*	IceAGE1	ICE	KJ937303		MZ379985
IDesm206	cf. *Mirabilicoxa*	IceAGE1	ICE			MZ128246
INann39	*Austroniscus* cf. *groenlandicus*	IceAGE1	ICE	MZ151074		MZ128166
INann40	*Pseudomesus* sp.	IceAGE1	ICE			MZ128253
INann43	*Pseudomesus* sp.	IceAGE1	ICE			MZ128188
KJ277	*Prochelator lateralis*	Oslo Fjord	OSF		MZ128325	
KJ280	*Prochelator lateralis*	Oslo Fjord	OSF		MZ128331	MZ128238
KJ281	*Prochelator lateralis*	Oslo Fjord	OSF			MZ128268
KJ288	*Echinopleura* cf. *aculeata*	Oslo Fjord	OSF		MZ128298	
KJ291	*Prochelator lateralis*	Oslo Fjord	OSF		MG936646	
KJ292	*Prochelator lateralis*	Oslo Fjord	OSF		MZ128337	MZ379986
DE1	*Chelator* sp.	ANDEEP	SO	KJ578691	AY461460	
DE2	*Mirabilicoxa* sp.	ANDEEP	SO		AY461461	
DE4	*Prochelator* sp.	ANDEEP	SO	MZ337818	AY461462	
DE7	*Eugerda* sp.	ANDEEP	SO		AY461463	
KM14_Iso259_1	*Ketosoma* sp. nov. 2	MANGAN	CCZ		KY693694	KY693698
KM14_Iso261_2	*Ketosoma* sp. nov. 2	MANGAN	CCZ		KY693695	KY693697
NB12_Iso740_9	*Ketosoma* sp. nov. 1	MANGAN	CCZ		KY693696	
NBIso337	*Ketosoma ruehlemanni*	MANGAN	CCZ	KJ736158		
VTDes001	*Disparella* sp.	VEMA-TRANSIT	VEM	MF325479	MF325728	MF325639
VTDes007	*Torwolia* sp.	VEMA-TRANSIT	VEM	MF325577	MF325781	MF325692
VTDes008	*Pseudomesus* sp.	VEMA-TRANSIT	VEM	MF325557	MF325770	MF325684
VTDes011	*Eugerdella* sp.	VEMA-TRANSIT	VEM	MF325489	MF325735	
VTDes012	*Eugerdella* sp.	VEMA-TRANSIT	VEM	MF325490	MF325736	
VTDes013	*Ketosoma vemae*	VEMA-TRANSIT	VEM	MF040892	KY951737	KY951730
VTDes014	*Whoia* sp.	VEMA-TRANSIT	VEM	MF325578	MF325782	
VTDes019	*Pseudomesus* sp.	VEMA-TRANSIT	VEM	MF325554	MF325768	MF325681
VTDes024	*Torwolia* sp.	VEMA-TRANSIT	VEM	MF325576	MF325780	MF325691
VTDes031	*Parvochelus* sp.	VEMA-TRANSIT	VEM	MF325537	MF325756	MF325671
VTDes033	*Chelator* sp.	VEMA-TRANSIT	VEM	MF325441	MF325707	MF325604
VTDes036	*Disparella* sp.	VEMA-TRANSIT	VEM	MF325478	MF325727	
VTDes108	*Prochelator barnacki*	VEMA-TRANSIT	VEM	MF325543	MF325760	
VTDes112	*Prochelator* sp.	VEMA-TRANSIT	VEM	MF325545	MF325761	
VTDes159	*Torwolia* sp.	VEMA-TRANSIT	VEM	MF325575	MF325779	MF325690
VTDes161	*Eugerdella* sp.	VEMA-TRANSIT	VEM	MF325484	MF325732	
VTDes569	*Ketosoma hessleri*	VEMA-TRANSIT	VEM			KY951729

Before DNA extraction, all isopod specimens were morphologically identified and given individual voucher numbers. All voucher specimens are stored at the Zoological Museum, Hamburg (LIB - Leibnitz Insitute for the Analysis of Biodiversity Change; Zoological Museum, Hamburg; see Table 1). After DNA extraction, all isopod specimens were re-checked morphologically to species level using a LEICA MZ 12.5 stereomicroscope and thus molecular trees were quality checked and cross-checked with the morphological identifications. All determinations were entered into an Excel spreadsheet to use as a baseline for creating maps in QGIS.

Three markers were selected for analysis: the nuclear small ribosomal subunit (18S), and the mitochondrial cytochrome *c* oxidase subunit I (COI) and large ribosomal subunit (16S). We chose a set of one nuclear gene and two mitochondrial genes because they are widely used in deep-sea isopod phylogenetic studies (Brix et al., 2014, 2015; Kaiser et al., 2018; Lins et al., 2012; Osborn, 2009; Raupach et al., 2007, 2009; Riehl et al., 2014) and allow for integration with and comparison to existing data. DNA extraction, PCR, and sequencing were as described in Riehl et al. (2014). Sequencing of all loci was performed at the Smithsonian Institute’s Laboratories of Analytical Biology (LAB) as described in Riehl et al. (2014). Additionally, these protocols were applied in the laboratory of the University of Hamburg with material from the Oslo Fjord sampled in 2014.

Sequences were checked by hand using the Geneious software (Biomatters Ltd.) to remove primer regions and regions of low confidence, to resolve mismatches, and to check for proper amino acid translation (COI). These quality-checked sequences were screened for contaminants by BLAST searches against the GenBank nucleotide database; verified sequences were deposited in GenBank (Table 1). All specimen and sequence information including metadata is available under the BoLD dataset DEEPISO under 10.5883/DS-DEEPISO.  For COI, sequences were aligned as DNA codons using the CLUSTAL algorithm (Larkin et al., 2007) in BioEdit (Tom Hall, Ibis Therapeutics) with default settings. The 16S and 18S alignments were produced with MAFFT ver. 7 (Katoh & Standley, 2013) using default settings, followed by removal of poorly aligned regions in the online Gblocks v0.91b server (Talavera et al., 2007) using all three options for a less stringent selection. Outgroups were chosen from GenBank or in-house unpublished data based on phylogenetic proximity and availability: three to four randomly chosen representatives from Macrostylidae, Haploniscidae Hansen, 1916, and Munnopsidae. The final alignments were deposited in DRYAD under https://doi.org/10.5061/dryad.9w0vt4bfp.

Aligned sequences were used to estimate phylogenetic trees separately for each locus using Bayesian phylogenetic (BP) algorithms in BEAST 2.4.1 (Bouckaert et al., 2014) using the GTR nucleotide substitution model, with four gamma-distributed categories of rate heterogeneity and estimated equilibrium nucleotide frequencies. A starting tree computed via UPGMA and a Yule process of tree evolution was employed. For COI and 18S, branch rate heterogeneity was modeled with a relaxed uncorrelated lognormal clock; for 16S, branch rate heterogeneity could not be adequately modeled, so a strict clock was employed. All tree computations were started with 10 million steps, then checked with Tracer 1.6 and run further if needed until all effective sample size (ESS) estimates were ≥ 200 with a manually chosen burn-in. Final Bayesian trees were computed using TreeAnnotator, with maximum clade credibility tree using common ancestor heights. Multilocus trees were computed on a reduced dataset comprising all specimens for which sequences were obtained from any two of the three loci (the “2G” dataset). Outgroup sequences from single-locus datasets were combined and included if taxonomic IDs across loci were identical at the conspecific level, or if this was not possible at the congeneric level. The Bayesian 2G tree was computed in BEAST2 as above, with site and clock models unlinked across loci.

To estimate divergence times for clades in the molecular trees, divergence estimates from Lins et al. (2012) were used as calibration points in the 2G Bayesian tree, employing normally distributed priors with means taken from Fig. 1 (pg. 980). The divergence of Haploniscidae (our outgroup) was placed at 310 mya and given a variance of 60 mya to correspond to the 95% credibility interval of Lins et al. (2012). The divergence of Nannoniscidae was placed at 260 mya; and the divergence of Desmosomatidae was placed at 210 mya; because no credibility intervals were available for these latter dates, variances of 60 mya were applied here as well. The resulting calibrated 2G tree was used to perform lineage through time (LTT) analysis with the “speciationextinction” model in BAMM (Rabosky, 2014), to determine if significant changes in speciation and extinction rates have occurred in these taxa. Initial values for priors were selected empirically using setBammpriors, a function in the companion BAMMtools package in R. Five million Markov chain steps were employed, with four heated chains (Metropolis coupling); a deltaT of 0.1 (lowest chain 77% heating) was selected to promote mixing among chains while maintaining the suggested acceptance rates. The expected number of rate shifts was varied among runs from 1 to 3. The first 10% of each run was excluded as burn-in, and BAMMtools was used to ensure the effective sample size (ESS) of the remaining steps was > 200. Functions in BAMMtools were used to analyze the output file and produce estimates and confidence parameters as described in the online documentation and guide. For comparison, speciation and extinction rates were estimated using the TESS package (Hoehna et al., 2015) in R, with hyper-parameters estimated empirically, a fraction 0.75 of unsampled lineages among Desmosomatidae and Nannoniscidae, and the MCMC chain run until the ESS reached 500. Replicate runs were conducted with normally distributed priors, and with lognormally distributed priors. Assessment of convergence and generation of output plots were conducted in R according to suggestions in the TESS manual, and the run configuration with the best convergence statistics was chosen. The R package phytools was also used to test the fit of simple models including speciation only (the Yule model) vs. speciation and extinction (the birth–death model).

To estimate the number of species (or Operational Taxonomic Units, OTUs) in the molecular datasets, species delimitation (SD) analyses were conducted on the full COI and 16S ingroup datasets (18S has too slow a mutation rate, and the 2G dataset included too few taxa with enough putative species lineages). Three analyses were conducted on each dataset: ABGD (Automatic Barcode Gap Detection, Puillandre et al., 2011), single-threshold GMYC (General Mixed Yule Coalescent, Pons et al., 2006), and mPTP (multiple Poisson Tree Process, Kapli et al., 2016). The ABGD analysis was performed on aligned sequences using the online website (https://bioinfo.mnhn.fr/abi/public/abgd/abgdweb.html) using K2P distance. GMYC and mPTP were performed on the Bayesian trees from BEAST2; GMYC was performed using its R package, and mPTP with the command-line software, with 3 replicate runs of 100 million steps, discarding the first 1% as burn-in.

### Morphology

**Fig. 2:**
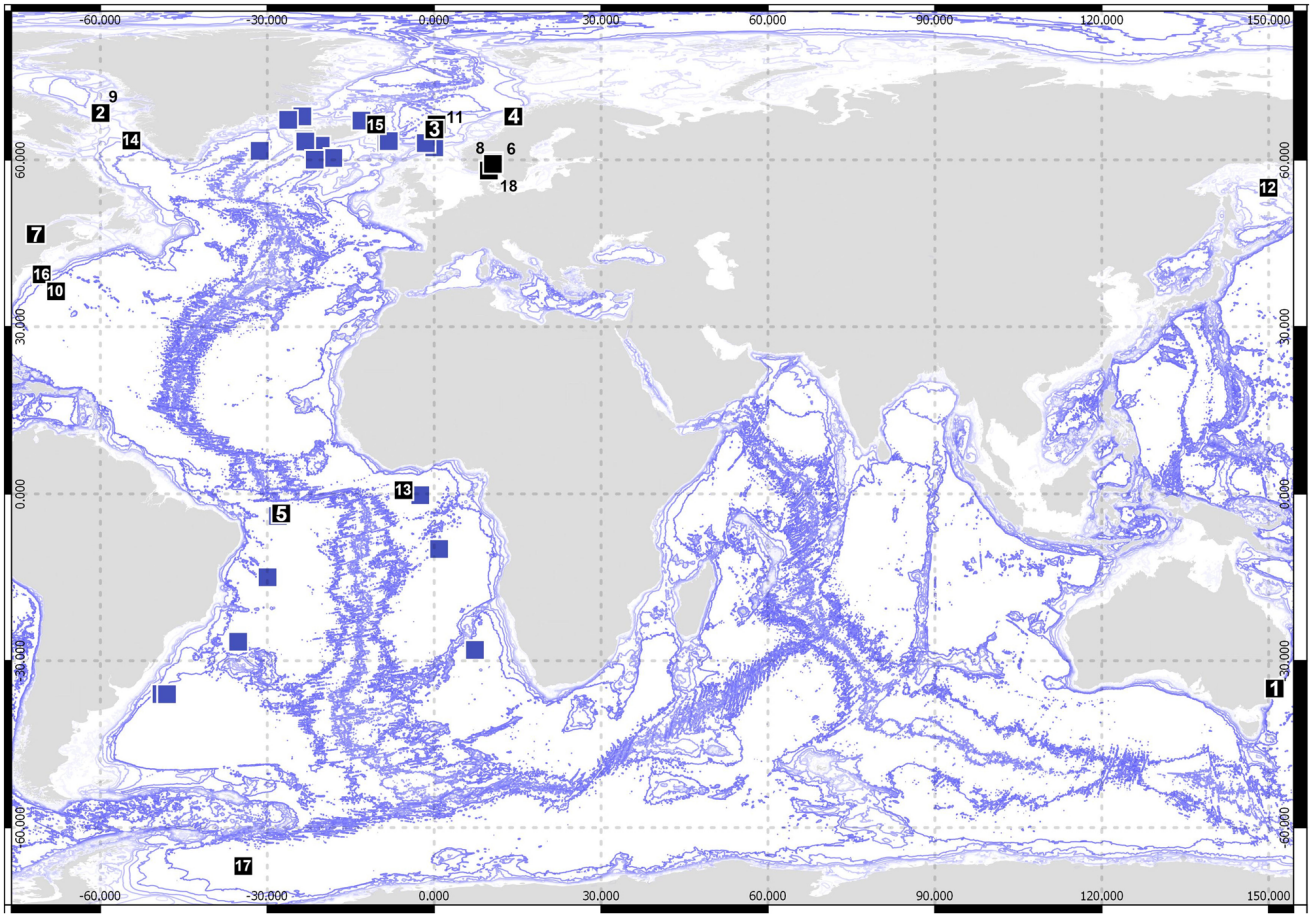
Type localities of type species of desmosomatid genera. The blue squares reflect the genetic dataset available in this study (compare Fig. 1). 1—*Chelantermedia composita* Brix, 2007, 2—*Chelator insignis* (Hansen, 1916), 3—*Cryodesma agnari* Svavarsson, 1988, 4—*Desmosoma lineare* G.O. Sars 1864, 5—*Disparella valida* Hessler, 1970, 6—*Echinopleura aculeata* (G.O. Sars, 1864), 7—*Eugerda tenuimana* (G.O. Sars, 1866), 8—*Eugerdella coarctata* (G.O. Sars, 1899), 9—*Mirabilicoxa gracilipes* (Hansen, 1916), 10—*Momedossa profunda* Hessler, 1970, 11—*Oecidiobranchus plebejum* (Hansen, 1916), 12—*Paradesmosoma conforme* (Kussakin, 1965), 13—*Parvochelus russus* Brix & Kihara, 2015, 14—*Prochelator lateralis* (G.O.Sars, 1899), 15—*Pseudomesus brevicornis* (Hansen, 1916), 16—*Reductosoma gunnera* Brandt, 1992, 17—*Torwolia subchelatus* Hessler, 1970, 18—*Whoia angusta* (G.O.Sars, 1899)

**Fig. 3:**
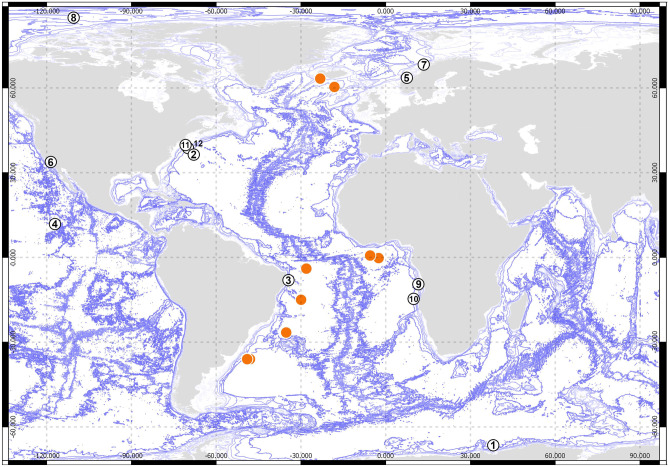
Type localities of type species of nannoniscid genera. The orange dots reflect the genetic dataset available in this study (compare Fig. 1). 1—*Austroniscus ovalis* (Vanhöffen, 1914), 2—*Exiliniscus clipeatus* Siebenaller & Hessler, 1981, 3—*Ketosoma ruehlmanni* Kaiser & Janssen, 2018, 4—*Hebefustis vafer* Siebenaller & Hessler, 1981, 5—*Nannoniscoides angulatus* (Hansen, 1916), 6—*Nannoniscus oblongus* (G.O. Sars, 1870), 7—*Nannonisconus latipleonus* (Schultz, 1966), 8—*Nymphodora fletcheri* (Paul & George, 1975), 9—P*anetela wolffi* Siebenaller & Hessler, 1981, 10—*Rapaniscus dewdenyi* Sienbenaller & Hessler, 1981, *Regabellator profugus* Siebenaller & Hessler, 1981, 12—*Thaumastosoma platycarpus* Hessler, 1970

To be included in the morphological phylogenetic analysis, specimens had to be assignable to described species. The material examined was sampled during the scientific cruises DIVA-1 (Latitudinal Gradients of deep-sea BioDIVersity in the Atlantic Ocean) with RV *Meteor* in summer 2000, ANDEEP I–II (ANtarctic benthic DEEP-sea biodiversity, colonization history, and recent community patterns) in Antarctic spring 2002. Additionally, type material from the following museums was studied: Australian Museum, Sydney (AM); United States National Museum of Natural History, Washington D.C. (USNM); Zoological Museum of the University of Copenhagen (ZMUC); Museum für Naturkunde, Berlin and Zoological Museum, Hamburg (ZMH) (a detailed list of type specimens used is available as Electronic Supplement 1). Type localities of the species included in the morphological tree are illustrated per family in Figs. 2 and 3.

Maps were created using QGIS version 2.16 based on distribution data available in OBIS, GBIF, and all literature available summarized in the so called “Asselkartei” literature collection of Johann-Wolfgang Wägele (currently housed in Bonn and accessed by SB January 2020).

For the morphological analysis, the eight steps of a complete phylogenetic analysis presented by Wägele (2004) were followed. Wägele (2004) adopted the Hennigian method of modern cladistics. Although the basis of our phylogenetic approach is the Hennigian method, we highlight that we follow the methodology described by Wägele (2004) as “phylogenetic cladistics”, i.e., a further development of the Hennigian method. This includes, for example, “traditional” steps like the “a priori” analysis (i.e., the character discussion, see Electronic Supplement 2) and character weighting as well as the use of computer programs.

The morphological phylogenetic analysis was based on a character matrix (Table 2) established with the program DELTA (Description Language for Taxonomy, DELTA Editor, 1.04, © CSIRO 1998–2000, Dallwitz, 1980; Dallwitz et al., 1999) and NEXUSEDITOR (version 0.5.0 © Roderic D.M. Page, University of Glasgow, 2001). PAUP (Swofford, 1998: Phylogenetic Analysis Using Parsimony) was used to conduct the analysis (*ß* test version 4.0b10 for Windows) after converting the DELTA matrix into a nexus file. The DELTA matrix contains 107 taxa and 129 characters. To distinguish the outgroup from the ingroup, 12 characters were used. The character matrix concentrates on highly complex characters, which are hypothesized to be phylogenetically informative. Macrostylidae are defined as the outgroup because they are regarded as closely related to Desmosomatidae and Nannoniscidae, but are clearly differentiated from them by more than 10 synapomorphies (Riehl et al., 2014; Wägele, 1989). The choice of Macrostylidae as outgroup, and its systematic position relative to the ingroups (Desmosomatidae and Nannoniscidae) is based on work by Wägele (1989) and Raupach et al., (2004, 2009). From both morphological and molecular genetic analyses, there is consensus in choosing Macrostylidae as the outgroup (see above), although there are differences in the systematic position of Macrostylidae and Munnopsidae. In the molecular study of Lins et al. (2012), munnopsids were the sister taxon to desmosomatids while in the morphological analysis, macrostylids were the sister taxon. This placement makes macrostylids an ideal choice as outgroup.

**Table 2:**
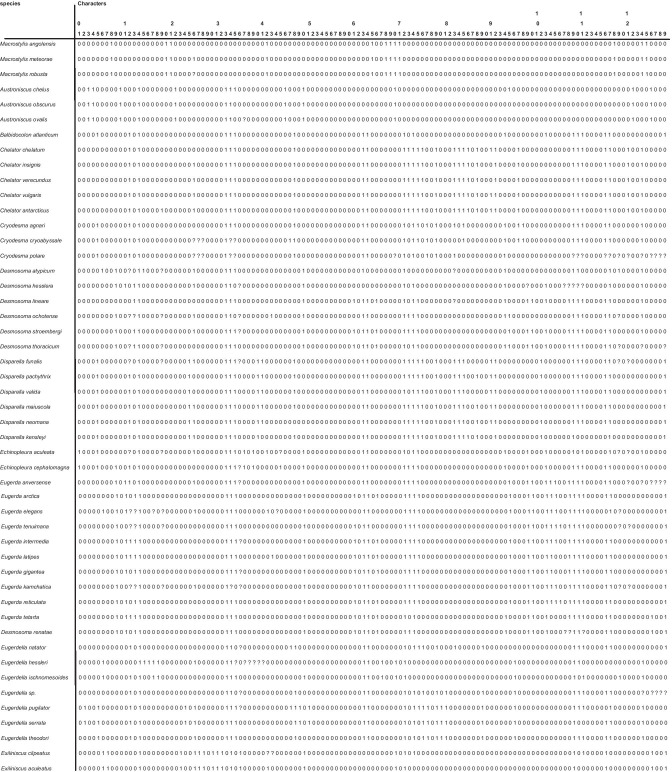
DELTA morphological phylogenetic analysis was based on a character matrix


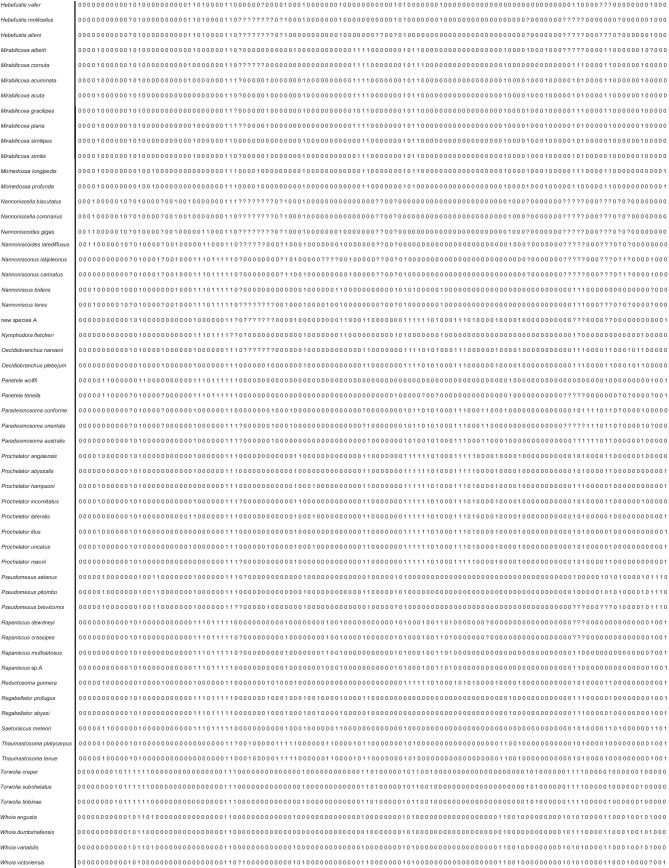
Characters of Nannoniscidae and Desmosomatidae were treated equally and analyzed as one group. Characters of sexual dimorphism were not used within the phylogenetic analysis because males and females are not known for all species. For the phylogeny, only adult specimens or preparatory females are described in detail. A list of all characters and their a priori weighting sensu Wägele (2004) is presented in Electronic Supplement 3. We followed Richter (2005) in using character weighting by splitting characters into subcharacters according to Wägele (2004) as long as the substructures are tested for homology, as was done in our character analysis (Electronic Supplement 2).

**Fig. 4:**
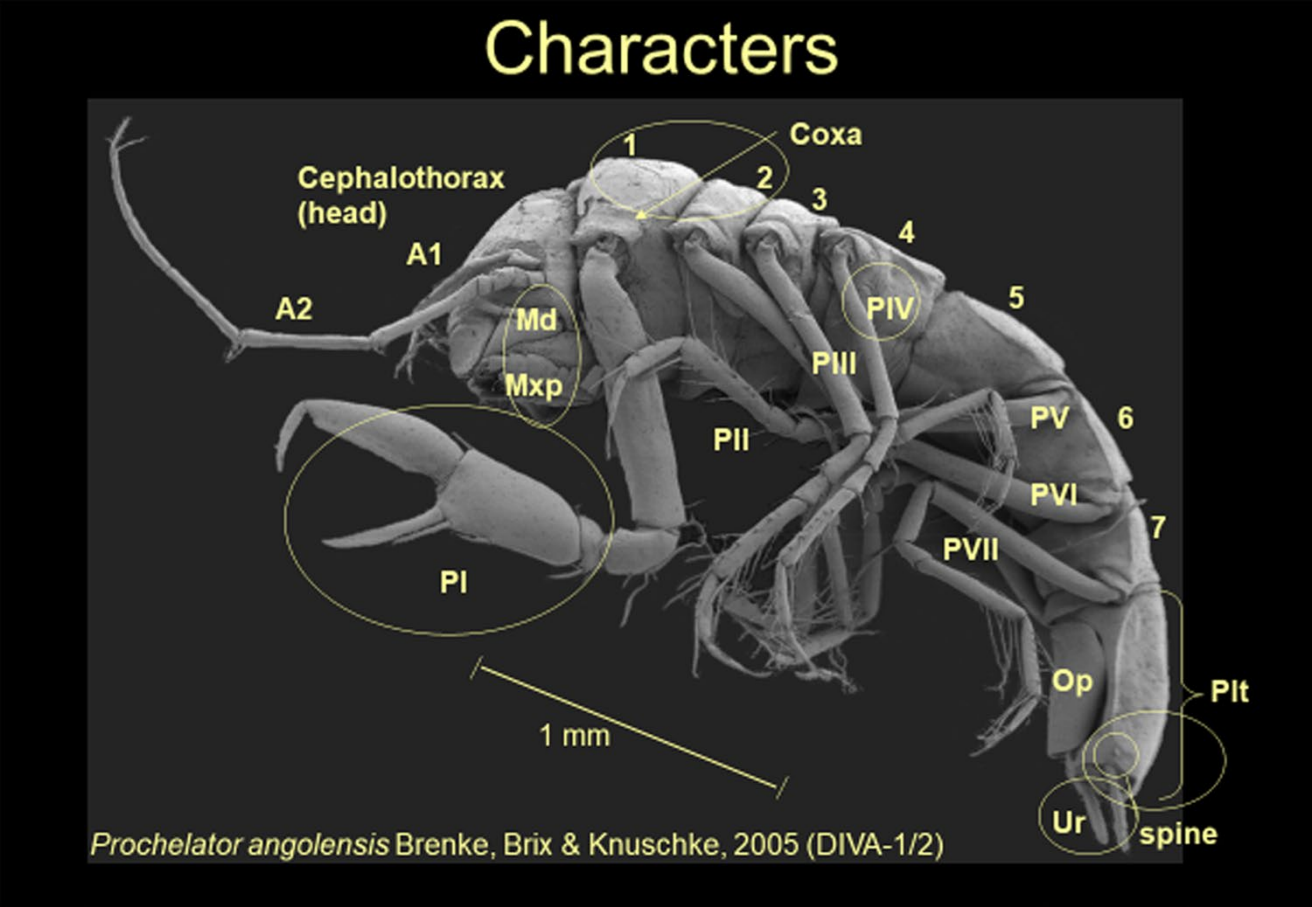
*Prochelator angolensis* Brenke, Brix & Knuschke, 2005 as SEM photo to illustrate a typical desmosomatid habitus. In this species, P I is forming a chelate condition using a large composed seta at the carpus (see Fig. 5J) as counterpart to the propodus. Abbrevations: A1, antennula; A2, antenna; Md, mandible; Mxp, maxilliped; 1–7, pereonites 1 to 7; PI, pereopod I; PII, pereopod II; PIII, pereopod III; PIV, pereopod IV; PV, pereopod V; PVI, pereopod VI; PVII, pereopod VII; Op, operculum; Plt, pleoteson; Ur, uropod; spine, posterolateral spine

**Fig. 5:**
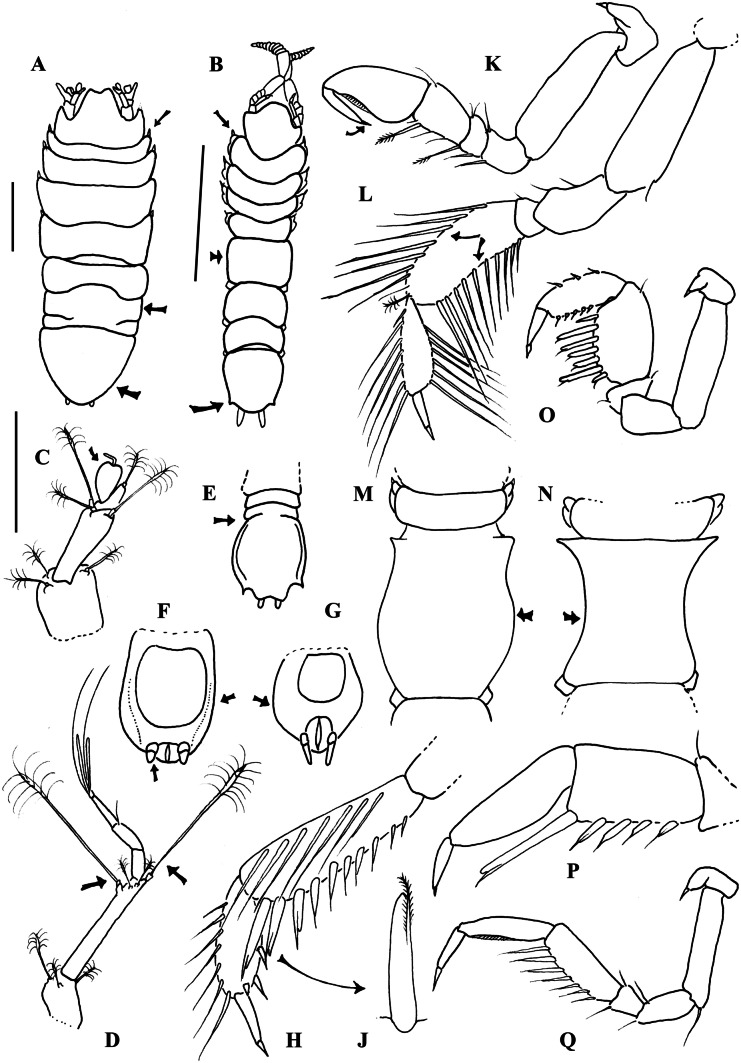
Generalized sketch drawings of main characters discussed in the main manuscript. (**A**) Positioning of setae on either tergite (nannoniscid character, *Nannoniscus oblongus* modified after Wilson, 2008) or (**B**) coxae (desmosomatid character: standardized *Chelator* specimen modified after Brix et al. (2015); (**C**) bulbous 5-segmented antennula modified after Wilson (2008), i.e., *Nannoniscus*, *Rapaniscus*, *Regabellator*, and *Exiliniscus*)); (**D**) antennula article 2 with two large articulated broom setae modified after Hessler (1970); (**E**, **F**, **G**) presence or absence of posterolateral spines at the pleotelson and pleotelson shape as well as degree of somite articulation is variable within nannoniscid genera (e.g., *Nannoniscoides*)—in (**F**) uropods covering anus valves (*Pseudomesus*); (**H**) dorsal row of long setae on carpus of PII (*Echinopleura*) modified after Brix (2007); (**J**) composed (unequally bifid) seta according to Hessler (1970); (**K**) subchelate PI of *Torwolia* after Brix (2007); (**L**) ventral rows of natatory setae at PV-VII in *Eugerda* modified after Park (1999); (**M**) shape of the fifth pereonite comparable to *Torwolia creper* Hessler, 1970 (here: convex); (**N**) shape of the fifth pereonite comparable to *Prochelator hampsoni* Hessler, 1970 (here: concave); (**O**) raptorial and enlarged PI in *Eugerdella* and *Whoia* modified after Hessler (1970); (**P**) chelate PI (*Disparella*); (**Q**) unspecialized PI in *Mirabilicoxa*/*Desmosoma* holding rows of composed setae. Little black arrows may focus the reader’s eye to the illustrated characters

All characters (see Figs. 4, 5) are discussed on the basis of the principles of a phylogenetic analysis sensu Hennig (1966, 1984) and Wägele (2004) implying that the plesiomorphy is relevant for all other taxa (see Electronic Supplements 1, 2, 3). Genera defined by monotypy were included (except for *Chelibranchus* Mezhov, 1986 and *Micromesus* Birstein, 1963) because they support groups of related taxa. For all other genera, a minimum number of two species (type species plus an additional species) were used.

A heuristic search using the software PAUP was conducted with randomized addition of taxa (addseq = random) using tree bisconnection-reconnection (TBR) as swapping algorithm. One thousand replicates were performed (nchuck = 3 chuckscore = 1 nreps = 1000 randomize = trees). Both accelerated transformation (Acctran) and delayed transformation (Deltran) were tested as character state optimisation criteria. Consensus trees were calculated and drawn with TreeView (version 1.6.6, © Roderic D. M. Page, 2001; Page, 1996). Figures were finalized using Photoshop CS5.

## Results

### Species diversity and delimitation

**Fig. 6:**
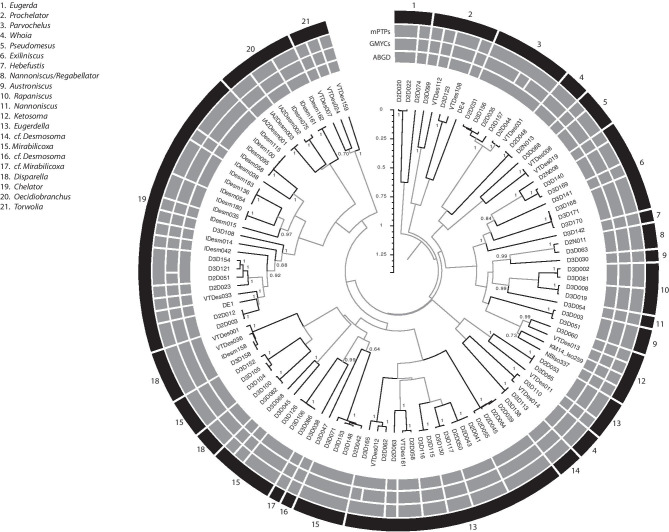
Bayesian, ultrametric, unrooted circle tree for COI. Bayesian posterior probabilities are shown only for nodes relevant to species delimitations (SDs); interior nodes are in gray. Bars in the inner three rings (gray) denote molecular SDs for the three methods as labeled. Bars in the outer black ring denote morphological species determination, with genera indicated in the legend

**Fig. 7:**
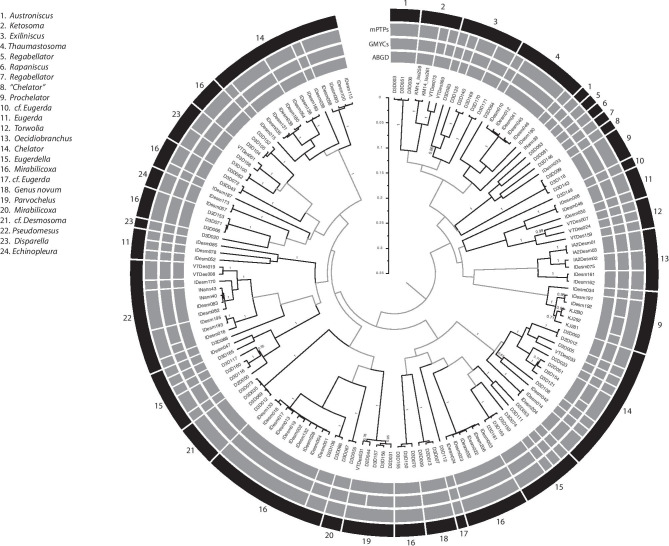
Bayesian, ultrametric, unrooted circle tree for 16S. Format and labeling as in Fig. 4

The three SD methods (ABGD, GMYC, mPTP) produced largely congruent delimitations for both COI (Fig. 6) and 16S (Fig. 7). Out of 121 lineages in COI, ABGD delimited 64 species, GMYC 68, and mPTP 64; out of 155 lineages in 16S ABGD delimited 74 species, GMYC 80, and mPTP 75. These OTUs include 13 valid species names for 16S, nine for COI, and 16 combined; the remaining OTUs were either potentially species new to science or identified to genus level only.

**Fig. 8:**
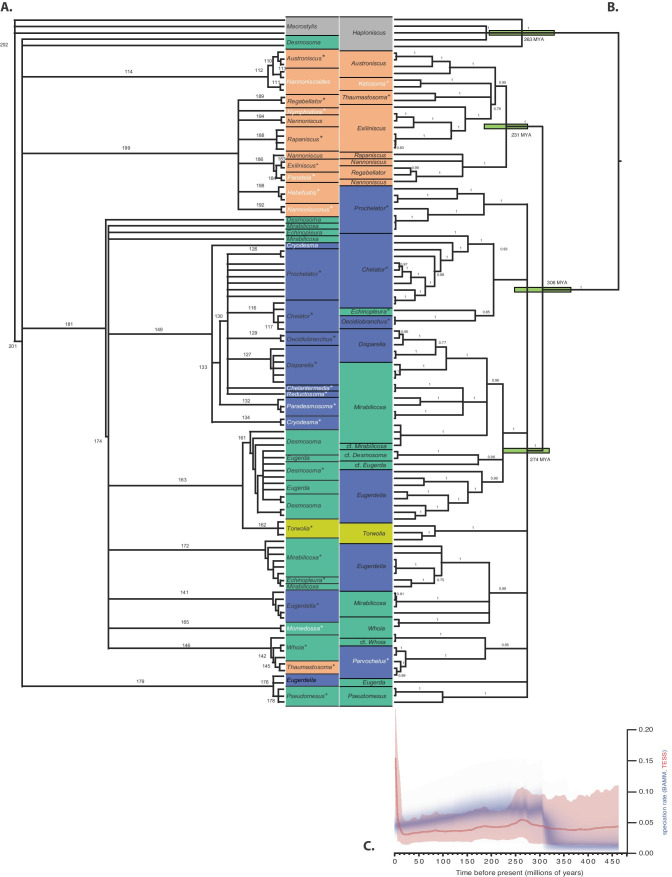
Mirrored morphological and molecular phylogenetic trees. Panel **A**, morphological strict consensus parsimony tree. Numbers on branches indicate steps along that branch. Panel **B**, molecular 2G Bayesian consensus tree. Some nodes were rotated or moved along polytomous bases to maximize vertical correspondence of taxa between the trees. Numbers on branches indicate posterior probability. Green bars show 95% confidence intervals (CI) for estimated divergence dates based on fossil calibrations, using the time scale at figure bottom. In both panels, white text indicates genera found only in that tree and asterisks mark genera for which the type species was included; orange shading denotes the Nannoniscidae, whereas blue (Eugerdellatinae) and green (Desmosomatinae) denote the two subfamilies of Desmosomatidae proposed by Hessler (1970). Panel **C**, estimated speciation rate through time (LTT analysis). The red line and shading show the mean and 95% CI from TESS, and the blue cloud shows the same from BAMM

The morphological dataset contains 107 described species including the type species of all genera except for *Nannoniscus*, whereas the genetic dataset is limited to 74–80 species (see above), most of which are new to science and not yet described by morphological characters. The molecular 2G tree (mirrored to the morphological tree in Fig. 8, with some nodes reordered to maximize vertical correspondence) includes type species of 25 genera (labeled with asterisks). Of the seven type species present in the molecular tree, only *Thaumastosoma platycarpus* Hessler, 1970 and *Pseudomesus brevicornis* Hansen, 1916 are included in both datasets. In the case of *Pseudomesus brevicornis*, sequence data are from the area of the type locality, but not in the case of *Thaumastosoma platycarpus*.

### Morphological and molecular topologies

#### Morphological versus molecular phylogeny

The morphological and molecular 2G trees were largely congruent (Fig. 8). Twelve of the 20 genera present in both trees are monophyletic in both (three nannoniscid and nine desmosomatid genera). The 2G tree recovered Nannoniscidae + Desmosomatidae (the ingroup) as reciprocally monophyletic sister-taxa with high support (0.89–1.00; Fig. 8B). All single-gene trees recovered this ingroup as monophyletic relative to Haploniscidae; however, the monophyly and sister status of Nannoniscidae and Desmosomatidae were only recovered in 18S among single-gene trees (with full support; Electronic Supplement 4). For COI, a monophyletic Nannoniscidae was fully supported but fell among desmosomatid clades (Electronic Supplement 5), and for 16S neither group was monophyletic (Electronic Supplement 6). In both—morphological and molecular analyses—*Pseudomesus* is clearly positioned within Desmosomatidae. Although the morphological data do not resolve at family level, the clade of *Pseudomesus* also contains two *Eugerdella* Kussakin, 1965 species. By contrast, our morphological data suggest *Thaumastosoma* to be the sister clade of *Whoia* Hessler, 1970 within Desmosomatidae, whereas in the molecular data (2G, 18S), the genus is clearly positioned within Nannoniscidae next to *Ketosoma* Kaiser & Brix, 2018 as a sister taxon. It should be noted that the consistency index of all trees found in the morphological phylogenetic analysis is low. Consequently, the homoplasy index is high. The retention index (0.8182) is thought to not be distorted by autapomorphies and symplesiomorphies (Wägele, 2001). This index is distinctly higher than the homoplasy index (0.6815). In total, 49 apomorphies were found only once in the trees, 27 apomorphies twice, while 53 occurred more than twice. Due to these difficulties, morphological tree bootstrap values are not shown.

Within Nannoniscidae, four genera out of the seven present in the 2G tree (Fig. 8) were monophyletic (*Austroniscus* Vanhöffen, 1914, *Exiliniscus* Siebenaller & Hessler, 1981, *Ketosoma*, *Thaumastosoma*); the same number of genera was monophyletic in the 18S, COI, and 16S trees. Within Desmosomatidae, eight genera out of 14 present in the 2G and 18S trees were monophyletic (*Chelator* Hessler, 1970, *Echinopleura* G. O. Sars, 1897 [18S only], cf. *Desmosoma* G. O. Sars, 1864, *Disparella* Hessler, 1970, *Oecidiobranchus* Hessler, 1970, *Parvochelus* Brix & Kihara, 2015, *Prochelator* Hessler, 1970 [2G only], *Pseudomesus*, and *Torwolia*); this number fell to six in COI and 16S. Support of intermediate nodes was generally highest in the 2G and 18S trees, moderate in COI, and low in 16S. No support was recovered in any molecular tree for the two subfamilies defined by Hessler (1970). In both trees—morphological and molecular—*Torwolia* was recovered as *incertae sedis* (Hessler, 1970). Genetic data place the genus in a basal polytomy, whereas the morphological strict consensus shows *Torwolia* as sister clade to a *Desmosoma* + *Eugerda* clade. The 2G, COI, and 16S trees all exhibited topologies with large evolutionary distances between ingroup and outgroup.

### Divergence times, biogeography, and speciation rates

Bayesian estimates of divergence times suggested 263 mya for Haploniscidae, 231 mya for Nannoniscidae, and 306 mya for Desmosomatidae. Both trees tend to have many branches in two “zones” or time periods: near the base of the tree at the nannoniscid/desmosomatid split, and near the tips at the level of genera/species. Both TESS and BAMM detected a significant increase in speciation rate in the older time period, around 270 mya (TESS, Bayes Factor (BF) ≈3) to 330 mya (BAMM, posterior support 0.93–0.95); in BAMM, there was low posterior support of 0.05–0.07 for an increase only in the desmosomatids. TESS also detected a significant, larger increase in speciation rate in the more recent time period, about 10 mya (BF≈12). TESS also detected a significant increase in extinction rate just prior to this period, from roughly 27–10 mya (BAMM does not estimate a separate extinction rate). Supporting these findings, model testing with LTT generated a statistically significant better fit of the birth–death model (speciation + extinction) over the speciation-only Yule model (likelihood ratio test chi^2^ *p* value 0.030), and the Pybus-Harvey gamma statistic was positive (1.7347), indicating that the speciation rate was initially low and subsequently increased (though this statistic was marginally significant at *p* = 0.083).

When collection location (i.e., oceanographic basin) was mapped onto the tips of single-gene trees, no regional patterns were found (Electronic Supplement 7); that is, there was neither convincing evidence of different species being restricted to particular geographic regions, nor of species with broad ranges. Evidence of such patterns was lacking at the generic level as well; in both cases, the datasets suffer from lack of sufficient specimen sampling.

## Discussion

### One or two families?

Our molecular phylogenetic analyses revealed Desmosomatidae and Nannoniscidae to form two well-supported monophyletic clades in the 18S and 2G trees. These datasets represent different inheritance modes and substitution rates, increased by the fact that the faster evolving ribosomal expansion segments in the 18S gene are greatly enlarged in peracarid crustaceans (Raupach et al., 2009). The combination of quickly evolving expansion segments with highly conserved segments likely gave 18S the greatest resolution; conversely, COI and 16S were better resolved at the genus and species level.

The genetic results were not identical to morphological findings, where family-level relationships for Desmosomatidae and Nannoniscidae remained unresolved in a basal polytomy consisting of six major clades (*Desmosoma atypicum* Schiecke & Fresi, 1969, *D. hesslera* Brandt, 1992, *Austroniscus* + *Nannoniscoides*, “Nannonisicdae s.s., Desmosomatidae s.s. and *Pseudomesus* + *Eugerdella*, Fig. 8A). Notably, *Thaumastosoma* spp. is nested within the Desmosomatidae, whereas *Pseudomesus* spp., together with two *Eudergella* species, formed a separate clade distinct from all other desmosomatid and nannoniscid genera. By contrast, molecular analysis clearly assigned *Thaumastosoma* and *Ketosoma* to Nannoniscidae and *Pseudomesus* to Desmosomatidae. One reason for explaining the discrepancy between molecular and morphological topologies might be their different taxonomic scopes: the molecular data contained 21 genera of mostly undescribed species, as opposed to 31 genera, including most of their type species, in the morphological data set. Still, sequences of type species for seven genera were contained in the molecular trees.

Recent phylogenetic work on asellote isopods supports the hypothesis of a rapid and profuse radiation in this group (i.e., a great number of many species generated very quickly), including multiple independent radiations from shallow water into the deep sea (e.g., Osborn et al., 2009; Raupach et al., 2009; Lins et al., 2012; Riehl et al., 2014). In this context, the polytomies and short interior branches recovered in our trees should not only be thought of as a lack of resolution; they also represent the nature of rapid radiation itself, which would make obvious and robust apomorphies difficult to uncover, and would create less genetic differentiation among species than would otherwise be expected. Indeed, LLT analyses (Fig. 8C) provide intriguing evidence for exactly such an increase at the desmosomatid/nannoniscid split, which was likely paralleled in other asellote taxa.

It is known that incomplete taxon sampling can make it difficult to deduce sister relationships. This has more of an influence at higher taxonomic levels than when inferring species relationships (Purvis & Agapow, 2002). A phylogenetic study by Riehl et al. (2014) represents a comprehensive morphological phylogenetic study that includes representative families of the munnopsoid radiation. Using a very reduced taxon sampling for Desmosomatidae and Nannoniscidae respectively, their analyses nevertheless recovered monophyly of both families, while our much more comprehensive morphological data set failed to infer clear phylogenetic relationships. Remarkably, their analysis included the systematically ambiguous genera *Thaumastosoma*/*Ketosoma* and *Pseudomesus*, which they assigned to Nannoniscidae and Desmosomatidae respectively in line with our molecular data (Riehl et al., 2014).

Outgroup choice can have a significant effect on estimated phylogenetic relationships, as demonstrated by Puslednik and Serb (2008). Compounding this difficulty is the munnopsoid radiation itself, which appears to have been rapid and profuse (Lins et al., 2012), nevertheless, despite these authors using different outgroups, Desmosomatidae and Nannoniscidae were consistently recovered as separate, monophyletic taxa.

Based on molecular analysis, both families are clearly monophyletic, when *Pseudomesus* is excluded from the Nannoniscidae, and *Thaumastosoma* and *Ketosoma* are included. Although neither data type should be assumed to be superior to the other (Pisani et al., 2007), diagnostic characters have to be re-evaluated since those currently proposed are not phylogenetically informative. According to Wägele (1989), the following synapomorphies define Nannoniscidae: ventral rows of natatory setae present on pereopods V–VII (Fig. 5L); uropods short covering the anus valves (Fig. 5F/G). In addition, Wilson (2008) reviewed the taxonomic concepts of the Nannoniscidae and pointed out the complexity of characters as discussed in detail further below (see the “Within-family relationships: Nannoniscidae” section).

Desmosomatidae, on the other hand, have been diagnosed as follows: carpus of pereopod I bearing a ventral row of enlarged composed setae (Fig. 5O/P/Q) and a dorsal row of long simple setae; carpus and propodus of pereopod II bearing a ventral row of enlarged composed setae and a dorsal row of long setae (Fig. 5H/J); antennula article 2 with (only) 2 articulated broom setae (Fig. 5D). Although in the molecular analyses, *Thaumastosoma* was placed solidly in Nannoniscidae and *Pseudomesus* solidly in Desmosomatidae, both have ventral rows of natatory setae present on pereopods V–VII (Fig. 5L) as expected for nannoniscids. In addition, species within *Pseudomesus* have short uropods that often cover the anus valves (Fig. 5F), which are considered a nannoniscid synapomorphy. On the other hand, *Rapaniscus* Siebenaller & Hessler, 1981 provides an example of a nannoniscid genus that bears both a ventral row of enlarged compound setae and a dorsal row of long simple setae on the carpus of pereopod I (Fig. 5O), and possessing a ventral row of enlarged composed setae and a dorsal row of long setae on carpus and propodus of pereopod II (Fig. 5H). Also, most genera in both families (only) have two articulated broom setae on article 2 of the antennula (Fig. 5D). Thus, characters diagnosing Nannoniscidae and Desmosomatidae are not truly synapomorphic and should be revised.

### Within-family relationships: Desmosomatidae

A number of diagnostic features have been used to distinguish morphological clades within the Desmosomatidae, including the shape of the first pereopod (Hessler, 1970; Fig. 5K, O, P, Q), the setation of the carpus and propodus of pereopod II (Fig. 5H/J), the shape of the fifth pereonite (Fig. 5A, B, M, N), presence or absence of posterolateral spines at the pleotelson (Fig. 5A, B, E, F, G) as well as pleotelson shape. The position of the genus *Torwolia* Hessler, 1970 was particularly unclear due to the unique subchelate condition of pereopod I (Fig. 5K), which is highly unusual and unique to this family (Hessler, 1970; but see also Brix, 2007).

The subfamilies of Desmosomatidae defined by Hessler (1970) were not supported in either morphological or molecular analysis; indeed, several polytomies within the family prohibited the position of *Torwolia* within Desmosomatidae from being clarified. Our results were similar to Raupach et al. (2009), who could not recover Eugerdellatinae and Desmosomatinae as monophyletic clades, but their analysis placed respective genera in a polytomy. The poor resolution at deep desmosomatid nodes probably reflects the long evolutionary history of the family, and likely indicates rapid evolutionary radiations (Humphries & Winker, 2010; Osborn, 2009). At smaller scales, the monophyly of several genera was similarly rejected by both morphological and molecular analysis (i.e., *Desmosoma*, *Echinopleura*, *Eugerda*, *Eugerdella*, *Mirabilicoxa*, and *Whoia*), whereas others formed well-supported monophyletic clades (*Chelator*, *Disparella*, *Oecidiobranchus*, *Pseudomesus*, and *Torwolia*) (Fig. 8). The position of *Prochelator* in the morphological tree could not be resolved, but its monophyly was suggested by molecular analysis. Unfortunately, only a few sequences could be acquired for *Desmosoma*, and *Echinopleura* which, according to our morphological data, seem to be polyphyletic (Fig. 8). Similarly, for *Cryodesma* Svavarsson, 1988, where the lack of genetic data only allowed morphological assessment, polyphyly of the genus was hypothesized. In this context, greater taxon sampling is desirable in order to test the monophyly of these genera and to clarify their phylogenetic placement.

Within desmosomatids, convergent evolution and analogies could pose a difficulty in defining apomorphies for phylogenetic reconstructions. Here, unraveling of the *Mirabilicoxa* + *Disparella* and *Eugerdella* + *Mirabilicoxa* and *Whoia* clades should currently be one of the main tasks in desmosomatid systematics, since the difficulties of defining different phenotypic clades are symptomatic of the entire family. Morphologically, these genera can be broadly distinguished by the shape of the first pereopod (Fig. 5P: chelate in *Disparella*, Fig. 5O: raptorial and enlarged/robust in *Eugerdella* and *Whoia*, Fig. 5Q: “unspecialized” in *Mirabilicoxa*). However, intermediate character states in some (thus far undescribed) species exist from an unspecialized pereopod I towards a raptorial and chelate condition, making phenotypic assignment based on the first pereopod alone tremendously difficult. *Mirabilicoxa*, in particular, can be viewed as a “grab bag” for species that cannot be assigned to *Desmosoma*, *Eugerda*, *Momedossa* Hessler, 1970 or *Whoia* (first author’s pers. observ.). In the same way, Golovan (2018) states that the definition of *Mirabilicoxa* is still unclear. Many proposed characters were either imprecise or can be also observed in other desmosomatid genera and thus are considered to be plesiomorphic (Brix, 2007). Considering the chelate form of pereopod I, a closer relationship to *Chelator*, *Parvochelus*, and *Prochelator* would have been assumed for *Disparella*, as can be seen in the morphological tree. However, the latter contains a clade possessing a wide range of pereopod I morphologies. Interestingly, a sister-group relationship of *Chelator*, *Parvochelus*, and *Prochelator* could also not be confirmed by the molecular data suggesting that the chelate pereopod I represents an analogous feature. Many *Eugerdella* species have a striking first pereopod, which is characterized by an enlarged propodus and carpus with a ventral row of very robust seta. Overall, however, this genus is very heterogeneous in terms of pereopod I, but also in terms of body shape. Since molecular analyses did not contain any sequences of the type species *Eugerdella coarctata* ( Sars, 1899), it was not possible to designate the true *Eugerdella*. Therefore, further studies are needed to resolve the phylogeny within this genus.

Hessler (1970) hypothesized a “*Eugerdella*-like” condition of the pereopod I (Fig. 5O) in *Whoia* species, which might explain the close linkage of the genus to an *Eugerdella* clade seen both in the morphological and molecular data. However, the morphological resemblance of pereopod I between *Whoia* and *Thaumastosoma* (the latter now confirmed as a nannoniscid, Kaiser et al., 2018) suggests multiple origins of this feature.

Functionally, the first pereopod is used for feeding and grooming (Bauer, 2013; Hessler & Strömberg, 1989). Previous studies conducted on a variety of metazoan taxa have shown that trophic features, including mouthpart and pereopod morphology, can be lost or convergently derived and may therefore not be valuable characters (Apakupakul et al., 1999; Corrigan et al., 2013; Halanych, 1996; Harrington & Reeder, 2017; Havermans et al., 2010; Ruber et al., 1999). Havermans et al. (2010) investigated the phylogenetic relationships within the hyper-diverse superfamily Lysianassoidea and found mismatches between molecular and morphological classification schemes, the latter mainly based on trophic adaptations. However, characters related to dietary habits or grooming do not per se indicate convergent evolution. Bauer (1989), for instance, suggested homology with regard to the location of certain types of pereopod l setae and brushes as phylogenetically informative to derive relationships within Decapoda.

Therefore, just as the first pereopod is not a valuable character for subfamily assignment, it may not even be always useful at the generic level, which means that generic diagnoses need to be thoroughly revised. Here, the subchelate condition of pereopod I in *Torwolia* might be an exception. Our morphological analysis did not provide sufficient resolution, but supported our molecular findings that complex structures such as a chelate (*Chelator*, *Prochelator*, *Parvochelus*, and *Disparella*) or raptorial (*Eugerdella*, *Whoia*) pereopod I can be considered as analogous features that have probably developed several times independently within the family in the course of adaptive processes.

Here, we do not provide a revision for the desmosomatid genera, since certain clades need a thorough revision and moreover type species were not included for all clades in the molecular data, which permitted inference of the respective genera (sensu stricto). Beyond the scope of the present work, but for future steps, a revision of *Mirabilicoxa* s.s., *Eugerdella*, and *Disparella* as well as *Eugerda* and *Desmosoma* will be needed as stated also by Golovan (2015) and Jennings et al. (2020). This includes in case of *Mirabilicoxa* the detection of genetic differences among what have until now been considered different sexes and/or developmental stages leading toward a new understanding of its development and evolution.

### Within-family relationships: Nannoniscidae

Within the Nannoniscidae, different morphological clades have been distinguished, mostly using the antennula (number of articles and specialization of the distal articles, see Fig. 5C) as well as level of articulation of pereonites 6, 7, and/or the pleotelson (Fig. 5A, E) as synapomorphic characters. Accordingly, George (2001) defined three different subfamilies based on the fusion of the posterior somites. This classification, however, was rejected by Wilson (2008). Owing to its anatomical complexity, we expected genera with a bulbous 5-segmented antennula (Fig. 5C: i.e., *Nannoniscus*, *Rapaniscus*, *Regabellator* Siebenaller & Hessler, 1981, and *Exiliniscus* Siebenaller & Hessler, 1981 in our study) to be more derived and separate from genera with an unspecialized antennula (Fig. 5D; Wägele, 1989; and Just, 1970; as detailed below). The molecular data were in support of the hypothesis of *Nannoniscus*, *Rapaniscus*, and *Regabellator* forming a well-supported monophyletic clade both in the 18S and 2G tree. In this regard, the position of *Exiliniscus* appears quite remarkable, forming a group with genera that have an unspecialised antennula, at least at first. The arrangement of the antennula in *Nannoniscus*, *Rapaniscus*, *Regabellator*, and *Exiliniscus* seems to be quite conservative and regarded as homologous feature among respective genera showing a bulbous terminal article and a shelf-like extension of the fourth article (Fig. 2 in Wägele, 1989). While such an extension is present in the type species of *Exiliniscus*, *E. clipeatus* Siebenaller & Hessler, 1981, there is none visible in the remaining described species (Siebenaller & Hessler, 1981, cf. Figure 1 in Just, 1970). In some ways, *Exiliniscus* is quite different from other nannoniscid genera, likely in part reflecting adaptations to a more infaunal lifestyle (e.g., narrow cigar-like body shape, stout first and second antenna, lack of a mandibular palp). Wägele (1989) suggested a close relationship of *Exiliniscus* with *Panetela* and *Micromesus*, which are unfortunately not included in the current analyses. Furthermore, *Hebefustis* Siebenaller & Hessler, 1977 is not included, yet its 5-segmented though unspecialized antennula is thought to represent an intermediate state between the specialized bulbous and unspecialized antennula found in nannoniscids (Siebenaller & Hessler, 1977). At the current stage, our molecular results are more in the line with George’s (2001) classification (taxa with free vs. fused posterior somites), while the antennula is considered as analogous, which has likely developed independently several times. However, we acknowledge that the degree of somite articulation does not display a consistent character and may be variable within nannoniscid genera (e.g., *Nannoniscoides* Hansen, 1916). Therefore, subfamilies introduced by George (2001) are not recovered here. Besides, information from the remaining nannoniscid taxa not included in our molecular analyses will need to be added to draw a “final” conclusion at this stage.

Molecular analyses supported the monophyly of most nannoniscid genera, though *Nannoniscus* was revealed to be polyphyletic in both our morphological and molecular analyses. Siebenaller & Hessler (1981) already highlighted the great morphological variation of *Nannoniscus* species relative to its type species, *N. oblongus* G. O. Sars, 1870, though they did not suggest an alternative classification. So far, *Nannoniscus* is solely defined by plesiomorphies, such as uropods inserting closely to the anus valves (Fig. 5A, F), that define the family Nannoniscidae, or synapomorphies (e.g., bulbous terminal article of the antennula as illustrated in Fig. 5C), characteristic for the respective clade (*Nannoniscus* + *Rapaniscus* + *Regabellator* + *Exiliniscus*). Thus, a thorough morphological and molecular assessment will be required to solve phylogenetic relationships within the clade—also with regard to the variable position of *Regabellator* in the individual 18S vs. 2G tree (Supplement 4 and 6B, respectively).

### Estimation of divergence times/diversification rates

There is now compelling evidence for a long evolutionary history and origination of many asellotan families in the deep sea, well before end-Permian mass extinctions (Jacobs & Lindberg, 1998; Lins et al., 2012; Raupach et al., 2004, 2009; Wilson, 1998). Isopods in general have a long fossil history starting in the Carboniferous period (Wilson, 2009), when malacostracan diversity was bursting on the evolutionary scene (Schram, 1970, 1974). Putative sister groups for the isopods do not appear in the record until later. The oldest Amphipoda seems to be known from the Triassic (200–250 mya, see McMenamin et al., 2013) while a review of the amphipod fossil record is given by Hegna et al. (2019) discussing amphipods first appearing as fossils in the Eocene. Another possible sister group, Tanaidacea, does have one Paleozoic fossil and a more frequent fossil record from the Jurassic on (Schädel et al., 2019; Vonk & Schram, 2007). Lins et al. (2012) confirmed the colonization of the deep sea by isopods on multiple occasions from shallow waters (also Raupach et al., 2004, 2009). This, however, does not apply to the clade of “munnopsoid radiation” (including Nannoniscidae and Desmosomatidae), which likely followed an ancient colonization. In contrast to Lins et al. (2012), our Bayesian estimates of divergence times suggested a younger divergence time for Haploniscidae (263 vs. 310 mya), a younger divergence time for Nannoniscidae (231 vs. 260 mya), and an older divergence time for Desmosomatidae (306 vs. 210 mya), although Bayesian 95% credibility intervals for the first two overlapped the Lins et al. (2012) estimates. Credibility intervals in these analyses are often frustratingly wide, particularly where few molecular markers are employed as is the case here; however, a general consensus is becoming established that the Carboniferous and Permian were especially critical periods in isopod evolution. Consistent with this clustering of divergence times, lineage through time (LTT) analysis strongly suggested (*f* = 0.93–0.95) a rapid increase in speciation rates at the base of the desmosomatid/nannoniscid split, occurring around 300–325 mya at the end of the Carboniferous. During this period, episodic increases in oxygenation (oxygen pulses) might have triggered speciation in many terrestrial and marine groups (Droser et al., 2000; Graham et al., 1995). In contrast, decreasing oxygen, alongside changes in sea level and lower temperature levels during the Permian probably contributed to widespread extinctions and modification of faunal composition (Graham et al., 1995). While Paleozoic and Mesozoic anoxic or dysoxic conditions are believed to have eradicated most of the deep-sea fauna, particularly so in the deep Atlantic and Tethys seas (Jacobs & Lindberg, 1998), other studies suggest that allopatric speciation may even have been promoted by anoxic zones, the latter limiting dispersal between oxygenated patches (Rogers et al., 2000). Another possibility would be that taxa have survived anoxia in shallower refugia on the shelf or slope (Rogers et al., 2000). This scenario seems to be unlikely for Desmosomatidae and Nannoniscidae though, since these families exhibit greatest species diversity in the abyss, and in addition several genera have thus far only been recorded from lower bathyal/abyssal waters (e.g., *Disparella*, *Micromesus*, *Momedossa*, *Thaumastosoma*, *Ketosoma*) suggesting a deep-sea origin. The fact that most of the samples in our data set come from a depth of more than 3000 m hinders the assessment of depth-related patterns, but at the same time underlines the preponderance of Desmosomatidae and Nannoniscidae in the deep sea.

The lack of a phylogeographic signal in our data also supports the assumption of rapid speciation in both families in the world’s oceans, which results in few easily or robustly differentiated morphological features, especially in the Desmosomatidae. Dating the Desmosomatidae/Nannoniscidae split at c. 300–325 mya, both families evolved clearly before the formation of the Atlantic c. 150 mya (Sheridan et al., 1982). Initially consisting of two separate basins, a deep-water connection formed between the North and South Atlantic between 80 and 65 mya, with today’s bathymetric extent and hydrography only becoming established about 10 mya (Schopf, 1980; Priede & Fröse, 2013). Most of the genera analyzed herein seemed to be established toward the end of the Jurassic (ca. 200 mya), which could explain why the groups as a whole are widely distributed across the Atlantic, but no species in our molecular dataset do span large (> 2500 km) geographic ranges (exceptions based on morphology and literature data only may be *Torwolia creper* Hessler, 1970, see Electronic Supplement 8 and *Thaumastosoma platycarpus*, see Electronic Supplement 10). A phylogeographic mapping of oceanic basin of collection onto the COI tree also showed no such correlations (Electronic Supplement 7). Similarly, evidence of a more recent increase in speciation ca. 25–10 mya (Fig. 8C, TESS) corresponds to a late-Oligocene/early-Miocene window associated with increased speciation in, e.g., deep-water corals (Herrera et al., 2012) as Atlantic circulation approached its current configuration. Although these rapid radiations could explain the complicated systematics of desmosomatids and closely related isopod groups, no independent data currently exist with which to evaluate this hypothesis or its implications for the evolution of these taxa.

## Conclusion

Desmosomatidae and Nannoniscidae are distinct isopod families, both of which exhibit substantial convergent evolution, possibly reflecting their ecological diversity as Osborn (2009) has shown for the Munnopsidae. Both the morphological and the fossil-calibrated molecular phylogenies suggest that the high variability of forms and many intermediate character states resulted from a rapid, widespread radiation of species in the deep sea. While it is still difficult to find apomorphies for these groups, in light of their confirmed reciprocal monophyly, taxonomic revision and reexamination of problematic characters are needed to enable better genus diagnoses. Describing more species morphologically may also clarify the relationships indicated by intermediate states. This reanalysis will require a large amount of taxonomic effort (e.g., Brix et al., 2018), but should go far in elucidating the timing, causes, and consequences of rapid speciation in these abundant and ecologically important deep-sea taxa.

## Supplementary Information

Below is the link to the electronic supplementary material.

Supplementary file1 (DOCX 23 KB). Adetailed list of type specimens used is available as Electronic Supplement 1.

Supplementary file2 (DOCX 74 KB). “*a priori*” analysis (i.e. the character discussion, see ElectronicSupplement 2.

Supplementary file3 (DOCX 602 KB). Alist of all characters and their *a priori weighting sensu* Wägele (2004) is presented in Electronic Supplement 3.

Supplementary file4 (PDF 166 KB). Bayesian phylogenetic tree for 18S. Identical taxa have been collapsed with counts in parentheses. Numbers on branches indicate Bayesian posterior probabilities, shown when greater than 0.75.

Supplementary file5 (PDF 162 KB). Bayesian phylogenetic tree for COI. Format and labelling as in Electronic Supplement 4.

Supplementary file6 (PDF 168 KB). Bayesian phylogenetic tree for 16S. Format and labelling as in Electronic Supplement 4.

Supplementary file7 (PDF 175 KB). Phylogenetic tree for COI Bayesian geographical analysis. Tip branch colours indicate oceanic basin where specimens were sampled, and interior indicate colours estimated ancestral location along those branches; see legend at upper left. Taxa have been collapsed where both taxonomy and sampling locality were identical, with counts in parentheses.

Supplementary file8 (PDF 2 MB). Distribution map for the genus *Torwolia* Hessler, 1970 including all species described worldwide.

Supplementary file9 (PDF 2 MB). Distribution map for the genus *Pseudomesus* Hansen, 1916 including all species described worldwide.

Supplementary file10 (PDF 2 MB). Distribution map for the genus *Thaumastosoma* Hessler, 1970 including all species described worldwide.

## Data Availability

All material used in the study is stored in museum collections as indicated in the methods. Type material information, morphological character matrices, and single-gene phylogenetic trees are made directly available as electronic supplements; DNA sequences are deposited in BoLD at https://dx.doi.org/10.5883/DS-DEEPISO and GenBank (see Table 1), and final DNA alignments in DRYAD at https://doi.org/10.5061/dryad.9w0vt4bfp.

## Code availability

N/A.
