# Supplementary material for: Evolution and phylogeny of the deep-sea isopod families Desmosomatidae Sars, 1897 and Nannoniscidae Hansen, 1916 (Isopoda: Asellota)
Source: Org Divers Evol. 2021 Oct 13;21(4):691–717. doi: 10.1007/s13127-021-00509-9 (PMC8510888; doi:10.1007/s13127-021-00509-9)
Supplement: Supplementary file 1 — Supplementary file1 (DOCX 23 KB). Adetailed list of type specimens used is available as Electronic Supplement 1. [file 13127_2021_509_MOESM1_ESM.docx]

**List of type specimens compared**

Type material from different collections was studied. The types of species described in this study from material collected during DIVA-1 and ANDEEP I & II are deposited in the crustacean collection of the Zoological Museum of the University of Hamburg (ZMH K- 40998 to ZMH K-401015).

**University of Hamburg: Zoological Museum**

ZMH K-40998 *Desmosoma renatae* Brix, 2007, holotype female

ZMH K-40999 *Desmosoma renatae* Brix, 2007, allotype male

ZMH K-401000 *Desmosoma renatae* Brix, 2007, paratypes

ZMH K-401001 *Eugerdella theodori* Brix, 2007, holotype female

ZMH K-401002 *Eugerdella theodori* Brix, 2007, allotype male

ZMH K-401003 *Eugerdella theodori* Brix, 2007, paratypes

ZMH K-401004 *Eugerdella serrata* Brix, 2007, holotype female

ZMH K-401005 *Eugerdella serrata* Brix, 2007, allotype male

ZMH K-40106 *Eugerdella serrata* Brix, 2007, paratypes

ZMH K-40674 *Disparella maiuscula* Kaiser & Brix, 2005, holotype female

ZMH K-40676 *Disparella maiuscula* Kaiser & Brix, 2005, allotype male

ZMH K-40675 to K 40682 *Disparella maiuscula* Kaiser & Brix, 2005, paratypes

ZMH K-401007 *Momedossa longipedis* Brix, 2007, holotype female

ZMH K-401008 *Momedossa longipedis* Brix, 2007, allotype male

ZMH K-401009 *Momedossa longipedis* Brix, 2007, paratypes

ZMH K-40331 A – K *Prochelator angolensis* Brenke, Brix und Knuschke, 2005,

holotype female

ZMH K-40322 to K-40323 *Prochelator angolensis* Brenke, Brix und Knuschke, 2005,

paratypes female

ZMH K-401010 *Torwolia tinbinae* Brix, 2007, holotype female

ZMH K-401011 *Torwolia tinbinae* Brix, 2007, paratype female

ZMH K-401012 *Torwolia tinbinae* Brix, 2007, paratype female

ZMH K-40104 *Regabellator abyssi* Brandt, 2002, holotype female

ZMH K-40106 *Saetoniscus meteori* Brandt, 2002, holotype female

ZMH K-40108 *Rapaniscus multisetosus* Brandt, 2002, holotype female

ZMH K-40110 *Nannoniscus antennaspinis* Brandt, 2002, holotype female

ZMH K-40276 *Macrostylis robusta* Brandt, 2004, holotype female

ZMH K-40278 *Macrostylis longipedis* Brandt, 2004, holotype female

ZMH K-40280 *Macrostylis angolensis* Brandt, 2004, holotype female

ZMH K-40282 *Macrostylis meteorae* Brandt, 2004, holotype female

ZMH K-40284 *Macrostylis abyssalis* Brandt, 2004, holotype female

ZMH K-40286 *Macrostylis longspinis* Brandt, 2004, holotype female

**Smithsonian Institution:**

**Natural Museum of Natural History (Washington D.C., U.S.A.)**

USNM 125088 *Balbidocolon atlanticum* Hessler, 1970, holotype female

USNM 125089 *Chelator verecundus* Hessler, 1970, holotype female

USNM 125090 *Chelator vulgaris* Hessler, 1970, holotype female

USNM 125091 *Disparella pachythrix* Hessler, 1970, holotype female

USNM 125092 *Disparella valida* Hessler, 1970, holotype female

USNM 125101 *Mirabilicoxa exopodata* Hessler, 1970, holotype female

USNM 125106 *Momedossa profunda* Hessler, 1970, holotype female

USNM 125107 *Prochelator abyssalis* Hessler, 1970, holotype female

USNM 125108 *Prochelator hampsoni* Hessler, 1970, holotype female

USNM 125109 *Prochelator incomitatus* Hessler, 1970, holotype female

USNM 120963 *Chelator brevicaudus* (Menzies & George, 1972), holotype male^^[[1]](#footnote-1)^*^

USNM 120971 *Disparella neomana* (Menzies & George, 1972), holotype male*

USNM 120972 *Disparella neomana* (Menzies & George, 1972), allotype female*

USNM 120973 *Disparella neomana* (Menzies & George, 1972), 4 paratypes*

USNM 120968 *Disparella funalis* (Menzies & George, 1972), holotype female*

USNM 120969 *Disparella funalis* (Menzies & George, 1972), 3 paratypes female*

USNM 120975 *Eugerdella rotunda* (Menzies & George, 1972), holotype female*

USNM 120966 *Desmosoma dolosa* (Menzies & George, 1972), holotype male

USNM 120967 *Desmosoma dolosa* (Menzies & George, 1972), 1 female, other material

USNM 120962 *Mirabilicoxa acuta* (Menzies & George, 1972), holotype female*

USNM 121711 *Mirabilicoxa similipes* (Menzies & George, 1972), holotype male*

USNM 121712 *Mirabilicoxa similipes* (Menzies & George, 1972), allotype female*

USNM 121750 *Mirabilicoxa similipes* (Menzies & George, 1972), other material*

USNM 171426 *Desmosoma anversense* (Schultz, 1969), holotype sex undetermined

USNM 138732 *Mirabilicoxa hessleri* George 2001, holotype male

USNM 138733 *Mirabilicoxa alberti* (George, 2001), holotype female

USNM 138731 *Prochelator sarsi* (George, 2001), holotype female

USNM 120964 *Rapaniscus coalescum* (Menzies & George, 1972), holotype*

USNM 143607 *Nymphodora fletcheri* (Paul & George, 1975) holotype female

**American Museum of Natural History (New York, U.S.A.)**

AMNH 12112 *Mirabilicoxa birsteini* (Menzies, 1962), holotype*

AMNH 12119 *Mirabilicoxa magnispina* (Menzies, 1962), holotype *

AMNH 12121 *Chelator striatus* (Menzies, 1962), holotype *

-----------------------------------------------------------------------------------------------------------------

Remark: Unfortunately, the type material of these three species is in a very bad condition. Identification of species is not possible even after study of the holotype due to the damage.

-----------------------------------------------------------------------------------------------------------------

**Museum Victoria (Melbourne, Australia**)

J 18597 *Pseudomesus satanus* Kaiser & Brix, 2007, holotype female

J 18608 *Paradesmomsoma australis* Brix, 2006, holotype female

J 18606 *Oecidiobranchus slopei* Brix, 2006, holotype male

J 18605 *Disparella kensleyi* Brix, 2006, holotype female

J 18600 *Echinopleura cephalomagna* Brix, 2006, holotype female

J 18601 *Echinopleura cephalomagna* Brix, 2006, paratype female

J 53074 *Echinopleura cephalomagna* Brix, 2006, paratype female

J 18598 *Whoia victoriensis* Brix, 2006, holotype female

J 18599 *Whoia victoriensis* Brix, 2006, paratype female

J 18612 *Chelantermedia composita* Brix, 2006, holotype female

**Australian Museum (Sydney, Australia)**

-----------------------------------------------------------------------------------------------------------------

Remark: The paratypes and allotypes of species which Hessler (1970) described in his monograph are deposited in the Australian Museum in Sydney.

-----------------------------------------------------------------------------------------------------------------

AM P59160 *Chelator vulgaris* Hessler, 1970, paratype female

AM P58856 *Chelator insignis* Hessler, 1970, paratype female

AM P59082 *Prochelator litus* Hessler, 1970, paratype female

AM P59075 *Prochelator abyssalis* Hessler, 1970, paratype female

AM P59197 *Prochelator hampsoni* Hessler, 970, paratype female

AM P58781 *Prochelator lateralis* (Sars, 1897)

**New Zealand Institution of Oceanographic and Atmospheric Research (NIWA) (Wellington, New Zealand)**

“Lincoln material”

NIWA 33747 *Prochelator tupuhi* Brix & Bruce, 2008, holotype

NIWA 33749  *Prochelator tupuhi* Brix & Bruce, 2008, paratype

NIWA 33750 *Prochelator tupuhi* Brix & Bruce, 2008, paratype

**Zoologisk Museum** [København](https://onlinelibrary.wiley.com/doi/abs/10.1002/mmnd.4800330318)

**(Kopenhagen, Danmark)**

ZMUC CRU-510 *Chelator chelatum* (Stephensen, 1915), holotype* female

ZMUC CRU plus 14 specimens deposited as „other material” (nontype Isopoda, blue label, no number) det. E. Fresi as *Desmosoma chelatum*, Ischia, Italy, 110 m, 16 May 1968

ZMUC CRU *Prochelator serratum* (Fresi & Schiecke, 1969)*, nontype Isopoda (blue label, no number), det. E. Fresi, Ischia N., Italy 80-110 m, May 1968

ZMUC CRU-7027 *Prochelator lateralis* (Sars, 1897)*, types

ZMUC CRU-514 *Eugerda globiceps* Meinert, 1890, types

ZMUC CRU-515 *Eugerda globiceps* Meinert, 1890, types

ZMUC CRU-516 *Eugerda globiceps* Meinert, 1890, types

ZMUC CRU-517 *Eugerda globiceps* Meinert, 1890, types

ZMUC CRU-518 *Eugerda globiceps* Meinert, 1890, types

ZMUC CRU-9206 *Eugerda globiceps* Meinert, 1890, types

ZMUC CRU-588 *Chelator insignis* (Hansen, 1916)*, lectotype

ZMUC CRU-589 *Chelator insignis* (Hansen, 1916)*, paralectotype

ZMUC CRU-7500 *Eugerdella natator* (Hansen, 1916)*, holotype

ZMUC CRU-7810 *Oecidiobranchus plebejum* (Hansen, 1916)*, lectotype

ZMUC CRU-7828 *Eugerdella polita* (Hansen, 1916)*, syntype

ZMUC CRU-9170 *Mirabilicoxa similis* (Hansen, 1916)*, lectotype

1. * Types are deposited as *Desmosoma*. [↑](#footnote-ref-1)
