# Supplementary material for: Evolution and phylogeny of the deep-sea isopod families Desmosomatidae Sars, 1897 and Nannoniscidae Hansen, 1916 (Isopoda: Asellota)
Source: Org Divers Evol. 2021 Oct 13;21(4):691–717. doi: 10.1007/s13127-021-00509-9 (PMC8510888; doi:10.1007/s13127-021-00509-9)
Supplement: Supplementary file 2 — Supplementary file2 (DOCX 74 KB). “a priori” analysis (i.e. the character discussion, see ElectronicSupplement 2. [file 13127_2021_509_MOESM2_ESM.docx]

**Discussion of characters used in the morphological phylogeny (Figure 6A)**

The DELTA matrix concentrates on highly complex characters, which are hypothesized to be phylogenetically informative. Macrostylidae are used as the outgroup (Macrostylidae are closely related representatives of Janiroidea). The outgroup is defined by 12 characters (8, 14, 20, 21, 42, 65, 68, 69, 71, 118, 124, 125). Of these characters, only the uniramous uropod (124) and the loss of the mandibular palp (42) occur also in Desmosomatidae and Nannoniscidae. All other characters are apomorphic in Desmosomatidae and Nannoniscidae are plesiomorphic in Macrostylidae.

Characters of Nannoniscidae and Desmosomatidae are treated equally. Both families are analysed as one group. All characters are discussed on the background of the principles of a phylogenetic analysis *sensu* Hennig (1966, 1984) and Wägele (2000, 2004). Plesiomorphic character states are shown in brackets behind the autapomorphic state. This implies that the plesiomorphy is relevant for all other taxa. Characters of sexual dimorphism are not used within the phylogenetic analysis because males and females are not known for all species. For the phylogenic analysis, only species with a detailed description of adult or preparatory females are used or, alternatively, material that could be borrowed from museum collections.

Genera defined by monotypy are included (except for *Chelibranchus* and *Micromesus*) because they support groups of related taxa. For all other genera, a minimum number of two species (type species plus an additional species) is used for the phylogenetic analysis.

**Setation**

Hessler (1970) described the setation as conservative. Within a series of setae, such as the ventral row on the carpus, the range of variation generally does not exceed one or two setae (Hessler 1970). The data he gathered for his comparison of individuals showed that in *Desmosoma tetarta* (Hessler, 1970), the range of variation is low, whereas it is high in *Whoia variabilis* Hessler, 1970. Further, a species may show a low standard variation at one locality, but it may still vary from place to place (Hessler 1970). There are clear differences in the number of setae within the setal rows on the carpus and the propodus between species. Therefore, utilisation of the setal number for phylogenetic analysis becomes critical. One reason not to use numbers of setae is the necessity to determine the intraspecific variation for each species used for the phylogenetic analysis. In many cases the available number of individuals of a species is too low for statistical comparison. Additionally, setal numbers may vary between different developmental stages as seen in *Desmosoma tetarta* (Hessler, 1970). This does not affect the phylogenetic analysis presented in this thesis because only adult or preparatory females are used. However, in order to avoid numerical mistakes, this character matrix concentrates on the presence of setal types, not on numbers of setae.

Hessler (1970) concluded that the presence of many kinds of setae is highly conservative such that setae fulfil a specific sensory function. Without doubt, every seta fulfils a certain function. Nevertheless, the question is if setae at specific positions can be homologised *a priori*. Some setae are not only constant in numbers in a species but are present in all members of the family at the same position (an example is article 2 of the first antenna carrying 3-4 distomedial broom setae). Hessler (1970) hypothesized that such characters, although minute, may serve for the family diagnosis unless they are constant in other asellote families as well. Hessler´s hypothesis about the broom setae was used by Wägele (1989) when he defined “*two big broom setae inserted opposite to each other”* as an autapomorphy of Desmosomatidae.

**Characters**

In the following, a discussion of each single character used for the phylogenetic analysis is presented. The characters discussed here are the same as defined in the DELTA-matrix (ES1). They are numbered the same way. An **O** marks a character that is defined as “ordered” and the direction of evolution is defined before the computer analysis using PAUP; a **U** marks a character that is defined as “unordered”. The taxa for which the synapomorphies, autapomorphies and plesiomorphies are defined are shown in brackets behind the plesiomorphy.

## Body

## 1) O Whole body serrated. [Not the whole body serrated.] (*Echinopleura* species and *Prochelator serratum*)

Not only the two *Echinopleura* species have a completely serrated body. *Prochelator serratum* also possesses serrated body segments and a serrated pleotelson. *Eugerdella serrata* and *Prochelator serratum* show most probably convergent evolution. Nevertheless, the character confirms the relationship of the *Echinopleura* species.

## 2) O Only margins of pereonite 5-7 and pleotelson strongly serrated. [Not only margins of pereonite 5-7 and pleotelson strongly serrated.] (*Eugerdella serrata* and *E. pugilator*)

Serration, including body segments 5 to 7 and the pleotelson, is found in both species *Eugerdella serrata* sp. nov. and *E. pugilator*. This character is limited to the posterior part of the body and due to the large differences found in *Eugerdella* species convergent evolution is hypothesized. Therefore, serration of the posterior body part is defined as apomorphy.

**3) O Pereonites and pleotelson laterally expanding into flat marginal extensions. [Pereonites and pleotelson not with flat lateral extensions.]** (“nannoniscid character”: *Austroniscus*, *Nannoniscoides* and some species of *Nannoniscus^[[1]](#footnote-1)^*)

This character was presented by Siebenhaller & Hessler (1981) to distinguish *Thaumastosoma* from other nannoniscid genera. In Nannoniscidae, *Austroniscus* species in particular have a depressed body with an oval shape. The pereonites and pleotelson expand laterally into flat marginal flanges, doubling the real width of the body and extending posteriorly. This condition must have evolved secondarily, because the flattened shape of the body of other Isopoda includes whole body segments, not only lateral extensions. The lateral extensions are regarded as an autapomorphy of *Austroniscus* by Siebenhaller & Hessler (1981).

**4) O Body broad (between 2 times and 3.5 times longer than tergal width of pereonite 2). [Body slender (between 3.5 and 5 times longer than tergal width of pereonite 2).]** (*Austroniscus*, *Nannoniscoides* and species of *Nannoniscus*)

According to Wägele (1989), nannoniscids have a broader body than desmosomatids. This is true for the genera *Austroniscus*, *Nannoniscoides* and for some species of *Nannoniscus*. Species of other nannoniscid genera are very similar to the body shape found in desmosomatids: slender and a body length more than three times the width of pereonite 2. Species of some genera that were assigned to Nannoniscidae are of a cigar-like body shape (*Panetela*, *Exiliniscus*). According to Wägele (1989), this may be regarded as secondarily evolved. The elongated body shape of *Pseudomesus* may also be secondarily evolved like in some other species of Desmosomatidae (*Eugergella ischnomesoides*, *E. hessleri*).

The wide range of body shapes makes it difficult to present possibly homologous characters. The slender body shape is regarded as plesiomorphy while a depressed body is hypothesized to be an apomorphy. The broad body shape of *Austroniscus* results from a secondarily evolved lateral extension of the tergits, not from whole body segments.

**5) U Body anteriorly wide and posteriorly slender. [Body not anteriorly wide and posteriorly slender.]** (“desmosomatid character”)

Originally, this character was presented in the generic diagnosis of *Balbidocolon* (Hessler 1970). Later this body shape was discussed as typical desmosomatid body shape (Siebenhaller & Hessler 1977, 1981; Svavarsson 1984). Thus, it is defined as an apomorphy for species belonging to Desmosomatidae.

**6) O Body elongated (more than 5 times longer than tergal width of pereonite 2). [Body slender, but not elongated (between 3.5 and 5 times longer than tergal width of pereonite 2).]** (species of *Eugerdella* and *Pseudomesus*)

In Desmosomatidae a body more than five times longer than the tergal width of pereonite 2 occurs in *Eugerdella hessleri* Just, 1980 (6.8 times the tergal width of pereonite 2) and *E. ischnomesoides* Hessler, 1970 (6.1 times the tergal width of pereonite 2). These two species also bear an elongated segment 5 with convex margins. The elongation of pereonite 5 is the main difference in body shape to distinguish the two *Eugerdella* species from species of *Pseudomesus*. All species of *Pseudomesus* are about six times longer than wide (tergal width of pereonite 2). Therefore, the elongate body shape is discussed as a phylogenetically useful character since the similarity in the body shape correlates with similarities in the characters of pereopod I that are considered to be highly phylogenetically informative.

**7) O Body cigar-like with straight body margins (all pereonites of similar width). [Body not cigar-like with straight body margins.]** (*Exiliniscus*, *Panetela*)

“Cigar-like and with straight body margins” is the characteristic body shape for the nannoniscid genera *Exiliniscus* and *Panetela*. In *E. clipeatus* Siebenhaller & Hessler, 1981 and *E. aculeatus* Siebenhaller & Hessler, 1981, pereonites 3 and 4 are of similar size and shape as pereonites 5 and 6. Pereonite 1 is smallest. In *Panetela wolffi* Siebenhaller & Hessler, 1981, pereonites 4 and 5 are the longest and 1 and 2 are nearly the same size and the smallest. This body shape is regarded as apomorphy for the species of these two genera.

**8) O Close grouping of pereonites 1-3. [Pereonites 1-3 clearly separated by intersegmental skin, not closely grouped]** (“macrostylid character”)

Macrostylidae are easily distinguished from Nannoniscidae and Desmosomatidae by their distinctive body shape as diagnostic character. The terminus “distinctive” is presented as a distinguishing feature from desmosomatids and nannoniscids by Siebenhaller & Hessler 1981. One feature to clarify “distinctive” is the close grouping of pereonites 1-3, while the first three pereonites are usually clearly separated by intersegmental gaps. The close grouping of the first three pereonites occurs in all Macrostylidae. Therefore, it is hypothesized to be homologous within this family and considered to be an apomorphy of Macrostylidae.

**9) O Pereonite 1 not broad and half of size of pereonite 2 or smaller. [Pereonite 1 not broad nor half of size of pereonite 2 or smaller.]** (*Desmosoma* species, *Echinopleura cephalomagna*, *Eugerda* species, *Torwolia* species)

This character is typical for desmosomatids that have a specialized slender pereopod I e.g. *Torwolia* species, *Desmosoma* species and *Eugerda* species as well as *Echinopleura cephalomagna*. Thus, the character is defined as an apomorphy while a pereonite similar in size to the following pereonites is hypothesized as plesiomorphic condition.

**10) O Pereonite 1 broad and clearly smaller than pereonite 2. [Pereonite 1 not broad and smaller than pereonite 2.]** (*Austroniscus*, *Nannoniscoides* species, some *Nannoniscus* species)

In Nannoniscidae, a group of species have a small pereonite 1. In Nannoniscidae, the small pereonite 1 correlates with a broad body shape while in Desmosomatidae, there seems to be a correlation to the specialization of pereopod I. In Nannoniscidae, pereopod I is not specialized in species which have this character. Thus, convergent evolution is hypothesized in this case. A small pereonite 1 is thought to be a separate apomorphy for this group of nannoniscids.

**11) O Pereonite 2 largest of pereonites 1-4. [Pereonites 1-4 subequal.]** (*Torwolia*, species of *Eugerda*, *Desmosoma hesslera*)

The enlargement of pereonite 2 reflects the enlarged and robust pereopod 2 in the genera *Torwolia* and *Eugerda* as well as in *Desmosoma hesslera*. Characters 11 and 12 are often combined in species of both genera. Although, not all species that have one character also have the other. Both characters are regarded to be apomorphic.

**12) U Pereonites 1-4 higher than pereonites 5-7. [Pereonites 1-4 of same height as pereonites 5-7.]** (“desmosomatid character”)

The typical body shape of a desmosomatid in lateral view is flattening from the cephalothorax/pereonite 1 towards the posterior pereonites. The anterior pereonites are always higher than the posterior ones. The same condition is found within some groups of Nannoniscidae (for example in species of *Regabellator*, *Rapaniscus*, *Thaumastosoma* or *Pseudomesus*), but not within Macrostylidae. In Munnopsididae the body shape is highly variable. Within the janiroid groundpattern, all body segments are hypothesized to be of the same height. Therefore, this character state is used as apomorphy.

**13) O Pereonites 5-7 enlarged. [Pereonites 5-7 not enlarged.]** (species of *Desmosoma*, *Eugerda*, *Torwolia*)

The enlargement of the posterior pereopods is probably the result of the swimming life-style of those species. The posterior pereonites hold strong muscles for these enlarged limbs that carry rows of long natatory setae on carpi and propodi. The enlargement of the limbs is correlated with this character, but not necessarily. Thus, the enlargement of the posterior pereonites is treated as a separate character and defined as an apomorphy.

**14) Pereonites 1-4 shorter than pereonites 5-7 [Only anterior 3 pereonites shorter than pereonites 4-7.]** (“macrostylid character”)

Originally, pereonites 1-3 were shorter than the following pereonites (as in Macrostylidae, compare chapters 3.1.2; 4.1.1; 4.1.2). The adaptation of the fourth pereonite, which more closely resembles one of pereonites 1-3 in Desmosomatidae and Nannoniscidae, is regarded as apomorphy.

**15) O Pereonite 5 with clearly convex lateral margins. [Lateral margins of pereonite 5 not convex.]** (*Pseudomesus atypicum*, *Eugerdella hessleri*, *Panetela wolffi*)

(see character 17)

**16) O Pereonite 5 inflated. [Pereonite 5 not inflated.]** (*Torwolia*, *Eugerdella hessleri*)

(see character 17)

**17) O Pereonite 5 elongated. [Pereonite 5 similar in size to pereonite 6.]** (*Pseugerdella*, *Pseudomesus pitombo, Whoia species*, *Momedossa* species)

Character 15 distinguishes *Eugerdella hessleri* from species of the genus *Pseudomesus.* In *Pseudomesus* pereonite 5 is elongated, but the lateral margins are not convex. Inflation also occurs in species of *Torwolia*, but is not correlated with strongly convex lateral margins. Elongation seems to have evolved more than once in Desmosomatidae and occurs also in *Momedossa* and *Whoia*. Hence, elongation of pereonite 5 (17), an inflation (16) and strongly convex lateral margins (15) are regarded as apomorphies.

**18) O Pleotelson enlarged. [Pleotelson not enlarged.]** (*Pseudomesus* species, *Nannonisconus* species, *Eugerdella*)

Enlargement of the pleotelson is defined as an apomorphy regardless of the shape of the pleotelson.

**19) U Body highly vaulted in transverse section, especially in pleotelson. [Body in transverse section axis not highly vaulted, lateral fields presenting a continuous profile.]** (*Chelator, Oecidiobranchus, Reductosoma*)

The plesiomorphic condition is found for example in species of *Mirabilicoxa* and is presented as a character in the diagnosis of this genus (Hessler 1970). Highly vaulted bodies are found in *Chelator,* *Oecidiobranchus* and *Reductosoma*. This may indicate convergent evolution. For species of *Chelator* and *Oecidiobranchus,* a close relationship is hypothesized due to the characters of pereopod I. Therefore, this character might be phylogenetically informative within a monophyletic group of species with a chelate pereopod I.

**20) O Pereonites 4-7 posteriorly acute. [Pereonites 4-7 not posteriorly acute.]** (“macrostylid character”)

In Macrostylidae, the first three pereonites are closely grouped and clearly differ from pereonites 4-7. Additionally, these pereonites are posteriorly acute. This condition is not found in Desmosomatidae and Nannoniscidae and may be regarded as an autapomorphy of Macrostylidae.

**21) O Posterior angles of the tergits of pereonites 4-7 with one stout spine. [Posterior angles of the tergits of pereonites 4-7 without a stout spine.]** (“macrostylid character”)

Pereonites 4-7 of Macrostylidae are acute and the posterior angles of the tergits are tipped with a stout spine. This condition is neither found in Desmosomatidae nor in Nannoniscidae and may be regarded as an autapomorphy of Macrostylidae.

## Cephalothorax

## 22) O Cephalic keels between antennular folds. [Cephalic keels between antennular folds absent.] (“nannoniscid character”: *Austroniscus*, *Nannoniscoides*, *Nannonisconus*, *Nannoniscus*)

These bilateral rostral-like structures are very similar in species of nannoniscid genera with a broad body and do not occur in nannoniscids with a slender body shape. Thus, this character is regarded as an apomorphy of this group of taxa.

**23) O Cephalon with rostrum. [Cephalon without rostrum.]** (autapomophy *Exiliniscus*)

The janiroidean groundpattern does not include a rostrum. Species of the nannoniscid genus *Exiliniscus* bear a rostrum between the insertion of the antennae. Therefore, the presence of a rostrum is regarded as an autapomorphy of this genus (Siebenhaller & Hessler 1981). It is hypothesized that other generic characters also confirm the monophyly of the genus such as a robust antenna, loss of the mandibular palp (although discussed as weak character), and a uniform body shape.

No desmosomatid species bears a rostrum. Likewise, in Macrostylidae, no rostrum is known. There are desmosomatid species such as *Eugerdella pugilator* Hessler, 1970 and *E. serrata* sp. nov. which possess cephalic spines that resemble a rostral structure. However, these rows of spines must have evolved independently due to their position at the dorsal margin of the antennular fold where the antennulae and antennae are inserted (character 24).

**24) O Dorsal margin of antennular fold with row of spines resembling a rostral structure. [Dorsal margin of antennular fold without any spines resembling a rostral structure.]** (*Eugerdella pugilator, E. serrata*)

This spine row on the cephalon is found in *Eugerdella pugilator* Hessler, 1970 and *E. serrata* sp. nov. but in no other desmosomatid species. With regard to the high similarity of characters of pereopod I (character 81), these two species may form a group within *Eugerdella*. This relationship is confirmed by the presence of the spine row.

**25) O Margin of antennular fold with one anteriorly directed spine. [Margin of antennular fold without distinct spine.]** (*Disparella, Mirabilicoxa cornuta* and *M. acuminata, Hebefustis, Nannoniscoides, Prochelator lateralis* and *P. uncatus*)

A pronounced anteriorly directed spine on the antennular fold occurs in species of different genera in Desmosomatidae, especially in *Mirabilicoxa cornuta* (Hessler, 1970), *Prochelator* and all species of *Disparella*. For *Disparella*, such a projection (cephalic spine) is presented in the generic diagnosis (Hessler 1970). The character appears to be weak, but is used in the phylogenetic analysis due to its possible value in characterizing a subgroup (*Disparella*) within a group of species that were previously defined by highly weighted characters like the chela of pereopod I. As such a cephalic spine also occurs in Nannoniscidae, it may have evolved convergently, but seems to be useful in defining small groups of taxa.

## Antennula

## 26) O Antennula consisting of 5 articles. [Antennula consisting of 6 or more articles.]

Only in the genera *Austroniscus* and *Nannoniscoides*, some species are included that possess an antennula with 7 articles. The reduction to five articles instead of six or more is regarded as an apomorphic condition. The possibility of convergent reduction restricts the phylogenetic use of this character. Although it appears to be weak, it may be useful in a group that is clearly defined as a monophylum when the ventral number of antennular articles may be used as apomorphic character state. The number of articles becomes important with regard to the evolution of the specialized antennulae with a bulbous last article (discussed below).

**27) O Antennula with *specialized* distal articles. [Antennula with *unspecialized* distal articles*.*]** (“nannoniscid character”)

The specialized antennula includes more than one character. The specialization may include only the last article which is bulbous (*Hebefustis* and *Nannoniscoides*) or involve also an elongation of the previous article holding the last bulbous one. Character 27 is used to distinguish between a last bulbous article only (characters 28 and 29) or a character complex that depends on patterns of the previous articles (characters 30 to 32). The specialization of the antennula as defined in characters 30 to 32 is hypothesized to have evolved only once. Therefore, defining a large group of species, the “true Nannoniscidae”, while a bulbous last article alone may have evolved twice as defined in characters 28 and 29.

Whether the reduction of the flagellar articles from 4 to 3 proceeds the specialization of the distal articles is crucial in understanding the evolution of the distal antennula. If the reduction to three articles is followed by this specialization, this evolution needs a minimum of two steps and must be the apomorphy while the bulbous last article as the fourth flagellar article evolved convergently.

In some species of *Nannoniscus*, the elongation holding the last bulbous article is located at the first flagellar segment according to drawings of Menzies & George (1972). For example, in *Nannoniscus ovatus* and *Nannoniscus muscarius* there is no true elongation, and in *Nannoniscus perunis* there are two. Possibly there are mistakes in the drawings. The type material was not studied to clarify the questionable descriptions. However, the monophyly of *Nannoniscus* is questioned, as only plesiomorphies define the genus and species of this genus show a high variability.

**28) O Flagellum with rounded bulbous last article. [Flagellum not with rounded bulbous last article.]** (“nannoniscid character” occurring in species of *Exiliniscus*, *Nannonisconus*, *Nannoniscus*, *Nymphodora fletcheri*, *Panetela*, *Rapaniscus*, *Regabellator*, *Saetoniscus* and *Hebefustis*)

Although the bulbous terminal article of the antennula seems to be a conservative condition, there are exceptions in the features of this character. The bulbous article is only one of the characters regarded as phylogenetically informative (character 27). It is hypothesized that a bulbous last article evolved from a fusion of distal articles or an inflation of the distal article (except for the condition found in *Nannoniscoides*). This probably was followed by an elongation of flagellar article 2 which holds the third bulbous article and the bulbous form became more pronounced. The flagellar article 1 was reduced with further functional specialization of flagellar articles 2 and 3. Species of the genus *Hebefustis* bear a bulbous terminal article, but no elongation at the flagellar article 2. This is considered as a plesiomorphic condition within the evolutionary steps necessary for the specialized antennula.

**29) O** **Antennula with bulbous and long terminal article (clearly longer than wide). [Terminal article of antennula not bulbous and long.]** (*Nannoniscoides*)

(see character 27 and 28)

**30) O Flagellar article 1 of antennula smallest. [Flagellar article 1 of antennula not smallest.]** (“nannoniscid character” occurring in: *Exiliniscus*, *Nannonisconus*, *Nannoniscus*, *Panetela*, *Rapaniscus*, *Regabellator*, *Saetoniscus*)

The features of the flagellar articles have to be regarded as a complex character. It is difficult to decide if the features of the antennulae with a terminal bulbous flagellar article are homologous or convergently evolved^[[2]](#footnote-2)^.

**31) O Flagellar article 2 of antennula with elongation holding terminal bulbous article. [Flagellar article 2 of antennula without elongation holding terminal bulbous article.]**

(see character 27 and 28)

**32) O Terminal article of antennula bulbous and ball-shaped. [Terminal article of antennula not bulbous and ball-shaped.]**

(see character 27 and 28)

**33) O Article 2 of antennula elongated (twice as long as first peduncular article). [Article 1 and 2 of antennula of the same size.]** (character 13)

This character state was used as a synapomorphy for the sistergroups Desmosomatidae and Nannoniscidae by Wägele (1989). In members of both families, the second peduncular article is the longest article of the antennula. In Macrostylidae, the first peduncular article of the antennula is the largest. It is used in the phylogenetic analysis to separate Macrostylidae from the sistergroups Desmosomatidae and Nannoniscidae.

**34) O Article 2 of antennula distally with 3-4 joint articulated broom setae. [Broom setae sporadically present.]** (character 14)

In Macrostylidae, broom setae are sporadically present on the second peduncular article while in Nannoniscidae and Desmosomatidae the number of joint articulated broom setae is constant. The constant presence of broom setae is not known in the closest related group, Munnopsididae. Therefore, the constant number of three or four articulated broom setae is regarded as an apomorphy according to Wägele (1989).

**35) O Article 2 of antennula distally with (just) two joint articulated broom setae. [Article 2 of antennula with more than two joint articulated broom setae at distal end.]** (character 20)

This character is presented as an autapomorphy for Desmosomatidae by Wägele (1989). A question to be raised with regard to the original definition of this character is: what kind of broom setae may be included in the definition. In the present analysis, the character means joint articulated broom setae only. Broom setae with a simple cuticular articulation are not included.

#### Antenna

**36) O Shortened and robust antenna, reaching only one quarter of the body length. [Antenna long and slender, clearly longer than one quarter of the body length.]** (*Exiliniscus*)

This character state is presented in the generic diagnosis of *Exiliniscus* and is regarded as an autapomorphy of this genus (Siebenhaller & Hessler 1981).

## Mandible

### 37) O Lacinia mobilis reduced to one small tooth. [Lacinia mobilis with 3 to 5 teeth.] (*Echinopleura, Whoia victoriensis*)

Both species of *Echinopleura* possess a lacinia mobilis that is only one small tooth. This character state occurs also in *Whoia victoriensis*. It is regarded as an apomorphy of *Echinopleura*.

**38) O Incisior process bent forward as one strong tooth.** **[Incisior process not bent forward as one strong tooth.]** (*Thaumastosoma*)

This character is one of the features of the mandible that is influenced by the anteriorly directed production of the mouthparts in *Thaumastosoma*. Thus, it is regarded as an autapomorphy of the genus. The incisior process is also bent forward in *Cyodesma agnari* but not so strongly and with two distal teeth.

**39) O Incisior process without teeth. [Incisior process with teeth.]** (*Echinopleura*)

Simplified mandibles are presented as a character in the generic diagnosis of *Echinopleura* (Sars 1864, Hessler 1970). The mouthparts in general are complex structures adapted to the lifestyle and feeding mechanisms of the species. Thus, the characters of the mandible are regarded to be phylogenetic informative. Specializations of mandibular structures (here a simplified incisior process) are correlated with other features as in *Echinopleura* where the lacinia mobilis is reduced to a small tooth (character 37).

**40) O Incisor process with strong shelf-like tooth. [Incisior process not shelf-like.]** (*Disparella, Momedossa*)

In this kind of incisior process, the teeth seem to be fused into one extremely strong enlarged shelf-like tooth, which is regarded as the apomorphic character state.

**41) O Incisor process enlarged (almost twice as long as lacinia mobilis). [Incisior process not enlarged.]** (*Disparella*)

This may be regarded as apomorphy of *Disparella,* as such an enlargement of the incisor process is unique for species of this genus.

**42) O Mandibular palp absent. [Mandibular palp present.]** (character 11 in chapter 4.1.1)

The presence or absence of the mandibular palp is a weak character in the families Desmosomatidae and Nannoniscidae; although the *absence of the mandibular palp* is presented as an autapomorphy of Macrostylidae by Wägele (1989). According to Wägele (1989), the presence of the mandibular palp is the plesiomorphic condition in Desmosomatidae and Nannoniscidae. In both families there are genera that include species with and without a mandibular palp (in *Mirabilicoxa* some species possess a palp and some do not, whereas in *Exiliniscus* no species possesses a palp).

**43) O Mandibular palp consisting of two articles. [Mandibular palp not consisting of two articles.]** (*Paradesmosoma, Desmosoma latipes, D. ochotense*)

A reduced third article of the mandibular palp occurs in all species of *Paradesmosoma*. Therefore, it is hypothesized that the reduction is an apomorphy of the genus although the third article is reduced, most possibly convergently, in other species of Desmosomatidae as well.

**Maxilliped**

**44) O Retinaculae elongated: more than 3 times longer than width of stalk. [Retinaculae not elongated: about 2 times longer than width of stalk.]** (*Thaumastosoma*, *Reductosoma*)

Such an elongation of the retinaculae is known only for the genus *Thaumastosoma*. No other desmosomatid, nannoniscid or macrostylid species possesses such long retinaculae. In *Reductosoma gunnera* Brandt, 1992, the retinaculae are longer than usual, but not as elongated as in *Thaumastosoma*. Species of *Thaumastosoma* have extremely highly specialized mouthparts and the elongated retinaculae are one of the complex patterns of this specialization.

**45) O Mouthparts extremely bent forward. [Mouthparts not bent forward.]** (*Thaumastosoma, Echinopleura cephalomagna*)

From a lateral view, the cephalothorax tapers to the tip of the maxilliped palp, covering the mouthparts. It is difficult to decide what the plesiomorphic condition of the position of the mouthparts is. In *Thaumastosoma* the head is characterized by the mouthparts bent extremely forward, forming a “tip” from lateral view. This condition is also found in *Echinopleura cephalomagna*, most possibly as the result of convergent evolution. However, the extremely modified mouthparts are a complex character that include more than one phylogenetically informative character.

## Pereonites

**46) O Stout sensory setae present anteriorly on tergits 1-4. [No sensory setae present anteriorly on tergits 1-4.]** (“nannoniscid character”)

Most nannoniscid species possess spines on the tergits. Such spines on the tergits do not occur in Desmosomatidae or Macrostylidae. The anteriorly directed spines on the tergits may be an autapomorphy of a group of species in Nannoniscidae *sensu* Siebenhaller & Hessler 1981.

**47) O Pereonite 1 broader than pereonite 2. [Pereonite 1 not broader than pereonite 2.]** (“desmosomatid character”)

(see character 48)

**48) O Pereonite 1 longer than pereonite 2 (midsagital length). [Pereonite 1 not longer than pereonite 2.]** (Eugerdellatinae)

The enlargement of pereonite 1 is dependent on the need of musculature for a modified robust pereopod. Most species with a robust or chelate first pereopod possess an enlarged pereonite 1. When in the evolution of the families Nannoniscidae and Desmosomatidae was a robust or chelate first pereopod developed? The similar size of the pereonites is here regarded as a plesiomorphy as well as in the following character (49).

**49) O Pereonite 1 enlarged and clearly bigger (more than 2 times of midsagital length of pereonite 2). [Pereonite 1 not enlarged and clearly bigger than pereonite 2.]** (*Eugerdella* species group 1)

The extreme enlargement of pereonite 1 is found in *Eugerdella* species. It is regarded as an autapomorphy for this group.

**50) O Pereonite 1 shorter and not as broad as pereonite 2. [Pereonite not shorter than pereonite 2.]** (species of *Eugerda* and *Desmosoma*)

The reduction or attenuating of pereopod I is often combined with a reduction in size of pereonite 1 (species of *Eugerda*) although this dependence is not always given (species of *Eugerda* and *Desmosoma*). Thus, equal size of pereonite 1 and 2 is the plesiomorphic state, which also relates to the groundpattern of Janiroidea.

**51) O Spine-like ventral elongations at pereonites 1 to 5 decreasing in length towards the posterior pereonites. [Pereonites 1 to 5 without spine-like ventral elongations.]** (*Eugerdella pugilator* and *E. serrata*)

The presence or absence of a ventral elongation at the pereonites should be treated as single character for each pereonite. If a common ancestor of Macrostylidae, Nannoniscidae and Desmosomatidae is hypothesized to have ventral elongations at the pereonites, the absence of the elongations is a reduction. Here it is postulated that the common ancestor did not possess ventral elongations. Although, the presence of ventral elongations at the pereonites is regarded as synapomorphy of the three families by Wägele (1989). (character 15)

When comparing the ventral elongations which occur in all three families, it becomes obvious that they are located at different pereonites and that their shape varies: the nannoniscid species *Regabellator profugus* and *R. abyssi* possess ventral elongation at the fused pereonites 6 and 7 (character of the generic diagnosis of *Regabellator*), the elongation at pereonite 6 is directed anteriorly, the one at pereonite 7 caudally. The nannoniscids *Rapaniscus dewdneyi*, *R. crassipes* possess a caudally directed strong ventral elongation on pereonite 7, but not at the dorsally fused pereonites 6 and 7. In addition to these characters, all *Rapaniscus* species possess a specialized antennula with a bulbous last article. *Nannoniscus teres* possesses a caudally directed strong spine on pereonite 7, *N. analis* a curved caudally directed spine on the operculum and *N. bidens* and *N. antennaspinis* a straight, but caudally directed spine on the operculum. Within *Nannoniscus*, species with and without opercular spine are grouped together. *Saetoniscus meteori* possesses an opercular spine. In *Thaumastosoma*, a straight spine on pereonite 7 and a strong midventral spine on the operculum are present. The spines of *Nannoniscus* are more anteriorly positioned on the operculum as they are in *Saetoniscus meteori*.

Opercular spines also occur in Macrostylidae. *Macrostylis longipedis* possesses a straight opercular spine tapering to the posterior part of the operculum, a short straight ventral spine on pereonite 1, and a slightly curved caudally directed spine on pereonite 2. In *M. abyssalis*, ventral caudally directed elongations are present at pereonites 5 and 6 and in *M. longispinis* at pereonites 6 and 7. However, the elongation on pereonite 7 is stronger than the one on pereonite 6.

The desmosmatid species *Prochelator lateralis* and *P. hampsoni* possess an anteriorly directed spine at pereonite 1. In *P. lateralis* ventral caudally directed spines also occur on pereonite 2-4. *Disparella kensleyi* sp. nov. bears an anteriorly directed ventral elongation at pereonite 5 and the *Eugerdella* species *E. pugilator* and *E. serrata* posses anteriorly directed ventral elongation at pereonites 1-5 which decrease in length towards the posterior pereonites.

With regard to the differences in the families, one might homologize the ventral elongations present in Desmosomatidae at pereonites 1-5 (with the exception of *Disparella kensleyi*), a second group of ventral elongations at the pereonites in Nannoniscidae, a third group of ventral elongations at the operculum and - as a fourth group - the ventral elongations occurring in Macrostylidae. In the present analysis, it is decided to treat each type of spine or involvement of ventral elongations in a character complex as a single character (characters 51 to 57). For the macrostylid spines, no characters are defined because they were previously defined as an outgroup due to 10 other characters. Further, the spines occurring in Macrostylidae seem to have evolved convergently compared to those found in Nannoniscidae and Desmosomatidae.

**52) O Anteriorly directed spine at pereonite 1. [Pereonite 1 smooth ventrally.]** (*Prochelator lateralis* and *P. hampsoni, P. uncatus*)

(see character 51)

**53) O Spine-like ventral elongation at the fused pereonites 6 and 7, the elongation at pereonite 6 directed anteriorly, the one at pereonite 7 caudally. [Pereonites 6 and 7 without fusion or spine-like elongation.]** (*Regabellator* species: *R. profugus* and *R. abyssi*)

(see character 51)

**54) O Ventral spine midway on the operculum. [Operculum without ventral spine.]** (*Rapaniscus multisetosus*)

(see character 51)

**55) O Ventral caudally directed strong spine on pereonite 7. [Pereonite 7 smooth ventrally.]** (*Nannoniscus teres*, *Thaumastosoma platycarpus* and *T. tenue*, *Rapaniscus dewdneyi*, *R. crassipes* and *R. multisetosus*)

(see character 51)

**56) O Ventral curved caudally directed spine located midway on the operculum. [Operculum without spine.]** (*Nannoniscus analis*, *Thaumastosoma platycarpus* and *T. tenue*)

(see character 51)

**57) O Ventral straight, caudally directed spine positioned anteriorly on the operculum. [Operculum without spine.]** (*Nannoniscus antennaspinis,* *N. bidens,* *Saetoniscus meteori*)

(see character 51)

**58) O Pereonites 6 and 7 fused. [Pereonites free.]** (“nannoniscid character” *Saetoniscus*, *Rapaniscus*, *Regabellator*, *Nannoniscus*, *Hebefustis*, *Nannoniscoides, Gen. nov.,* new species A)

The fusion of the pereonites is a character occurring in Nannoniscidae and can be regarded as an apomorphy, while free pereonites are the plesiomorphic condition in the asellotan groundpattern and in closely related groups.

**59) O Pereonites 6 and 7 fused with pleotelson. [Pereonites and pleotelson free.]** (“nannoniscid character”, *Nymphodora fletcheri*)

This character state occurs in gen. nov. *fletcheri* (Paul & George 1975; Kaiser 2009). In no other nannoniscid species the posterior two pereonites (6 and 7) *and* the pleotelson are fused. This character is regarded as an autapomorphy and may have evolved out of a fusion of pereonites 6 and 7 only, secondarily involving the pleotelson or from a fusion of the pleotelson with pereonite 7, secondarily involving pereonite 6. Since the pleotelson contains fused pleonites, it is hypothesized that the fusion first included pereonite 7 and the pleotelson at first and then pereonite 6.

**60) O Pereonite 7 and pleotelson fused. [Pereonites and pleotelson free.]** (“nannoniscid character” *Nannonisconus*)

Only pereonite 7 and the pleotelson are fused in species of *Nannonisconus*. The character is regarded as an apomorphy of this genus.

**61) O Pereonites 6, 7 and pleotelson with marginal flanges. [Pereonites 6, 7 and pleotelson without marginal flanges.]** (*Mirabilicoxa*, *Eugerda*, *Desmosoma, Thaumastosoma*)

Marginal flanges occur in different genera of Desmosomatidae representing a character also known as sexual dimorphic and occurs in females and males. Therefore, it is difficult to decide whether it evolved convergently or not. Although the character appears to be weak, it is used in the analysis. It is hypothesized that the marginal flanges may be used as apomorphy to define a subgroup within a genus.

**Pereopods**

**62) O Coxae 1-4 with anterolateral elongations [Coxae without anterolateral elongations.]** (*Mirabilicoxa*)

In the generic diagnosis of *Mirabilicoxa* presented by Hessler (1970), the produced coxae are regarded as an autapomorphy of the genus. This is probably true, because they are not known for any other genus of Desmosomatidae. In females, the projections are not as strong as in males. In Nannoniscidae and Macrostylidae, such coxal projections are absent.

**63) O Coxae 1-4 anteriorly tipped with stout seta. [No stout seta present on anterior tip of coxae 1-4]** (“desmosomatid character”)

The presence of setae on the tip of the coxal projections is regarded as a desmosomatid character (contrary to character 39). The presence of a sensory spine is defined as apomorphy. Taxa without a projection usually posses a seta on the angular anterior tip of the coxa.

## 64) O Coxae produced anteriorly. [Coxae angular, without projection.] (“desmosomatid character”)

In the janiroidean groundpattern, coxae are not produced anteriorly. Such a projection is regarded as an apomorphy.

**65) O Pereopods I, II, VI and VII longer than pereopods III to V. [Pereopods of similar length.]** (“macrostylid character”)

This character was considered to be a macrostylid character in the literature (e.g. Siebenhaller & Hessler 1981). In most nannoniscid species all pereopods are of similar length. Species of *Thaumastosoma* and *Pseudomesus* in addition to most Desmosomatidae possess posterior swimming legs that are slightly longer than the anterior pereopods.

**66) O Pereopods V to VII longer and more heavily built than pereopods II to IV. [Pereopods V to VII and pereopods II to IV of similar length.]** (*Eugerda*, *Desmosoma,* *Torwolia*, *Desmosoma*)

The plesiomorphic condition is a similar length of the pereopods. A difference in strength (length) is regarded as apomorphy.

**67) O Pereopods V-VII: Ischium elongated (more than 5.5 times longer than wide). [Pereopods V-VII not with elongated ischium.]** (*Eugerdella hessleri*, *E. ischnomesoides*)

Such an elongation of the ischium occurs in no other species of Desmosomatidae or Nannoniscidae. This characterizes these two species as sister taxa.

**68) O Pereopod III dorsally bent. [Pereopod III not dorsally bent.]** (“macrostylid character” 9)

When comparing the pereopod III of macrostylid species with pereopods of desmosomatid or nannoniscid species, the macrostylid dactyli are dorsally bent (and the dorsal margin has long setae). This is regarded as an autapomorphy of Macrostylidae (Wägele 1989).

**69) O Dactylus of pereopod III with row of long setae. [Dactylus of pereopod III without row of long setae.]** (“macrostylid character” 10)

This character is an autapomorphy of Macrostylidae according to Wägele (1989).

**70) U Ventral row of natatory setae at pereopods V to VII absent. [Ventral row of natatory setae present.]** (“nannoniscid character” 16)

Ventral rows of setae were presented as an autapomorphy of Desmosomatidae by Wägele (1989). Thus, Nannoniscidae are regarded as a more derived group. Consequently, the presence of ventral rows of setae in some nannoniscid species (for example at carpus and propodus of pereopod VII in the *Pseudomesus* and *Thaumastosoma* species) leads to the hypothesis of a reduction of these setae in Nannoniscidae. It may also be possible, that the group of species without natatory setae on the posterior limbs is the group that has the plesiomorphic condition. Consequently, natatory setae evolved once in Nannoniscidae and Desmosomatidae regarding the two families as one group. In this case, the species with the ability to swim would be more derived (most desmosomatids).

**71) O Basis of pereopod VII with long setae. [No long setae on basis of pereopod VII present.]** (“macrostylid character”)

The “swimming setae” at the basis of pereopod VII in Macrostylidae do not occur in Desmosomatidae and Nannoniscidae (Brandt 2004). In contrast to these two families, Macrostylidae possess robust setae on propodus and carpus. This kind of setation may be regarded as an apomorphy, which distinguishes Macrostylidae from Desmosomatidae, Nannoniscidae and other Janiroidea.

**Pereopod I^[[3]](#footnote-3)^***

**72) U Ventral margin of carpus of pereopod I with composed robust setae in a row. [Ventral margin of carpus of pereopod I not with composed robust setae in a row.]** (“desmosomatid character” 18)

This character is one of the apomorphies of Desmosomatidae as presented by Wägele (1989) Setae standing in a row means more than three setae inserting very close and in regular distances to each other increasing in size towards the distal setae of the article. Hessler (1970) used the term “major setae”. Composed robust setae are present in Macrostylidae, but are not lined-up in clear rows. In some Nannoniscidae, composed robust setae are lined-up in a row (for example on the carpus and propodus of the robust pereopod I of the three *Rapaniscus* species). In Desmosomatidae these rows tend to be reduced in the genera *Desmosoma*, *Eugerda* and *Mirabilicoxa* as well as in those genera where pereopod I has a chela (*Chelator*, *Prochelator*, *Disparella*, *Oecidiobranchus*). These reductions are regarded as apomorphies of the single groups, while within Desmosomatidae composed setae lined-up in rows are the plesiomorphic condition (defined in character 73). In Desmosomatidae the setae tend to be composed and long while in Nannoniscidae, if present, the setae in the row are composed and short.

**73) O Pereopod I: ventral row of setae on carpus reduced due to specialization. [Pereopod I: ventral row of setae on carpus not reduced due to specialization.]** (“desmosomatid character”)

(see character 72)

**74) O Carpus of pereopod I with a row of long simple setae dorsally. [Carpus of pereopod I without a row of long simple setae dorsally.]** (“desmosomatid character”).

Reduction of this dorsal row is present in desmosomatid species depending on the specialization of pereopod I. These reductions are defined as an apomorphy in character 75.

**75) O Pereopod I: dorsal row of setae on carpus reduced due to specialization. [Pereopod I: dorsal row of setae on carpus not reduced due to specialization.]** (“desmosomatid character”)

(see character 74)

**76) O Enlargement of pereopod I concentrating on carpus. [Enlargement of pereopod I not concentrating on carpus.]** (*Prochelator, Chelator, Disparella, Eugerdella* species*, Mirabilicoxa cornuta, Paradesmosoma, Reductosoma, Rapaniscus*)

The enlargement of pereopod I correlates with the enlargement of the carpus. This occurs in the group of species possessing a carpo-euchelate or a raptorial pereopod I. A heavy carpus seems to be essential in both forms. Thus, the concentration on the enlargement of the carpus is defined as apomorphy for this group of species.

**77) O Enlargement of pereopod I concentrating on propodus. [Enlargement of pereopod I not concentrating on propodus.]** (*Eugerdella species*, *Paradesmosoma, Prochelator, Reductosoma, Oecidiobranchus, Cryodesma*)

This character is defined as apomorphy for the species possessing a raptorial pereopod I due to the hypothesis, that its function makes the evolution of the enlarged propodus necessary.

**78) O Pereopod I small and slender, but propodus enlarged. [Pereopod I not small and slender with enlarged propodus.]** (*Torwolia*)

In *Torwolia* the propodus is modified to form a subchela and differs in form and shape from the kind of enlargement found in other desmosomatids. Furthermore, the other articles and specialization of pereopod I in *Torwolia* resembles more the trend found in *Eugerda* and some *Desmosoma* species.

**79) O Pereopod I as functional unit enlarged. [Pereopod I as functional unit not enlarged.]** (Eugerdellatinae, *Rapaniscus* species)

Eugerdellatinae are regarded as a monophyletic group due to the enlargement of pereopod I leading to different forms of specialization in this subfamily. Thus, this character is regarded to be an apomorphy for all species included in Eugerdellatinae. In some species of Nannoniscidae an enlarged pereopod I evolved convergently.

**80) U Propodus of pereopod I ventrally with row of small stout unequally bifid setae. [Propodus of pereopod I ventrally not with row of small stout unequally bifid setae.]** (*Eugerdella* species*, Hebefustis, Nannoniscus, Chelantermedia., Rapaniscus*)

Within species possessing a raptorial pereopod small stout unequally bifid setae occur on the ventral margin of the propodus. This is regarded as an apomorphy related to the function of the limb, as such a row does not occur in any other of the five forms of pereopod I. Since the apomorphic character state also occurs in some nannoniscid genera, it may be possible, that such a row evolved more than once. The character is defined “unordered” due to the possibility of convergent evolution or reduction of the row.

**81) O Platform-like gap between propodus and distoventral seta on carpus present. [Platform-like gap between propodus and distoventral seta on carpus absent.]** (*Eugerdella serrata, E. pugilator, E. theodori*)

This platform-like gap occurs in the extremely enlarged forms of a raptorial pereopod I only and seems to act as a stopping mechanism for the movement of the propodus. Thus, it is regarded as an apomorphy.

**82) O Carpus of pereopod I enlarged and tapering towards propodus. [Carpus of pereopod I not enlarged, not tapering towards propodus.]** (*Rapaniscus*)

An enlarged carpus tapering towards the propodus is the result of convergent evolution of the enlarged pereopod I within Nannoniscidae. This form of the carpus is regarded as apomorphy defining the *Rapaniscus* species.

**83) O Propodus of pereopod I ventrally fringed with fine hairs and setae connected by a cuticular membrane. [Propodus of pereopod I ventrally not fringed with fine hairs and setae connected by a cuticular membrane.]** (all species with a carpo-euchelate pereopod I)

A fringed ventral margin occurs in all species possessing a carpo-euchela. Thus, it is hypothesized, that there is a functional need of such a structure for the function of the chela. The character state is defined as apomorphic for this group of species. In some species belonging to *Momedossa*, *Desmosoma* the ventral margin of the propodus may be fringed with a cuticular membrane, but there are no setae breaking through this membrane as defined for this character.

**84) O Carpus of pereopod I enlarged and broadest at articulation to propodus. [Carpus of pereopod I not enlarged and not broadest at articulation of propodus.]** (new species A, *Paradesmosoma*, *Oecidiobranchus, Prochelator, Chelator, Disparella*)

In the specialized pereopod I forming a carpo-euchela, the width of the carpus gives the propodus more room at the articulation and the propodus is wider at its posterior articulation than at its anterior articulation with the dactylus. The carpus being broadest anteriorly is defined as an apomorphic character state.

**85) O Carpus distolaterally with “claw-seta”. [Carpus distolaterally not with a “claw-seta”.]** (*Oecidiobranchus, Prochelator, Chelator, Cryodesma, Disparella*, *Paradesmosoma, Reductosoma*)

The distal seta of the ventral row of the carpus is extremely enlarged reaching the full length of the ventral margin of the propodus or is even slightly longer than this. The claw-seta acts as an antagonist to the propodus forming a grasping organ. The evolution of these patterns is so complex, that it is most likely, that such a structure evolved only once. Consequently, the group of species possessing a carpo-euchela can be defined as a monophyletic group.

**86) O Carpus distolaterally produced. [Carpus distolaterally not produced.]** (species of *Chelator,* *Prochelator* and *Disparella*, *Reductosoma gunnera*)

In some species of the carpo-euchelate genera the carpus is produced at the base of the claw-seta. This may support the function of the claw-seta and is therefore defined as apomorphic character state. However, this character appears to be weak, as it is difficult to distinguish between a production and only a bulge due to the insertion of the large claw-seta, but was presented in the generic diagnosis of *Chelator* (Hessler 1970).

**87) O Carpus of pereopod I with one composed seta midway. [Carpus of pereopod I not with one composed seta midway.]** (*Prochelator, Reductosoma*)

The composed midventral seta is presented as character within the generic diagnosis of *Prochelator* (Hessler 1970). Some species of the genus possess a composed seta midway, others a distally setulate seta, which is also described as “accessory seta” by Hessler (1970). Such a midventral seta occurs not only in species of the genus *Prochelator*, but also in *Reductosoma gunnera* and new species A. It may be regarded as an apomorphy of this group of species.

**88) O Ventral setae behind claw-seta small and simple or small and slender. [Not with ventral setae behind claw-seta small and simple or small and slender.]** (*Chelator, Disparella*)

This combination of characters does only occur in species of *Chelator* and *Disparella*. It is regarded to be an apomorphy of this group of species.

**89) O Carpus of pereopod I enlarged and with setae of irregular size. [Carpus of pereopod I not enlarged, not with setae of irregular size.]** (*Paradesmosoma*, *Eugerdella*)

The apomorphy was used for the generic diagnoses of *Eugerdella* modified by Hessler (1970) and *Paradesmosoma* as presented by Kussakin (1965). The species of these two genera are the only species possessing setae of irregular size. Thus, this is regarded as an apomorphy of *Paradesmosoma* and a group of species within *Eugerdella*.

**90) O Size of ventral setae on carpus irregular and of varying types. [Size of ventral setae on carpus not irregular, of same type.]** (*Paradesmosoma*)

This character distinguishes the species of *Paradesmosoma* from the *Eugerdella* species. No other desmosomatid or nannoniscid species has this character. It clearly defines the group of species united within *Paradesmosoma*. Size of ventral setae on carpus irregular and of varying types is regarded as an apomorphy of the genus.

**91) O Setae behind claw-seta small, of similar size and type. [Carpus of pereopod I not with claw-setae and setae not behind claw-seta small, of similar size and type.]** (some *Disparella species*, *Chelator species*)

This combination of setae defines a group of species in *Chelator* and *Disparella*. The regular size combined with the claw-seta is unique for this group of species and therefore can be regarded as apomorphic character state.

**92) O Carpus distoventrally with claw-seta and penultimate seta. [Carpus not with claw-seta and penultimate seta.]** (*Prochelator*, *Chelator*, *Oecidiobranchus, Reductosoma, Disparella* and new species A)

This apomorphy characterizes the group of species with a carpo-euchela and supports the monophyly of this group.

**93) O Pereopod I robust, articles almost quadrangular. [Pereopod I not robust, articles not quadrangular.]** (*Whoia*, *Thaumastosoma*)

Species of *Whoia* and *Thaumastosoma* possess limbs very similar to the form hypothesized for the janiroidean groundpattern, but the limbs are more robust and bear rows of large composed setae. This robust form of the limb is regarded as an apomorphy.

**94) U Setae in ventral row on carpus of pereopod I increasing in length towards propodus. [Setae in ventral row on carpus of pereopod I not increasing in length towards propodus.]** (“desmosomatid character”)

In most species the setae in the ventral row are increasing in length towards the propodus. This condition is not found in nannoniscids. Thus, it is regarded as desmosomatid character, although in many species these rows are reduced. Due to this fact, the character is defined as “unordered”.

**95) O Setae on carpus and propodus of pereopod I not composed. [Composed setae present on carpus and propodus of pereopod I]** (*Desmosoma*, *Eugerda, Austroniscus, Regabellator, Nannoniscoides*)

The composed setae may have been reduced several times in both families. Therefore, this is defined as apomorphic character state.

**96) O Distoventral seta on carpus of pereopod I shortest. [Distoventral seta of carpus of pereopod I not shortest.]** (*Eugerdella* species*, Mirabilicoxa cornuta*)

This character defines the genus *Eugerdella* as presented by Hessler (1970), but the genus can be regarded as paraphyletic. It is quite possible that a short distal seta evolved convergently. However, here it is used as apomorphic character state, because it is hypothesized that this may clarify the relationship in terminal taxa and support the relationship between sister taxa.

**97) O Distoventral seta of carpus reaching full length of propodus. [Distoventral seta of carpus not reaching full length of propodus.]** (all species with a carpo-euchelate pereopod I as well as *Cryodesma*, *Whoia angusta* and *Thaumastosoma platycarpus*)

This apomorphy defines species with enlarged distal setae and the group of species bearing a carpo-euchelate pereopod I. It is hypothesized, that the large size of the distal setae evolved first and therefore is the plesiomorphic condition for the species bearing a carpo-euchela.

**98) O Second seta behind claw-seta of similar size. [Not with second seta behind claw-seta of similar size.]** (*Cryodesma*)

Species included in *Cryodesma* bear a ventral row of long composed setae on the carpus of pereopod I as well as a seta very similar to a claw-seta. The similarity in size of the setae in the row is regarded as an apomorphy of the genus (Svavarsson, 1988).

**99) O Pereopod I slender in comparison to pereopod II. [Pereopod I not slender in comparison to pereopod II.]** (*Desmosoma, Echinopleura, Eugerda, Mirabilicoxa, Torwolia*)

Here, the specialization of pereopod I with the trend to an attenuated condition is described. A slender pereopod I is defined as apomorphic character state.

**100) O Pereopod I slender and ventrally only slender setae present on carpus and propodus. [Pereopod I not slender and not only slender setae present on carpus and propodus.]** (*Desmosoma*, *Eugerda*)

This condition is only found in species of *Desmosoma* and *Eugerda* and is defined as an apomorphy for this group of species. The composed setae are hypothesized to be reduced.

**101) O Pereopod I small in size, subchelate: propodus enlarged and dactylus folding against propodus. [Pereopod I not small in size, not subchelate.]** (*Torwolia*)

A subchela is unique for species of *Torwolia*. Thus, this condition is defined as an apomorphy of the genus.

**102) O Propodus of pereopod I elongated in chela. [Propodus of pereopod I not elongated in chela.]** (*Disparella*)

An elongation of the propodus in the carpo-euchela of pereopod I is only found in species of *Disparella* and is defined as an apomorphy of the genus.

**103) O Propodus of slender pereopod I elongated (over 3.5 times longer than wide). [Propodus of pereopod I not elongated.]** (*Desmosoma* species*, Echinopleura, Eugerda, Mirabilicoxa*)

This character defines a group of species in the subfamily Desmosomatinae. For the species characterized by this apomorphy a close relationship is hypothesized. The elongation of the propodus leads to a transformation series that ends in an extremely attenuated pereopod (characters 104-106).

**104) O Pereopod I slender: propodus between 4.1 and 5.2 times longer than wide, carpus about 4.5 times longer than wide. [Pereopod I not slender.]** (*Desmosoma*)

*Desmosoma gigantea* propodus 4.1 times longer than wide; carpus 4 times longer than wide, *D. latipes* propodus 5.2 times longer than wide; carpus 4.8 times longer than wide. For discussion see character 103.

**105) O Pereopod I slightly attenuated: propodus of pereopod I between 6 and 9 times longer than wide and carpus between 5 and 7.2 times longer than wide). [Pereopod I not slightly attenuated.]** (*Eugerda*)

*Eugerda intermedia* propodus 8.6 times longer than wide; carpus 7.2 times longer than wide. *E. elegans* propodus about 9 times longer than wide, carpus about 5 times longer than wide, *E. arctica* propodus 6.5 times longer than wide, carpus 5.6 times longer than wide, *E. anversense* propodus about 6 times longer than wide, carpus about 7 times longer than wide. For discussion see character 103.

**106) O Pereopod I strongly attenuated (propodus 18.8 times longer than wide, carpus 15 times longer than wide), setae absent on propodus and carpus. [Pereopod I not strongly attenuated, setae present on propodus and carpus.]** (*Eugerda*)

*Eugerda reticulata* (propodus 18.8 times longer than wide, carpus 15 times longer than wide), *Eugerda fulcimandibulata* (propodus 15.2 times longer than wide, carpus 11.8 times longer than wide). For discussion see character 103.

## Pereopod II

**107) O Pereopod II robust, articles almost quadrangular. [Pereopod II not robust, articles, not quadrangular.]** (*Whoia*)

In species of *Whoia* pereopod II is as robust as pereopod I. The first two limbs resemble (compare character 93) each other so much that the similarity was presented in the generic diagnosis by Hessler (1970). Such a robust pereopod II occurs in no other species of Desmosomatidae or Nannoniscidae. It is regarded here as an apomorphy of the genus.

**108) O Propodus of pereopod II heavily built. (carpus and propodus broad). [Propodus of pereopod II not heavily built.]** (*Torwolia*, *Eugerda*, *Desmosoma*)

In some species of *Desmosoma* and *Eugerda* pereopod II is specialized to an enlarged and strong limb. This correlates with the reduction of size of pereopod I and sometimes with an enlargement of pereonite 2, e.g. in *Torwolia*. A heavily built pereopod II is defined as an apomorphy.

**109) O Carpus of pereopod II bearing a ventral row of composed setae. [Setae on carpus of pereopod II not standing in ventral rows.]** (desmosomatid character 19 chapter 4.1.1)

This character is part of a complex autapomorphy presented by Wägele (1989). The other parts are defined in characters 110 and 111. Composed setae occur on pereopod II in Macrostylidae, Desmosomatidae and Nannoniscidae. The presence of composed setae can be regarded as a plesiomorphy. In Macrostylidae these setae are not standing in a row. In most species of Nannoniscidae, these setae are not sanding in a row. The presence of a row has to be defined as an apomorphy.

**110) O Propodus of pereopod II bearing a ventral row of composed setae. [Setae on carpus and propodus of pereopod II not standing in rows ventrally.]**

(see character 109, second part of the autapomorphy.)

**111) O Carpus and propodus of the pereopod II bearing dorsally a row of long setae. [Setae on carpus and propodus of pereopod II not standing in rows dorsally.]** (see character 109, third part of the autapomorphy.)

**112) O Basis and ischium of pereopods II and III fringed with distally plumose setae. [Basis and ischium without this setal type.]** (compare characters 113 and 114, *Paradesmosoma*) This type of seta occurs only in the genus *Paradesmosoma.*

## Pereopod IV

**113) O Pereopod IV folious, carpus and propodus paddle-like. [Pereopod IV not folious, carpus and propodus resembling carpus and propodus of pereopod III.]** (*Paradesmosoma*)

The species of the genus *Paradesmosoma* possess a very specialized pereopod IV – a character, which distinguishes the species of the genus from all other desmosomatids and nannoniscids. Hessler (1970) described the fourth pereopod to be unique for this genus and suggested an origin separate from that of the chelate forms.

**114) O Carpus and propodus surrounded by (with a dense row of) numerous distally plumose setae^[[4]](#footnote-4)^. [Carpus and propodus not surrounded by numerous distally plumose setae.]** (*Paradesmosoma*)

This type of seta (see character 112) occurs only within the species of the genus *Paradesmosoma*. The setae are surrounding the propodus and carpus so characteristically, that this can be defined as an apomorphy of the genus. The plesiomorphic character state is a ventral and dorsal row of setae (composed or slender setae) as in other desmosomatid species which bear a pereopod IV that resembles pereopod III.

**Pereopod VII**

**115) O Ischium dorsally with anteriorly directed cuticular hook. [Ischium dorsally smooth.]** (*Pseudomesus*)

The dorsal hook on the ischium is a cuticular structure appearing in the species of *Pseudomesus* only and can therefore be regarded as an autapomorphy of this genus.

**116) O Propodus and carpus of pereopod VII with long setae dorsally. [No long setae dorsally of propodus and carpus of pereopod VII.]** (“desmosomatid character”)

The evolution of natatory setae on the carpi and propodi of the posterior pereopods is presented as an autapomorphy of Desmosomatidae in the literature (Hessler 1982, Wägele 1989). While some species possess a condition “between” like *Thaumastosoma platycarpus* or *Pseudomesus satanus* or the natatory setae are completely absent in other nannoniscid species. Wägele (1989) hypothesized a common ancestor of Munnopsididae, Macrostylidae, Desmosomatidae and Nannoniscidae that was able to dig and swim with the posterior pereopods. Consequently, Macrostylidae lost the natatory setae, while in Desmosomatidae the natatory setae evolved as a row and these rows were reduced in Nannoniscidae, the most derived group according to Wägele (1989). Regarding the relationships of the families in figure 115 based on molecular data (Raupach *et. al.* 2004), another hypothesis could be that Macrostylidae never had swimming setae and these setae have evolved in Munnopsididae and Desmomatidae (including Nannoniscidae).

**117) O Propodus and carpus of pereopod VII with long setae dorsally. [No long setae dorsally of propodus and carpus of pereopod VII.]** (“desmosomatid character”, see character 116)

**118) O Basis of pereopod VII with long slender “swimming setae”. [Basis of pereopod VII without “swimming setae”.]** (“macrostylid character”)

According to Brandt (2004), the presence of these setae is regarded as a macrostylid autapomorphy.

### Pleotelson

**119) O Pleotelson dorsally inflated. [Pleotelson dorsally not inflated.]** (*Pseudomesus*)

The dorsal inflation of the pleotelson is the most typical feature of *Pseudomesus* species next to the extremely short uropods. It is probably an apomorphy of the genus.

**120) O Anus region separated and bilobed. [Anus region not separated and bilobed.]** (*Nannonisconus*)

This shape of the anus region is unique for species of this genus. Thus, it is defined as its apomorphy.

**121) O Pleotelson vaulted in transverse section. [Pleotelson not vaulted in transverse section.]** (*Chelator*, *Oecidiobranchus, Whoia* species)

This condition was defined as generic character of *Oecidiobranchus* by Hessler (1970). It also occurs in *Chelator antarcticus* sp. nov. and some *Whoia* species. In *Oecidiobranchus* there may be a correlation of the form of the pleotelson and the small opercular chamber (character 123). This form of the pleotelson may indicate a close relationship of *Oecidiobranchus* and *Chelator*. Both genera possess a carpo-euchela on pereopod I.

**122) O Branchial chamber and operculum in relation to size of pleotelson small, operculum of oval shape and posterior part broadest. [Branchial chamber and operculum covering nearly the whole pleotelson ventrally.]** (*Austroniscus*)

This condition of the branchial chamber in *Austroniscus* (Nannoniscidae) differs from the form of a small branchial chamber as it occurs in *Oecidiobranchus* (character 123); in *Austroniscus* the branchial chamber is small with regard to the flat lateral body extension. This is unique for species of this genus and therefore defined as apomorphy.

**123) O Branchial chamber and operculum in relation to size of pleotelson small, rounded. [Branchial chamber not small and rounded.]**

(*Oecidiobranchus*)

A very small operculum occurs also in species of *Austroniscus*, which are defined by other autapomorphies like the lateral elongation and the flattening of the tergits. In *Oecidiobranchus*, the size of the branchial chamber is small in comparison to the body without regard to any flanges. Therefore, it is possible, that a small branchial chamber evolved convergently within these two taxa. Here, both character states are presented as an apomorphy due to the different shapes of the branchial chamber and the operculum (character 122). It is considered to be useful to define the systematic position of the species of the two genera.

**124) O Uropods uniramous. [Uropods biramous.]**

Macrostylidae possess very characteristic uropods: uniramous with elongated articles. In most cases, the uropods are much longer than the length of the pleotelson. It is hypothesized that the reduction of the exopod took place first, and then the elongation followed as evolutionary step. Reduction of the exopod occurs frequently within Desmosomatidae while most Nannoniscidae possess biramous uropods (exception: *Pseudomesus*, *Nannoniscus ovatus* and *N. perunis*). Reduction of the exopod is not a phylogenetically useful character. The uniramous condition with the extreme elongation found in Macrostylidae can be regarded as a macrostylid autapomorphy (character 125).

Hessler (1970) described five stages of reduction and presented examples from different species. In his opinion, the species possessing a reduced exopod indicate how this structure might have got lost. The loss of the exopod cannot be regarded as a transformation series, as long as species with a reduced exopod are not characterized by any other synapomorphy. The description of different „stages of reduction“ is most possibly a description of convergent evolution. The phylogenetic information of these reductions is doubtful, but the reduction may serve well as diagnostic character. In Desmosomatidae, the uropodal exopod does never reach the full length of the endopod.

**125) O Uropodal sympod extremely elongated; styliform. [Uropodal sympod not elongated, shorter than endopod.]** (“macrostylid character” 7)

This character is presented as an autapomorphy for Macrostylidae by Wägele (1989). The very characteristic uropods are described above (character 124).

**126) U Uropods cover anus valves. [Uropods not covering anus valves.]** (“nannoniscid character” 17)

Wägele (1989) presented this character as an autapomorphy for Nannoniscidae. The question is, how much of the anus valves are covered by the uropods or whether a very close insertion to the anus valves is the same. This character state is scored when the uropods are partly overlapping the anus valves (due to a close insertion next to them). The plesiomorphic state is given, if the uropods are inserting near the distal ends of the pleotelson. Within the phylogenetic analysis, groups may be defined by this character although it occurs also in some desmosomatid species, not only in Nannoniscidae.

**127) O Uropods short, not overlapping posterior margin of pleotelson. [Uropods not short, overlapping posterior margin of pleotelson.]** (“nannoniscid character” 17)

For using the apomorphic character state that was presented as an autapomorphy of Nannoniscidae by Wägele (1989), “uropods short” has to be defined clearly. The terminus “short” is defined here as not overlapping the terminal margin of the pleotelson (for example species of *Pseudomesus*). Thus, the plesiomorphic condition is that the uropods overlap the posterior margin of the pleotelson. However, the definition of this character remains weak. To define the length of the uropod, as morphometric relation its extension beyond the posterior margin of the pleotelson was used.

**128) O Uropodal endopod bulbous. [uropodal endopod clearly longer than wide.]** (*Pseudomesus*)

An extremely shortened, bulbous uropodal endopod occurs within species of *Pseudomesus*. This is regarded as an apomorphic condition while in the plesiomorphic state the uropodal endopod is longer than wide.

**129) O Uropodal exopod reduced to half of size of endopod or less. [Uropodal exopod not reduced to half of size of endopod or less.]**

For this character, convergent evolution is hypothesized in many taxa. Although this character appears to be weak, the reduction of the exopod is used as apomorphic state because it may solve the relationship in terminal clades.

1. Remark: The monophyly of *Nannoniscus* is questioned due to the high variability of shapes within the genus. Some species of *Nannoniscus* are flattened. Nevertheless, they are not included in the phylogenetic analysis, because of the lack of many details in the description that are necessary for the phylogenetic analysis. [↑](#footnote-ref-1)
2. Flagellar article 4 as last bulbous article occurs in *Micromesus* Birstein, 1963, a monotypic genus with the species *Micromesus nannoniscoides* Birstein, 1963, which is excluded from the phylogenetic analysis due to an incomplete species description and a questionable systematic position in Nannoniscidae (Siebenhaller & Hessler 1981, Wägele 1989, Kaiser 2005). Therefore, this character is not defined here. [↑](#footnote-ref-2)
3. * The functional unit of the propodus and carpus as well as the setation of the ventral margin of the carpus on pereopod I is a complex character. Each sub-character (pattern) is coded separately. The “claw-seta” can be homologized with the distal seta of the ventral row. The characters of pereopod I are mainly defined to distinguish between the related groups in Desmosomatidae because ventral and dorsal rows are not present in most species of Nannoniscidae. However, there are exceptions (character 18). [↑](#footnote-ref-3)
4. Ventral margins of ischium, merus, capus and propodus with setae having sturdy long bases, being abruptly more slender with flexible tips possessing fine setae. [↑](#footnote-ref-4)
