# Supplementary material for: Evolution and phylogeny of the deep-sea isopod families Desmosomatidae Sars, 1897 and Nannoniscidae Hansen, 1916 (Isopoda: Asellota)
Source: Org Divers Evol. 2021 Oct 13;21(4):691–717. doi: 10.1007/s13127-021-00509-9 (PMC8510888; doi:10.1007/s13127-021-00509-9)
Supplement: Supplementary file 3 — Supplementary file3 (DOCX 602 KB). Alist of all characters and their a priori weighting sensu Wägele (2004) is presented in Electronic Supplement 3. [file 13127_2021_509_MOESM3_ESM.docx]

**Morphological Phylogeny (Figure6A)**

**List of characters used in the phylogenetic analysis**

All 107 characters are binary coded and parsimony informative. Each character has a weight of 1. For complex patterns the number of details assigns the weight.

**ES3 Table 3:** List of characters used in the phylogenetic analysis

| **number** | **character** | **character states** | | **weight (number of details)** | **CI**  **acctran/deltran** |
| --- | --- | --- | --- | --- | --- |
|  |  | **apomorphy** | **plesiomorphy** |  |  |
| habitus | | | | | |
| 1 | body | Whole body serrated. | [Not the whole body serrated.] | 7 | 0.5/0.5 |
| 2 |  | Only margins of pereonite 5-7 and pleotelson strongly serrate. | [Not only margins of pereonite 5-7 and pleotelson strongly serrate.] |  | 1.0/1.0 |
| 3 |  | Pereonites and pleotelson laterally expanding into flat marginal flanges. | [Pereonites and pleotelson not with flat lateral extensions.] |  | 1.0/1.0 |
| 4 |  | Body broad. | [Body slender.] |  | 0.5/0.5 |
| 5 |  | Body anteriorly wide and posteriorly slender. | [Body not anteriorly wide and posteriorly slender.] |  | 0.143/0.125 |
| 6 |  | Body elongated. | [Body slender, but not elongated.] |  | 0.167/0.2 |
| 7 |  | Body cigar-like with straight body margins. | [Body not cigar-like with straight body margins.] |  | 1.0/1.0 |
| 8 | pereonites 1-3 | Close packing of pereonites 1-3. | [Pereonites 1-3 not closely packed] | 1 | 1.0/1.0 |
| 9 | pereonite 1 | Pereonite 1 not broad and half of size of pereonite 2 or smaller. | [Pereonite 1 not broad nor half of size of pereonite 2 or smaller.] | 6 (including 47-50 with a weight of 1 for each) | 0.25/0.2 |
| 10 |  | Pereonite 1 broad and clearly smaller than pereonite 2. | [Pereonite 1 not broad and smaller than pereonite 2.] |  | 0.5/0.5 |
| 11 | pereonite 2 | Pereonite 2 largest of pereonites 1-4. | [Pereonites 1-4 subequal.] | 1 | 0.2/0.25 |
| 12 | pereonites 1-4 | Pereonites 1-4 higher than pereonites 5-7. | [Pereonites 1-4 of same height as pereonites 5-7.] | 1 | 0.083/0.1 |
| 13 | pereonites 5-7 | Pereonites 5-7 enlarged. | [Pereonites 5-7 not enlarged.] | 1 | 0.333/0.333 |
| 14 | anterior pereonites | Pereonites 1-4 shorter than pereonites 5-7 | [Pereonites 1-3 shorter than pereonites 4-7.] | 1 | 1.0/1.0 |
| 15 | pereonite 5 | Pereonite 5 clearly convex inflated lateral margins. | [Lateral margins of pereonite 5 not inflated.] | 3 | 0.167/0.167 |
| 16 |  | Pereonite 5 inflated. | [Pereonite 5 not inflated.] |  | 0.5/0.5 |
| 17 |  | Pereonite 5 elongated. | [Pereonite 5 similar in size to pereonite 6.] |  | 0.333/0.333 |
| 18 | pleotelson | Pleotelson enlarged. | [Pleotelson not enlarged.] | 1 | 0.5/0.5 |
| 19 | transverse section | Body highly vaulted in transverse section especially in pleotelson. | [Body in transverse section axis not highly vaulted, lateral fields presenting a continuous profile.] | 1 | 0.333/0.333 |
| 20 | pereonites 4-7 | Pereonites 4-7 posteriorly acute. | [Pereonites 4-7 not posteriorly acute.] | 2 | 1.0/1.0 |
| 21 |  | Posterior corners of the tergits of pereonites 4-7 tipped with one stout spine. | [Posterior corners of the tergits of pereonites 4-7 without a stout spine.] |  | 1.0/1.0 |
| Cephalothorax | | | | | |
| 22 | cephalic keels | Cephalic keels between antennular folds present. | [Cephalic keels between antennular folds absent.] | 1 | 0.5/0.5 |
| 23 | rostrum | Cephalon with rostrum. | [Cephalon without rostrum.] | 1 | 1.0/1.0 |
| 24 | cephalic spine row | Dorsal margin of antennular fold with row of spines resembling a rostral structure. | [Dorsal margin of antennular fold without any spines resembling a rostral structure.] | 1 | 1.0/1.0 |
| 25 | cephalic spine | Margin of antennular fold with one anteriorly directed spine. | [Margin of antennular fold without distinct spine.] | 1 | 0.2/0.2 |
| 26 | antennula | Antennula consisting of 5 articles. | [Antennula consisting out of 6 or more articles.] | 1 | 0.077/0.077 |
| 27 | flagellum of antennula | Antennula with *specialized* distal articles. | [Antennula with *unspecialized* distal articles*.*] | 6 | 0.077/1.0 |
| 28 |  | Flagellum with rounded bulbous last article. | [Flagellum not with rounded bulbous last article.] |  | 0.5/0.5 |
| 29 |  | Antennula with bulbous and long terminal article (clearly longer than wide). | [Terminal article of antennula not bulbous and long.] |  | 1.0/1.0 |
| 30 |  | Flagellar article 1 of antennula smallest. | [Flagellar article 1 of antennula not smallest.] |  | 1.0/1.0 |
| 31 |  | Flagellar article 2 of antennula with elongation holding terminal bulbous article. | [Flagellar article 2 of antennula without elongation holding terminal bulbous article.] |  | 1.0/1.0 |
| 32 |  | Terminal article of antennula bulbous and formed like a ball. | [Terminal article of antennula not bulbous and formed like a ball.] |  | 1.0/1.0 |
| 33 | article 2 of antennula | Article 2 of antennula elongated (twice as long as first peduncular article). | [Article 1 and 2 of antennula of the same size.] | 3 | 0.5/0.5 |
| 34 |  | Article 2 of antennula distally with 3-4 joint articulated broom setae. | [Broom setae sporadically present.] |  | 1.0/1.0 |
| 35 |  | Article 2 of antennula distally with (just) two joint articulated broom setae. | [Article 2 of antennula with more than two joint articulated broom setae at distal end.] |  | 0.071/0.071 |
| 36 | antenna | Shortened and robust antenna, reaching only one quarter of the body length. | [Antenna long and slender, clearly longer than one quarter of the body length.] | 1 | 1.0/1.0 |
| 37 | lacinia mobilis | Lacinia mobilis reduced to one small bulge-like tooth. | [Lacinia mobilis with 3 to 5 teeth.] | 1 | 0.333/0.333 |
| 38 | incisior process | Incisior process bent forward as one strong tooth. | [Incisior process not bent forward as one strong tooth.] | 4 | 1.0/1.0 |
| 39 |  | Incisior process simplified. | [Incisior process with teeth.] |  | 0.5/0.5 |
| 40 |  | Incisor process with strong shelf-like tooth. | [Incisior process not shelf-like.] |  | 0.5/0.5 |
| 41 |  | Incisor process enlarged. | [Incisior process not enlarged.] |  | 1.0/1.0 |
| 42 | mandibular palp | Mandibular palp absent. | [Mandibular palp present.] | 2 | 0.091/0.091 |
| 43 |  | Mandibular palp consisting out of two articles. | [Mandibular palp not consisting out of two articles.] |  | 0.333/0.333 |
| 44 | maxilliped | Retinaculae elongated: more than 3 times longer than width of stalk. | [Retinaculae not elongated: about 2 times longer than width of stalk.] | 1 | 0.5/0.5 |
| 45 | mouthparts | Mouthparts extremely bent forward. | [Mouthparts not bent forward.] | 1 | 0.5/0.5 |
| pereonites | | | | | |
| 46 | sensory seta | Stout sensory setae present anteriorly on tergits 1-4. | [No sensory setae present anteriorly on tergits 1-4.] | 1 | 0.167/0.167 |
| 47 | pereonite 1 | Pereonite 1 broader than pereonite 2. | [Pereonite 1 not broader than pereonite 2.] | 6 (including 9 and 10 with a weight of 1) | 0.167/0.167 |
| 48 |  | Pereonite 1 longer than pereonite 2 (midsagital length). | [Pereonite 1 not longer than pereonite 2.] |  | 0.125/0.143 |
| 49 |  | Pereonite 1 enlarged and clearly bigger (more than 2 times of midsagital length of pereonite 2). | [Pereonite 1 not enlarged and clearly bigger than pereonite 2.] |  | 0.5/0.5 |
| 50 |  | Pereonite 1 shorter and not as broad as pereonite 2. | [Pereonite not shorter than pereonite 2.] |  | 0.083/0.083 |
| 51 | ventral elongations at pereonites 1-5 | Spine-like ventral elongations at pereonites 1 to 5 decreasing in length towards the posterior pereonites. | [Pereonites 1 to 5 without spine-like ventral elongations.] | 1 | 1.0/1.0 |
| 52 | anteriorly directed spine at pereonite 1 | Anteriorly directed spine at pereonite 1. | [Pereonite 1 smooth ventrally.] | 1 | 0.5/0.5 |
| 53 | ventral elongation at pereonites 6 and 7 | Spine-like ventral elongation at the fused pereonites 6 and 7, the elongation at pereonite 6 directed anteriorly, the one at pereonite 7 caudally. | [Pereonites 6 and 7 without fusion or spine-like elongation.] | 1 | 1.0/1.0 |
| 54 | opercular spine | Ventral spine midway on the operculum. | [Operculum without ventral spine.] | 4 | 0.5/1.0 |
| 55 |  | Caudally directed strong spine on pereonite 7. | [Pereonite 7 smooth ventrally.] |  | 0.333/0.25 |
| 56 |  | Curved caudally directed spine located midway on the operculum. | [Operculum without spine.] |  | 1.0/1.0 |
| 57 |  | Straight, caudally directed spine positioned anteriorly on the operculum. | [Operculum without spine.] |  | 0.5/0.5 |
| 58 | fusion of posterior pereonites | Pereonites 6 and 7 fused. | [Pereonites free.] | 3 | 0.2/0.2 |
| 59 |  | Pereonites 6 and 7 fused with pleotelson. | [Pereonites and pleotelson free.] |  | 0.5/0.5 |
| 60 |  | Pereonite 7 and pleotelson fused. | [Pereonites and pleotelson free.] |  | 1.0/1.0 |
| 61 | marginal flanges | Pereonites 6, 7 and pleotelson with marginal flanges. | [Pereonites 6, 7 and pleotelson without marginal flanges.] | 1 | 0.167/0.167 |
| pereopods | | | | | |
| 62 | coxae | Coxae 1-4 with anterolateral elongation | [Coxae without anterolateral elongation.] | 3 | 0.333/0.333 |
| 63 |  | Coxae 1-4 anteriorly tipped with stout seta. | [No stout seta present on anterior tip of coxae 1-4] |  | 0.111/0.111 |
| 64 |  | Coxae produced anteriorly. | [Coxae angular anteriorly, without projection.] |  | 0.167/0.167 |
| 65 | pereopods III and IV | Pereopods I, II, VI and VII longer than pereopods III to V. | [Pereopods of similar length.] | 1 | 1.0/1.0 |
| 66 | posterior pereopods | Pereopods V to VII longer and more heavily built than pereopods II to IV. | [Pereopods V to VII and pereopods II to IV of similar length.] | 3 (including 70 with a weight of 1) | 0.333/0.5 |
| 67 |  | Pereopods V-VII: Ischium elongated (over 5.5 times longer than wide). | [Pereopods V-VII not with elongated ischium.] |  | 1.0/1.0 |
| 68 | pereopod III | Pereopod III dorsally bent. | [Pereopod III not dorsally bent.] | 2 | 1.0/1.0 |
| 69 |  | Dactylus of PIII with row of long setae. | [Dactylus of PIII without row of long setae.] |  | 1.0/1.0 |
| 70 | posterior pereopods | Ventral row of natatory setae at pereopods V to VII absent. | [Ventral row of natatory setae present.] | 3 (including 66 and 67 with a weight of 1 for each) | 0.25/0.25 |
| 71 | pereopod VII | Basis of pereopod VII with long setae. | [No long setae on basis of pereopod VII present.] | 1 | 1.0/1.0 |
| 72 | pereopod I | Lower margin of carpus of pereopod I with composed robust setae in a row. | [Lower margin of carpus of pereopod I not with composed robust setae in a row.] | 35 | 0.333/0.333 |
| 73 |  | Pereopod I: ventral row of setae on carpus reduced due to specialization. | [Pereopod I: ventral row of setae on carpus not reduced due to specialization.] |  | 0.167/0.167 |
| 74 |  | Carpus of pereopod I dorsally bearing a row of long simple setae. | [Carpus of pereopod I dorsally without a row of long simple setae.] |  | 0.333/0.333 |
| 75 |  | Pereopod I: dorsal row of setae on carpus reduced due to specialization. | [Pereopod I: dorsal row of setae on carpus not reduced due to specialization.] |  | 0.111/0.125 |
| 76 |  | Enlargement of pereopod I concentrating on carpus. | [Enlargement of pereopod I not concentrating on carpus.] |  | 0.2/0.25 |
| 77 |  | Enlargement of pereopod I concentrating on propodus. | [Enlargement of pereopod I not concentrating on propodus.] |  | 0.25/0.333 |
| 78 |  | Pereopod I small and slender, but propodus enlarged. | [Pereopod I not small and slender with enlarged propodus.] |  | 1.0/1.0 |
| 79 |  | Pereopod I as functional unit enlarged. | [Pereopod I as functional unit not enlarged.] |  | 0.5/0.333 |
| 80 |  | Propodus of pereopod I ventrally with row of small stout unequally bifid setae. | [Propodus of pereopod I ventrally not with row of small stout unequally bifid setae.] |  | 0.25/0.25 |
| 81 |  | Platform-like gap between propodus and distoventral seta on carpus present. | [Platform-like gap between propodus and distoventral seta on carpus absent.] |  | 1.0/1.0 |
| 82 |  | Carpus of pereopod I enlarged and tapering towards propodus. | [Carpus of pereopod I not enlarged, not tapering towards propodus.] |  | 1.0/1.0 |
| 83 |  | Propodus of pereopod I ventrally fringed with fine hairs and setae breaking through a cuticular membrane. | [Propodus of pereopod I ventrally not fringed with fine hairs and setae breaking through a cuticular membrane.] |  | 0.333/0.333 |
| 84 |  | Carpus of pereopod I enlarged and broadest at articulation to propodus. | [Carpus of pereopod I not enlarged and not broadest at articulation of propodus.] |  | 0.5/0.5 |
| 85 |  | Carpus distolaterally with “claw-seta”. | [Carpus distolaterally not with a “claw-seta”.] |  | 0.5/1.0 |
| 86 |  | Carpus distolaterally produced. | [Carpus distolaterally not produced.] |  | 0.2/0.25 |
| 87 |  | Carpus of pereopod I with 1 composed seta midway. | [Carpus of pereopod I not with 1 composed seta midway.] |  | 0.5/1.0 |
| 88 |  | Ventral setae behind claw-seta small and simple or small and slender. | [Not with ventral setae behind claw-seta small and simple or small and slender.] |  | 0.25/0.5 |
| 89 |  | Carpus of pereopod I enlarged and with setae of irregular size. | [Carpus of pereopod I not enlarged, not with setae in irregular size.] |  | 0.5/0.5 |
| 90 |  | Size of ventral setae on carpus irregular and of varying types. | [Size of ventral setae on carpus not irregular, of same type.] |  | 1.0/1.0 |
| 91 |  | Setae behind claw-seta small, of similar size and type. | [Carpus of pereopod I not with claw-setae and setae not behind claw-seta small, of similar size and type.] |  | 0.5/0.5 |
| 92 |  | Carpus distoventrally with claw-seta and penultimate seta. | [Carpus not with claw-seta and penultimate seta.] |  | 1.0/1.0 |
| 93 |  | Pereopod I robust, articles almost quadrangular. | [Pereopod I not robust, articles not quadrangular.] |  | 1.0/1.0 |
| 94 |  | Setae in ventral row on carpus of pereopod I increasing in length towards propodus. | [Setae in ventral row on carpus of pereopod I not increasing in length towards propodus.] |  | 0.143/0.143 |
| 95 |  | Setae on carpus and propodus of pereopod I not composed. | [Composed setae present on carpus and propodus of pereopod I] |  | 0.143/0.143 |
| 96 |  | Distoventral seta on carpus of pereopod I shortest. | [Distoventral seta of carpus of pereopod I not shortest.] |  | 0.5/0.5 |
| 97 |  | Distoventral seta of carpus reaching full length of propodus. | [Distoventral seta of carpus not reaching full length of propodus.] |  | 0.25/0.25 |
| 98 |  | Second seta behind claw-seta of similar size. | [Not with second seta behind claw-seta of similar size.] |  | 1.0/1.0 |
| 99 |  | Pereopod I slender in comparison to pereopod II. | [Pereopod I not slender in comparison to pereopod II.] |  | 0.5/1.0 |
| 100 |  | Pereopod I slender and ventrally only slender setae present on carpus and propodus. | [Pereopod I not slender and not only slender setae present on carpus and propodus.] |  | 1.0/1.0 |
| 101 |  | Pereopod I small in size, subchelate: propodus enlarged and dactylus folding against propodus. | [Pereopod I not small in size, not subchelate.] |  | 1.0/1.0 |
| 102 |  | Propodus of pereopod I elongated in chela. | [Propodus of pereopod I not elongated in chela.] |  | 1.0/1.0 |
| 103 |  | Propodus of slender pereopod I elongated (over 3.5 times longer than wide). | [Propodus of pereopod I not elongated.] |  | 0.333/0.25 |
| 104 |  | Pereopod I slender: propodus between 4.1 and 5.2 times longer than wide, carpus about 4.5 times longer than wide. | [Pereopod I not slender.] |  | 1.0/1.0 |
| 105 |  | Pereopod I slightly attenuated: propodus of pereopod I between 6 and 9 times longer than wide and carpus between 5 and 7.2 times longer than wide). | [Pereopod I not slightly attenuated.] |  | 1.0/0.5 |
| 106 |  | Pereopod I strongly attenuated (propodus 18.8 times longer than wide, carpus 15 times longer than wide), setae absent on propodus and carpus. | [Pereopod I not strongly attenuated, setae present on propodus and carpus.] |  | 1.0/0.5 |
| 107 | pereopod II | Pereopod II robust, articles almost quadrangular. | [Pereopod II not robust, articles, not quadrangular.] | 6 | 0.5/0.5 |
| 108 |  | Propodus of pereopod II heavily built (carpus and propodus broad). | [Propodus of pereopod II not heavily built.] |  | 1.0/1.0 |
| 109 |  | Carpus of pereopod II bearing a ventral row of composed setae. | [Setae on carpus of pereopod II not standing in ventral rows.] |  | 0.5/0.5 |
| 110 |  | Propodus of pereopod II bearing a ventral row of composed setae. | [Setae on carpus and propodus of pereopod II not standing in rows ventrally.] |  | 0.1/0.1 |
| 111 |  | Carpus and propodus of the pereopod II bearing dorsally a row of long setae. | [Setae on carpus and propodus of pereopod II not standing in rows dorsally.] |  | 0.125/0.111 |
| 112 |  | Basis and ischium of pereopods II and III fringed with distally plumose setae. | [Basis and ischium without this setal type.] |  | 1.0/1.0 |
| 113 | pereopod IV | Pereopod IV folious, carpus and propodus paddle-like. | [Pereopod IV not folious, carpus and propodus resembling carpus and propodus of pereopod III.] | 2 | 1.0/1.0 |
| 114 |  | Carpus and propodus surrounded (with dense row of) by numerous distally plumose setae. | [Carpus and propodus not surrounded by numerous distally plumose setae.] |  | 1.0/1.0 |
| 115 | pereopod VII | Ischium dorsally with anteriorly directed cuticular hook. | [Ischium dorsally smooth.] | 4 | 1.0/1.0 |
| 116 |  | Propodus and carpus of pereopod VII with long setae dorsally. | [No long setae dorsally of propodus and carpus of pereopod VII.] |  | 0.1/0.091 |
| 117 |  | Propodus and carpus of pereopod VII with long setae dorsally. | [No long setae dorsally of propodus and carpus of pereopod VII.] |  | 0.167/0.167 |
| 118 |  | Basis of pereopod VII with long slender “swimming setae”. | [Basis of pereopod VII without “swimming setae”.] |  | 1.0/1.0 |
| pleotelson | | | | | |
| 119 | inflation | Pleotelson dorsally inflated. | [Pleotelson dorsally not inflated.] | 1 | 0.25/0.333 |
| 120 | anus region | Anus region separated and bilobed. | [Anus region not separated and bilobed.] | 1 | 1.0/1.0 |
| 121 | form | Pleotelson vaulted in transverse section. | [Pleotelson not vaulted in transverse section.] | 1 | 0.333/0.25 |
| 122 | branchial chamber | Branchial chamber and operculum in relation to size of pleotelson small, operculum of oval shape and posterior part broadest. | [Branchial chamber and operculum covering nearly the whole ventral view of pleotelson.] | 2 | 1.0/1.0 |
| 123 |  | Branchial chamber and operculum in relation to size of pleotelson small, rounded. | [Branchial chamber not small and rounded.] |  | 1.0/1.0 |
| 124 | uropods | Uropods uniramous. | [Uropods biramous.] | 6 | 0.125/0.125 |
| 125 |  | Uropodal sympod extremely elongated; styliform. | [Uropodal sympod not elongated, shorter than endopod.] |  | 1.0/1.0 |
| 126 |  | Uropods cover anus valves. | [Uropods not covering anus valves.] |  | 0.167/0.167 |
| 127 |  | Uropods short, not overlapping posterior margin of pleotelson. | [Uropods overlapping posterior margin of pleotelson.] |  | 0.5/0.5 |
| 128 |  | Uropodal endopodite nearly bulbous. | [uropodal endopodite clearly longer than wide.] |  | 1.0/1.0 |
| 129 |  | Uropodal exopod reduced to half of size of endopod or less. | [Uropodal exopod not reduced to half of size of endopod or less.] |  | 0.143/0.125 |

In total, 294 trees with a length of 405 steps were retained. The trees had a consistency index (CI) of 0.3185, a homoplasy index (HI) of 0.6815 and a retention index (RI) of 0.8182.

**ES3 Figure 7:** 50 percent majority rule tree, numbers refer to clades as listed in ES Table 4

*Macrostylis angolensis
Macrostylis meteorae
Macrostylis robusta
Austroniscus chelus
Austroniscus obscurus
Austroniscus ovalis
Nannoniscoides gigas
Nannoniscoides latediffusus
Nannoniscoides biscutatus
Nannoniscoides coronarius
Eugerdella atypicum
Desmosoma hessiera
Desmosoma ochotense
Mirabilicoxa cornuta
Mirabilicoxa atlanticum
Cryodesma polare
Chelator insignis
Chelator verecundus
Chelator chelatum
Chelator vulgaris
Chelator* sp. nov. A
new species A
*Reductosoma gunnera
Disparella kensleyi
Disparella funalis
Disparella maiuscula
Disparella neomana
Disparella pachythrix
Disparella valida
Prochelator hampsoni
Prochelator lateralis
Prochelator litus
Prochelator uncatus
Prochelator abyssalis
Prochelator maorii
Prochelator angolensis
Prochelator incomitatus
Oecidiobranchus nanseni
Oecidiobranchus plebejum
Paradesmosoma australis
Paradesmosoma conforme
Paradesmosoma Orientale
Cryodesma agnari
Cryodesma cryoabyssale
Eugerdella natator
Eugerdella nonfunalis
Eugerdella theodori
Eugerdella pugilator
Eugerdella serrata
Whoia dumbshafensis
Whoia victoriensis
Thaumastosoma platycarpus
Thaumastosoma tenue
Whoia angusta
Whoia variabilis
Echinopleura cephalomagna
Desmosoma lineare
Desmosoma thoracicum
Desmosoma stroembergi
Desmosoma gigantea
Desmosoma latipes
Pseudogerda arctica
Eugerda reticulata
Pseudogerda intermedia
Eugerda tenuimana
Pseudogerda kamchatica
Pseudogerda anversense
Pseudogerda elegans
Desmosoma tetarta
Desmosoma renatae
Torwolia creper
Torwolia subchelatus
Torwolia tinbinae
Mirabilicoxa gracilipes
Mirabilicoxa acuminata
Mirabilicoxa acuta
Mirabilicoxa similis
Echinopleura aculeata
Mirabilicoxa similipes
Mirabilicoxa alberti
Mirabilicoxa plana
Momedossa longipedis
Momedossa profunda
Pseudogerda hessleri
Pseudogerda ischnomesoides
Pseudomesus pitombo
Pseudomesus satanus
Pseudomesus brevicornis*genus novum *fletcheri
Saetoniscus meteori
Exiliniscus clipeatus
Exiliniscus aculeatus
Panetela wolffi
Panetela tenella
Rapaniscus dewdneyi
Rapaniscus crassipes
Rapaniscus multisetosus
Rapaniscus* sp. nov. A
*Regabellator profugus
Regabellator abyssi
Nannonisconus latipleonus
Nannonisconus carinatus
Nannoniscus bidens
Nannoniscus teres
Hebefustis alleni
Hebefustis vafer
Hebefustis mollicellus*


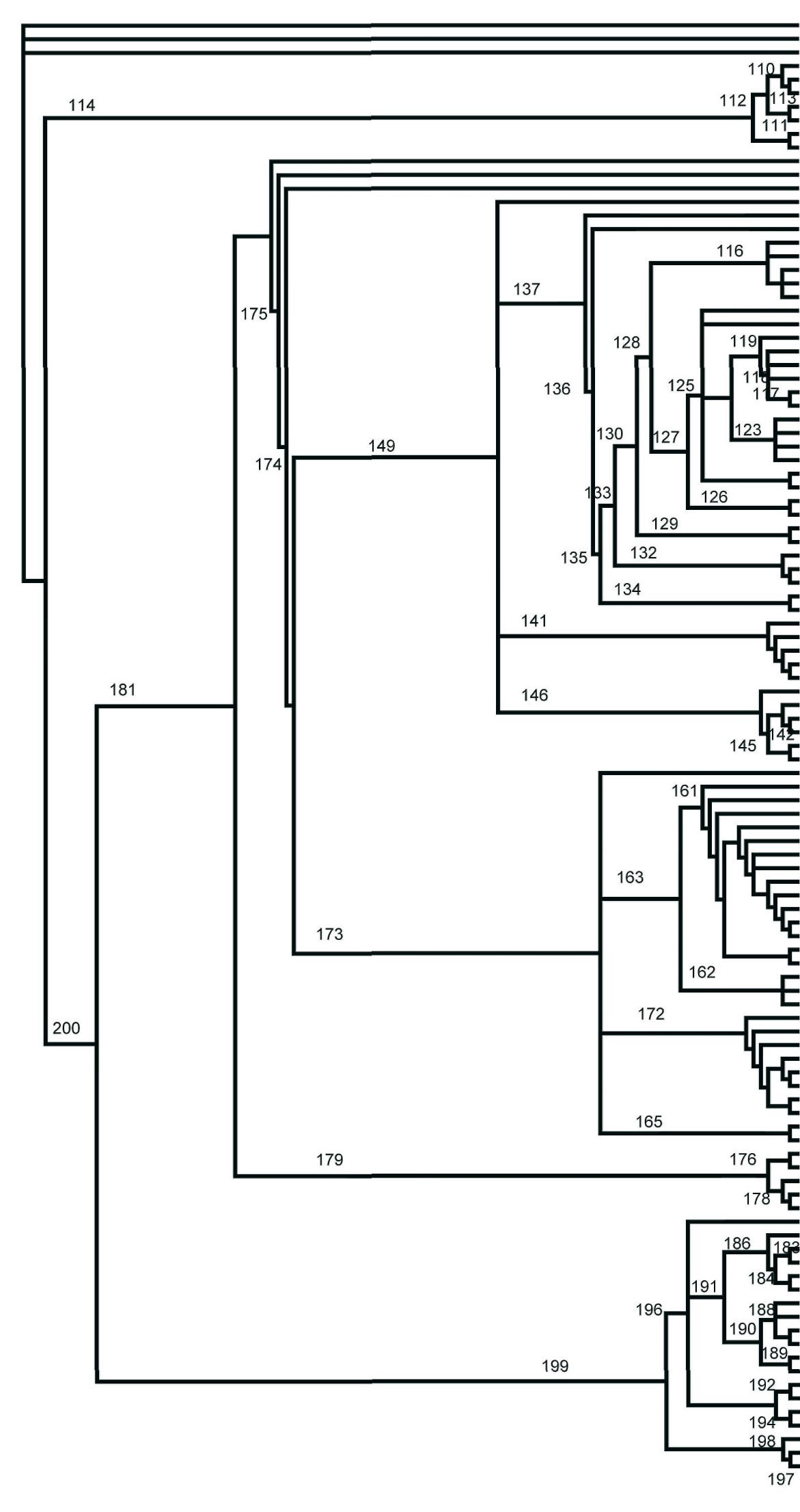


*Macrostylis angolensis
Macrostylis meteorae
Macrostylis robusta
Austroniscus chelus
Austroniscus obscurus
Austroniscus ovalis
Nannoniscoides gigas
Nannoniscoides latediffusus
Nannoniscoides biscutatus
Nannoniscoides coronarius
Desmosoma atypicum
Desmosoma hesslera
Desmosoma ochotense
Mirabilicoxa atlanticum
Echinopleura cephalomagna
Mirabilicoxa cornuta
Cryodesma polare*

*Prochelator abyssalis
Prochelator hampsoni
Prochelator lateralis
Prochelator litus
Prochelator uncatus
Prochelator maorii
Reductosoma gunnera
Chelator insignis
Chelator verecundus
Chelator chelatum
Chelator vulgaris
Chelator* sp.  *Disparella kensleyi
Disparella funalis
Disparella pachythrix
Disparella valida
Disparella maiuscula
Disparella neomana
Oecidiobranchus nanseni
Oecidiobranchus plebejum
Prochelator angolensis
Prochelator incomitatus
Paradesmosoma australis
Paradesmosoma conforme
Paradesmosoma Orientale
Cryodesma agnari
Cryodesma cryoabyssale
Desmosoma lineare
Desmosoma thoracicum
Desmosoma stroembergi
Desmosoma arctica
Eugerda intermedia
Desmosoma latipes
Desmosoma gigantea
Eugerda reticulata
Eugerda tenuimana
Eugerda kamchatica
Desmosoma anversense
Desmosoma elegans
Desmosoma tetarta
Desmosoma renatae
Torwolia creper
Torwolia subchelatus
Torwolia tinbinae
Mirabilicoxa gracilipes
Mirabilicoxa acuminata
Mirabilicoxa acuta
Mirabilicoxa alberti
Mirabilicoxa plana
Mirabilicoxa similis
Echinopleura aculeata
Mirabilicoxa similipes
Eugerdella natator
Eugerdella
Eugerdella theodori
Eugerdella pugilator
Eugerdella serrata
Momedossa longipedis
Momedossa profunda
Whoia dumbshafensis
Whoia victoriensis
Thaumastosoma platycarpus
Thaumastosoma tenue
Whoia angusta
Whoia variabilis
Eugerdella hessleri
Eugerdella ischnomesoides
Pseudomesus satanus
Pseudomesus pitombo
Pseudomesus brevicornis
Nymphodora* *fletcheri*
*Saetoniscus meteori
Exiliniscus clipeatus
Exiliniscus aculeatus
Panetela wolffi
Panetela tenella
Nannonisconus latipleonus
Nannonisconus carinatus
Nannoniscus bidens
Nannoniscus teres
Rapaniscus dewdneyi
Rapaniscus crassipes
Rapaniscus multisetosus
Rapaniscus centauri
Regabellator profugus
Regabellator abyssi
Hebefustis alleni
Hebefustis vafer
Hebefustis mollicellus*


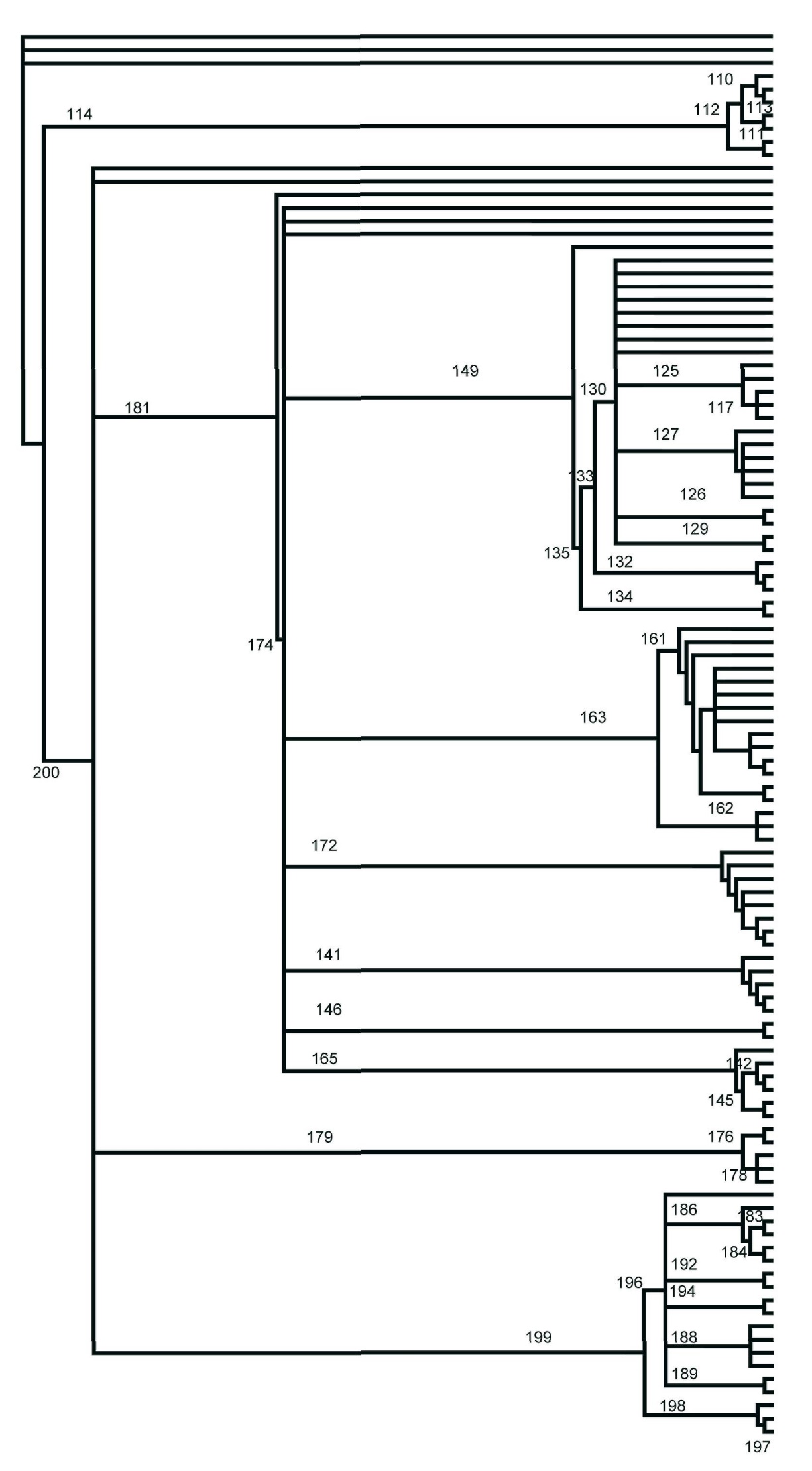
**ES3 Figure 8:** 80 percent majority rule tree, numbers refer to clades as listed in ES Table 4

*Macrostylis angolensis
Macrostylis meteorae
Macrostylis robusta
Desmosoma atypicum
Desmosoma hesslera
Austroniscus chelus
Austroniscus obscurus
Austroniscus ovalis
Nannoniscoides gigas
Nannoniscoides latediffusus
Nannoniscoides biscutatus
Nannoniscoides coronarius
Desmosoma ochotense
Mirabilicoxa atlanticum
Echinopleura cephalomagna
Mirabilicoxa cornuta
Cryodesma polare*

*Prochelator abyssalis
Prochelator hampsoni
Prochelator lateralis
Prochelator litus
Prochelator uncatus
Prochelator maorii
Reductosoma gunnera
Chelator insignis
Chelator verecundus
Chelator chelatum
Chelator vulgaris
Chelator* sp. *Disparella kensleyi
Disparella funalis
Disparella pachythrix
Disparella valida
Disparella maiuscula
Disparella neomana
Oecidiobranchus nanseni
Oecidiobranchus plebejum
Prochelator angolensis
Prochelator incomitatus
Paradesmosoma australis
Paradesmosoma conforme
Paradesmosoma Orientale
Cryodesma agnari
Cryodesma cryoabyssale
Desmosoma lineare
Desmosoma thoracicum
Desmosoma stroembergi
Desmosoma arctica
Eugerda tenuimana
Eugerda intermedia
Desmosoma latipes
Desmosoma gigantea
Desmosoma kamchatica
Euqerda reticulata
Desmosoma anversense
Eugerda elegans
Desmosoma tetarta
Desmosoma renatae
Torwolia creper
Torwolia subchelatus
Torwolia tinbinae
Mirabilicoxa gracilipes
Mirabilicoxa acuminata
Mirabilicoxa acuta
Mirabilicoxa alberti
Mirabilicoxa plana
Mirabilicoxa similis
Echinopleura aculeata
Mirabilicoxa similipes
Eugerdella natator
Eugerdella
Eugerdella theodori
Eugerdella pugilator
Eugerdella serrata
Momedossa longipedis
Momedossa profunda
Whoia dumbshafensis
Whoia victoriensis
Thaumastosoma platycarpus
Thaumastosoma tenue
Whoia angusta
Whoia variabilis
Eudergella hessleri
Eudergella ischnomesoides
Pseudomesus satanus
Pseudomesus pitombo
Pseudomesus brevicornis
Nymphodora fletcheri
Saetoniscus meteori
Exiliniscus clipeatus
Exiliniscus aculeatus
Panetela wolffi
Panetela tenella
Hebefustis alleni
Hebefustis vafer
Hebefustis mollicellus
Nannonisconus latipleonus
Nannonisconus carinatus
Nannoniscus bidens
Nannoniscus teres
Rapaniscus dewdneyi
Rapaniscus crassipes
Rapaniscus multisetosus
Rapaniscus centauri
Regabellator profugus
Regabellator abyssi*

**Character distribution in the trees**

**ES3 Table 4:** Characters defining the branches

(+ indicates the presence of the node in the tree; - indicates a polytomy; for the comparison of the apomorphy list, the trees with the greatest distance were used, here: two trees with a distance of 68, numbers of nodes refer to tree 284)

| **clade** | **taxa** | **character(s)** | **strict consensus** | **majrule 80** | **majrule 50** |
| --- | --- | --- | --- | --- | --- |
|  |  | apomorphy list acctran |  |  |  |
|  |  | trees 284/242 |  |  |  |
| 202 | Macrostylidae | 26 (1>2), 8 (2>1), 14 (1>2), 20 (2>1), 21 (2>1), 33 (1>2), 34 (1>2), 42 (2>1), 50 (1>2), 65 (2>1), 68 (2>1), 69 (2>1), 71 (2>1), 118 (2>1), 125 (2>1) | **+** | **+** | **+** |
| 201🡪 114 |  | 4 (1>2), 10 (1>2), 22 (1>2), 95 (1>2) | **-** | **+** | **+** |
| 114🡪112 | *Austroniscus, Nannoniscoides* | 3 (1>2) | **+** | **+** | **+** |
| 112🡪110 | *Austroniscus* | 122 (1>2), 126 (1>2) | **+** | **+** | **+** |
| 110🡪 | *A. chelus* | 35 (1>2) | **+** | **+** | **+** |
| 110🡪109 | *A. obscurus, A. ovalis* | 95 (2>1) | **+** | **+** | **+** |
| 112🡪111 | *N. gigas, N. latipleonus* | 28 (1>2), 29 (1>2), 58 (1>2) | **+** | **+** | **+** |
| 114🡪113 | *Nannoniscella* | 25 (1>2) | **+** | **+** | **+** |
| 113🡪 | *N. biscutatus, N. coronarius* | 35 (1>2) | **+** | **+** | **+** |
| 201🡪200 | Desmosomatidae | 26 (1>2), 72 (1>2), 109 (1>2), 110 (1>2) | **-** | **+** | **+** |
| 🡪181 | Desmosomatidae | 9 (1>2), 46 (2>1), 70 (2>1), 74 (1>2), 94 (1>2), 117 (1>2), 124 (1>2), 63 (1>2), 64 (1>2) | **-** | **-** | **+** |
| 200🡪199 |  | 28 (1>2), 58 (1>2), 126 (1>2) | **+** | **+** | **+** |
| 199🡪196 | without *Hebefustis* | 27 (1>2), 30 (1>2), 31 (1>2), 32 (1>2) | **-** | **+** | **+** |
| 199🡪198 | *Hebefustis* | 12 (1>2), 25 (1>2) | **+** | **+** | **+** |
| 198🡪197 | *H. vafer, H. mollicellua* | 80 (1>2) | **+** | **+** | **+** |
| 198🡪 | *H. alleni* | 50 (2>1) | **+** | **+** | **+** |
| 196🡪191 | *Saetoniscus meteori, Exiliniscus, Panetela, Rapaniscus, Regabellator* | 129 (1>2) | **-** | **-** | **+** |
| 191🡪186 | *Saetoniscus meteori, Exiliniscus, Panetela* | 6 (1>2), 7 (1>2), 110 (2>1), 116 (1>2) | **+** | **+** | **+** |
| 186🡪185 | *Exiliniscus, Panetela* | 46 (2>1), 58 (2>1) | **+** | **+** | **+** |
| 186🡪 | *Saetoniscus meteori* | 57 (1>2), 70 (2>1), 111 (1>2), 117 (1>2), 119 (1>2), 127 (1>2) | **+** | **+** | **+** |
| 185🡪183 | *Exiliniscus* | 23 (1>2), 33 (2>1), 36 (1>2), 42 (1>2) | **+** | **+** | **+** |
| 183🡪 | *E. clipeatus* | 74 (1>2), 111 (1>2) | **+** | **+** | **+** |
| 185🡪184 | *Panetela* | 35 (1>2), 50 (2>1), 72 (1>2), 95 (1>2), 109 (2>1) | **+** | **+** | **+** |
| 184🡪 | *P. wolffi* | 15 (1>2), 70 (2>1) | **+** | **+** | **+** |
| 184🡪 | *P. tenella* | 116 (2>1) | **+** | **+** | **+** |
| 191🡪190 | *Rapaniscus, Regabellator* | 12 (1>2) | **-** | **-** | **+** |
| 190🡪188 | *Rapaniscus* | 50 (2>1), 55 (1>2), 76 (1>2), 79 (1>2), 80 (1>2), 82 (1>2) | **+** | **+** | **+** |
| 188🡪 | *R. crassipes* | 63 (1>2) | **-** | **-** | **+** |
| 188🡪187 | *R. multisetosus, R. centauri* | 54 (1>2) | **-** | **-** | **+** |
| 187🡪 | *R. centauri* | 55 (2>1) | **-** | **-** | **+** |
| 190🡪189 | *Regabellator* | 42 (1>2), 53 (1>2), 64 (1>2), 70 (2>1), 72 (2>1), 95 (1>2), 111 (1>2), 117 (1>2) | **+** | **+** | **+** |
| 189🡪 | *R. profugus* | 63 (1>2) | **+** | **+** | **+** |
| 196🡪192 | *Nannonisconus* | 18 (1>2), 22 (1>2), 58 (2>1), 60 (1>2), 120 (1>2) | **+** | **+** | **+** |
| 192🡪 | *N. latipleonus* | 48 (1>2), 50 (2>1) | **+** | **+** | **+** |
| 192🡪 | *N. canalicatus* | 47 (1>2) | **+** | **+** | **+** |
| 196🡪194 | *Nannoniscus* | 4 (1>2), 10 (1>2), 22 (1>2), 111 (1>2) | **+** | **+** | **+** |
| 194🡪 | *N. bidens* | 57 (1>2), 74 (1>2) | **+** | **+** | **+** |
| 194🡪 | *N. teres* | 46 (1>2), 55 (1>2) | **+** | **+** | **+** |
| 196🡪 | *Nymphodora fletcheri* | 59 (1>2), 124 (1>2) | **+** | **+** | **+** |
| 181🡪175 | Eugerdellatinae and Desmosomatinae | 12 (1>2), 75 (1>2), 111 (1>2), 116 (1>2) | **-** | **-** | **+** |
| 🡪179 |  | 6 (1>2), 35 (1>2), 74 (2>1), 119 (1>2), 9 (2>1), 17 (1>2), 18 (1>2), 70 (1>2), 94 (2>1), 110 (2>1) | **+** | **+** | **+** |
| 179🡪176 | *Desmosoma/ Eugerdella* | 67 (1>2), 96 (1>2), 117 (2>1) | **-** | **+** | **+** |
| 176🡪 | *E. hessleri* | 15 (1>2), 16 (1>2), 50 (2>1), 110 (1>2), 119 (2>1) | **-** | **+** | **+** |
| 176🡪 | *E. ischnomesoides* | 12 (1>2), 26 (2>1), 111 (1>2) | **-** | **+** | **+** |
| 179🡪178 | *Pseudomesus* | 115 (1>2), 126 (1>2), 127 (1>2), 128 (1>2) | **+** | **+** | **+** |
| 178🡪177 | *P. satanus, P. brevicornis* | 63 (2>1) | **-** | **-** | **+** |
| 177🡪 | *P. brevicornis* | 42 (1>2) | **-** | **-** | **+** |
| 175🡪 | *Desmosoma ochotense* | 43 (1>2), 73 (1>2), 95 (1>2) | **+** | **+** | **+** |
| 175🡪174 | Desmosomatinae and Eugerdellatinae without *D. ochotense* | 5 (1>2), 9 (2>1), 26 (2>1) | **+** | **+** | **+** |
| 174🡪149 | Eugerdellatinae (including *Mirabilicoxa cornuta* and *M. atlanticum*) | 50 (2>1) | **-** | **-** | **+** |
| 174🡪173 | Desmosomatinae (except for *Mirabilicoxa cornuta*) | 99 (1>2), 103 (1>2) | **-** | **-** | **+** |
| 🡪163 | *Torwolia, Desmosoma, Eugerda* | 5 (2>1), 13 (1>2), 66 (1>2), 108 (1>2) | **+** | **+** | **+** |
| 163🡪161 | *Desmosoma, Eugerda* | 12 (2>1), 61 (1>2), 100 (1>2) | **+** | **+** | **+** |
| 163🡪162 | *Torwolia* | 11 (1>2), 15 (1>2), 16 (1>2), 78 (1>2), 94 (2>1), 101 (1>2), 103 (2>1”), 116 (2>1) | **+** | **+** | **+** |
| 🡪 | *Echinopleura cephalomagna* | 1 (1>2), 37 (1>2), 39 (1>2), 45 (1>2), 73 (2>1) | **+** | **+** | **+** |
| 🡪165 | *Momedossa* | 12 (2>1), 17 (1>2), 40 (1>2), 124 (2>1), 129 (1>2) | **+** | **+** | **+** |
| 165🡪 | *M. profunda* | 75 (2>1), 111 (2>1) | **+** | **+** | **+** |
| 161🡪 | *Desmosoma lineare* | 9 (2>1) | **+** | **+** | **+** |
| 161🡪160 | *D. thoracicum, D. stroembergi, Eugerda* | 95 (1>2) | **+** | **+** | **+** |
| 160🡪159 | *D. stroembergi, Eugerda* | 73 (1>2), 94 (2>1) | **+** | **+** | **+** |
| 159🡪 | *D. stroembergi* | 26 (1>2) | **+** | **+** | **+** |
| 159🡪158 | *Desmosoma, Eugerda* | 11 (1>2), 124 (2>1), 129 (1>2) | **+** | **+** | **+** |
| 158🡪156 | *Eugerda* | 104 (1>2) | **+** | **+** | **+** |
| 156🡪154 | *Eugerda* | 12 (1>2) | **+** | **+** | **+** |
| 154🡪 | *Desmosoma latipes* | 43 (1>2) | **+** | **+** | **+** |
| 154🡪153 |  | 105 (1>2) | **+** | **+** | **+** |
| 153🡪152 |  | 13 (1>2), 26 (2>1), 66 (2>1) | **+** | **+** | **+** |
| 152🡪150 |  | 61 (2>1) | **+** | **+** | **+** |
| 150🡪 | *Desmosoma elegans* | 6 (1>2), 42 (2>1),16 (2>1) | **+** | **+** | **+** |
| 152🡪151 |  | 11 (2>1) | **+** | **+** | **+** |
| 151🡪 | *Eugerda tenuimana* | 26 (1>2), 106 (2>1), 116 (2>1), 117 (2>1) | **+** | **+** | **+** |
| 151🡪 | *Desmosoma kamchatica* | 35 (1>2) | **+** | **+** | **+** |
| 156🡪155 | *Eugerda reticulata, Desmosoma gigantea* | 105 (1>2) | **+** | **+** | **+** |
| 155🡪 | *Eugerda reticulata* | 106 (2>1) | **+** | **+** | **+** |
| 158🡪157 | *Desmosoma tetarta, Desmosoma renatae* | 75 (2>1), 94 (1>2), 95 (2>1), 126 (1>2) | **+** | **+** | **+** |
| 157🡪 | *Desmosoma tetarta* | 12 (1>2), 73 (2>1) | **+** | **+** | **+** |
| 173🡪172 | *Mirabilicoxa* (including *Echinopleura aculeata*) | 42 (1>2),61 (1>2) | **+** | **+** | **+** |
| 172🡪171 | *Mirabilicoxa* (including *Echinopleura aculeata*) | 62 (1>2), 110 (2>1) | **+** | **+** | **+** |
| 171🡪170 | *Mirabilicoxa* (including *Echinopleura aculeata*) | 26 (1>2), 116 (2>1) | **+** | **+** | **+** |
| 170🡪168 | *Mirabilicoxa* (including *Echinopleura aculeata*) | 61 (2>1) | **+** | **+** | **+** |
| 168🡪167 | *Mirabilicoxa* (including *Echinopleura aculeata*) | 62 (2>1) | **+** | **+** | **+** |
| 167🡪 | *Echinopleura aculeata* | 1 (1>2), 35 (1>2), 37 (1>2), 39 (1>2) | **+** | **+** | **+** |
| 167🡪 | *Mirabilicoxa similipes* | 42 (2>1) | **+** | **+** | **+** |
| 170🡪169 |  | 50 (2>1) | **+** | **+** | **+** |
| 169🡪 | *Mirabilicoxa alberti* | 42 (2>1), 48 (1>2), 116 (1>2) | **+** | **+** | **+** |
| 169🡪 | *Mirabilicoxa plana* | 35 (1>2) | **+** | **+** | **+** |
| 171🡪 | *Mirabilicoxa acuminata* | 225 (1>2), 35 (1>2) | **+** | **+** | **+** |
| 🡪137 | clade of chelate genera | 35 (1>2) | **+** | **+** | **+** |
| 🡪141 | *Eugerdella* | 76 (1>2), 79 (1>2), 89 (1>2), 94 (2>1) | **+** | **+** | **+** |
| 🡪146 | *Whoia, Thaumastosoma* | 15 (1>2), 107 (1>2), 121 (1>2), 126 (1>2) | **+** | **+** | **+** |
| 🡪 | *Mirabilicoxa cornuta* | 25 (1>2), 61v,62 (1>2), 76 (1>2) | **-** | **-** | **+** |
| 137🡪 | *Mirabilicoxa atlanticum* | 103 (1>2), 124 (2>1), 129 (1>2) | **-** | **-** | **+** |
| 137🡪136 | clade of chelate genera | 77 (1>2), 79 (1>2), 83 (1>2) | **+** | **+** | **+** |
| 141🡪 | *Eugerdella natator* | 47 (1>2), 75 (1>2), 83 (1>2), 124 (2>1), 129 (1>2) | **+** | **+** | **+** |
| 141🡪140 | *E. theodori, E. serrata, E. pugilator* | 81 (1>2), 96 (1>2) | **+** | **+** | **+** |
| 140🡪139 | *E. theodori,E . serrata, E. pugilator* | 5 (1>2), 26 (1>2), 35 (1>2), 77 (1>2), 80 (1>2) | **+** | **+** | **+** |
| 139🡪138 | *E. serrata, E. pugilator* | 2 (1>2), 24 (1>2), 49 (1>2), 51 (1>2) | **+** | **+** | **+** |
| 138🡪 | *E. pugilator* | 47 (1>2), 75 (1>2), 116 (2>1) | **+** | **+** | **+** |
| 138🡪 | *E. serrata* | 110 (2>1), 111 (2>1) | **+** | **+** | **+** |
| 146🡪145 | *Whoia, Thaumastosoma* without *W. dumbshafensis* | 93 (1>2), 97 (1>2), 110 (2>1) | **+** | **+** | **+** |
| 145🡪143 | *Thaumastosma* and *W. victoriensis* | 15 (2>1), 124 (2>1), 129 (1>2) | **+** | **+** | **+** |
| 143🡪142 | *Thaumastosoma* | 6 (1>2), 38 (1>2), 44 (1>2), 45 (1>2), 46 (1>2), 47 (1>2), 55 (1>2), 56 (1>2), 61 (1>2), 107 (2>1), 117 (2>1), 121 (2>1) | **+** | **+** | **+** |
| 143🡪 | *Whoia victoriensis* | 37 (1>2), 50 (1>2) | **+** | **+** | **+** |
| 145🡪144 | *W. angusta, W. variabilis* | 17 (1>2), 64 (2>1) | **+** | **+** | **+** |
| 146🡪 | *Whoia dumbshafensis* | 48 (2>1), 63 (2>1) | **+** | **+** | **+** |
| 136🡪135 | clade of chelate genera without *Cryodesma polare* | 75 (1>2), 85 (1>2), 97 (1>2) | **-** | **+** | **+** |
| 135🡪 134 | *C. agnari, C. cryoabyssale* | 98 (1>2) | **+** | **+** | **+** |
| 134🡪 | *C. cryoabyssale* | 47 (1>2), 83(2>1) | **+** | **+** | **+** |
| 135🡪133 | clade of chelate genera without *Cryodesma* | 5 (2>1), 26 (1>2), 48 (2>1), 84 (1>2) | **+** | **+** | **+** |
| 133🡪132 | *Paradesmosoma* | 43 (1>2), 63 (2>1), 89 (1>2), 90 (1>2), 97 (2>1), 112 (1>2), 113 (1>2), 114 (1>2) | **+** | **+** | **+** |
| 133🡪130 | clade of chelate genera without *Cryodesma* and *Paradesmosoma* | 73 (1>2), 92 (1>2), 94 (2>1) | **+** | **+** | **+** |
| 130🡪129 | *Oecidiobranchus* | 19 (1>2), 42 (1>2), 50 (1>2), 121 (1>2), 123 (1>2) | **+** | **+** | **+** |
| 130🡪128 | *Prochelator, Reductosoma,* new species A*, Chelator, Disparella* | 48 (1>2), 86 (1>2) | **-** | **-** | **+** |
| 128🡪127 | *Prochelator, Disparella, Reductosoma,* new species A | 87 (1>2), 110 (2>1) | **-** | **-** | **+** |
| 127🡪126 | *P. angolensis, P. incomitatus* | 5 (1>2), 49 (1>2) | **-** | **-** | **+** |
| 127🡪125 | *Prochelator, Disparella, Reductosoma,* new species A without *P. angolensis* and *P. incomitatus* | 124 (2>1), 129 (1>2) | **-** | **-** | **+** |
| 🡪116 | *Chelator* | 121 (1>2) | **+** | **+** | **+** |
| 116🡪115 | *C. vulgaris, C. chelatum* and *C. antarcticus* | 91 (1>2) | **+** | **+** | **+** |
| 🡪119 | *Disparella* | 5 (1>2), 25 (1>2), 40 (1>2), 97 (2>1), 102 (1>2), 124 (2>1), 129 (1>2) | **+** | **+** | **+** |
| 119🡪118 | *Disparella* without *D. kensleyi* | 41 (1>2), 86 (2>1), 91 (1>2) | **-** | **-** | **+** |
| 118🡪117 | *D. funalis, D. valida* | 88 (2>1) | **-** | **-** | **+** |
| 🡪123 | *Prochelator* without *P. angolensis* and *P. incomitatus* | 116 (2>1) | **-** | **-** | **+** |
| 🡪 | *Reductosoma* | 6 (2>1), 12 (2>1), 19 (1>2), 35 (2>1), 42 (1>2), 44 (1>2), 63 (2>1), 64 (2>1), 84 (2>1) | **+** | **+** | **+** |

In the following, the major clades of the ingroup are described. Accelerated (Acctran) and delayed transformation (Deltran) produced relatively similar results. The synapomorphy patterns produced by Acctran were a little bit more convincing for those characters known as synapomorphies for the genera (subfamilies), therefore these results are shown. In the following, numbers in square brackets refer to the characters (Table 4).

Clade 114 consists of *Austroniscus* and *Nannoniscoides* and is defined by following synapomorphies: body broad [4], pereonite 1 broad and clearly smaller than pereonite 2 [10], cephalon with cephalic keels [22] and ventral setae on carpus of pereopod I of "non-composed" seta-type [95]. *Austroniscus* (clade 110) is characterized by following synapomorphies: branchial chamber and operculum in relation to pleotelson small and posteriorly broadest [122], uropods not covering anus valves [126]. Clade 112 includes *Austroniscus* (clade 110) and species of *Nannoniscoides* (clade 113) and is the sister group to clade 111 (species of *Nannoniscoides*). Clade 113 is based on the apomorphy: margin of antennular fold with one anteriorly directed spine [25]. Clade 111 is characterized by following apomorphies: flagellum of antennula with bulbous distal article [28], form of distal article elongated [29], pereonites 6 and 7 fused [58].

Clade 200 (50 and 80 percent majority rule tree only) includes all Desmosomatidae except *Austroniscus* and *Nannoniscoides* based on following synapomorphies: antennula consisting of 5 articles [26], ventral margin of carpus of pereopod I with composed robust setae standing in a row [72], carpus of pereopod II ventrally with row of composed setae [109], propodus of pereopod II ventrally with a row of composed setae [110].

Clade 199 consists of all genera with a specialized antennula (*Hebefustis*, *Regabellator*, *Rapaniscus*, *Nannoniscus*, *Nannonisconus*, *Panetela*, *Saetoniscus* and genus novum *fletcheri*) and is defined by following synapomorphies: flagellum of antennula with bulbous distal article [28], pereonites 6 and 7 fused [58], uropods covering anus valves [126]. In the 50 and 80 percent majority rule tree, *Hebefustis* (clade 198) is resolved as sister taxon to clade 196 based on following synapomorphies: pereonites 1-4 higher than pereonites 5-7 [12], margin of antennular fold with one anteriorly directed spine [25]. Clade 198 is defined by following synapomorphies: antennula with specialized distal articles [27], first article of flagellum of antennula smallest [30], second article of flagellum of antennula with elongation holding third bulbous article [31], distal article of flagellum bulbous and ball-shaped [32].

Clade 186 is defined by body elongated (more than five times longer than width of pereonite 2) [6], with straight, cigar-like body margins [6], propodus of pereopod II without setal row [110], carpus and propodus of pereopod V dorsally with rows of long setae [116]. It includes the genera *Saetoniscus*, *Exiliniscus* and *Panetela*. *Panetela* (clade 184) and *Exiliniscus* (clade 183) are sister taxa.

*Nannonisconus* (clade 192) is based on following synapomorphies: pleotelson enlarged [18], cephalon with cephalic keels [22], pereonites 6 and 7 not fused [58], pereonites 7 and pleotelson fused [60], anus region separated and bilobed [120]. *Nannoniscus* (clade 194) is defined by: body broad [4], pereonite 1 broad and clearly smaller than pereonite 2 [10], cephalon with cephalic keels [22], carpus and propodus of pereopod II bearing dorsal rows of setae [111].

*Rapaniscus* (clade 188) is defined by following synapomorphies: pereonite 1 not shorter than pereonite 2 [50], pereonite 7 with strong, caudally directed spine [55], pereopod I concentrating in enlargement of the carpus [76], pereopod I as functional unit enlarged [79], propodus of pereopod I with ventral row of small stout setae [80], carpus of pereopod I enlarged and tapering towards the propodus [82].

*Regabellator* (clade 189) is characterized by: mandibular palp absent [42], pereonites 6 and 7 with anteriorly directed spine [53], coxae produced [64], natatory setae present on pereopods V-VII [70], lower margin of carpus of pereopod I without composed setae [72], ventral setae on carpus of pereopod I of not-composed seta type [95], carpus and propodus of pereopod II bearing dorsally rows of setae [111].

Clade 179 includes *Pseudomesus* and is defined by following synapomorphies: body elongated (more than five times longer than width of pereonite 2) [6], second article of antennula with only two articulated broom setae [35], carpus of pereopod I dorsally without a row of simple setae [74], pleotelson dorsally inflated [119], pereonite 1 not broad and half of size of pereonite 2 [9], pereonite 5 elongated [17], pleotelson enlarged [18], pereopods V-VII rows of natatory setae absent [70], setae on carpus of pereopod I not standing in a row and increasing in length towards the propodus [94], propodus of pereopod II ventrally without row of composed setae [110]. In the 50 percent majority rule tree clade 179 is the sister taxon to Desmosomatinae including *Desmosoma atypicum*, while *D. atypicum* is resolved to be the sister taxon to all taxa included in Desmosomatinae and Eugerdellatinae (clade 175). In the 80 percent majority rule tree and the strict consensus *D. atypicum* branches polytom after clade 201. Clade 176 is resolved in both majority rule trees and defined by following synapomorphies: ischium of pereopods V-VII elongated (over 3.5 times longer than wide) [67], distoventral seta in the ventral row on the carpus of pereopod I shortest [96], carpus and propodus of pereopod V without natatory setae [117]. *Pseudomesus* (clade 178) is based on following synapomorphies: ischium of pereopods V-VII with dorsal hook [115], uropods covering anus valves [126], uropods short, not overlapping posterior margin of pleotelson [127], uropodal endite bulbous [128].

The following synapomorphies define clade 181 (Desmosomatinae including Eugerdellatinae): pereonite 1 broad and half of size of pereonite 2 [9], tergits 1-4 anteriorly without sensory spines [46], at pereopods V-VII rows of natatory setae present [70], carpus of pereopod I dorsally bearing a row of simple setae [74], ventral setae in row on carpus of pereopod I increasing in length towards propodus [94], carpus and propodus of pereopod V-VII ventrally with rows of long setae [117], uropods uniramous [124], coxae 1-4 anteriorly tipped with seta [63], coxae produced [64].

*Momedossa* (clade 165) is defined by following synapomorphies: pereonites 1-4 of same height as pereonite 5-7 [12], pereonite 5 elongated [17], incisior process shelf-like [40], uropods biramous [124], uropodal exopod reduced to half of size of endopod or less [129]. Clade 172 includes the *Mirabilicoxa* species, except *M. atlanticum* and *M. cornuta*, and *Echinopleura aculeata.* This clade is defined by: mandibular palp absent [42], pereonites 6, 7 and pleotelson with marginal flanges [61].

The group *Torwolia*, *Desmosoma* and *Eugerda* is united in clade 163 based on following synapomorphies: body not anteriorly wide and posteriorly slender [5], pereonites 5-7 enlarged [13], pereopods V-VII longer and more heavily built than pereopods I-IV [66], pereopod II heavily build, carpus and propodus broad [108]. *Torwolia* (clade 162) is characterized by: pereonite 2 largest of pereonites 1-4 [11], pereonite 5 with convex lateral margins [15], pereonite 5 inflated [16], pereopod I small and slender, but propodus enlarged [78], ventral setae on carpus of pereopod I composed [94], pereopod I much shorter than pereopod II [101], propodus of slender pereopod I not elongated [103], carpus and propodus of pereopod V without dorsal rows of natatory setae [116]. Clade 161 includes *Desmosoma* and *Eugerda* and is based on following synapomorphies: pereonites 1-4 of same height as pereonites 5-7 [12], pereonites 6, 7 and pleotelson with marginal flanges [61], pereopod I slender and only slender setae on carpus and propodus [100].

In the 50 percent majority rule tree clade 149 consists of all Eugerdellatinae and is defined by following synapomorphy: pereonite 1 not shorter than pereonite 2 [50]. In the 80 percent majority rule tree and the strict consensus, the genera *Eugerdella* (clade 141, defined by: pereopod I not concentrating in enlargement of carpus [76], pereopod I as functional unit enlarged [79], carpus of pereopod I enlarged and with setae of irregular size [89], ventral setae on carpus of pereopod I not standing in a row and increasing in size [94]) and *Whoia* (clade 146, defined by: lateral margins of pereonite 5 convex [15], pereopod II robust (articles almost quadrangular) [107], pleotelson vaulted in transverse section [121], uropods covering anus valves [126]) are not included in 149, but are polytom at the same node as clade 149.

Clade 134 is formed by *Cryodesma agnari* and *C. cryoabyssale* and inserts at a trichtome node next to *C. polare* and clade 133. The sister species (clade 134) are united by following synapomorphy: penultimate seta in ventral row of the carpus of pereopod I as long as distoventral seta [98].

Clade 133 comprises all genera with a carpo-euchela except *Cryodesma* and is defined by following synapomorphies: body not anteriorly wide and posteriorly slender [5], antennula consisting of five articles [26], pereonite 1 not longer than pereonite 2 (in midsagital length) [48], carpus of pereopod I enlarged and broadest at articulation towards propodus [84].

*Paradesmosoma* (clade 132) is the sister taxon to clade 130 and based on following synapomorphies: mandibular palp of two articles [43], coxae 1-4 without seta on anterior tip [63], carpus of perepod I enlarged and with setae of irregular size and type [89], ventral setae on the carpus of pereopod I composed [90], distal ventral setae not reaching full length of propodus [97], basis of pereopods II and III fringed with numerous distally plumose setae [112], pereopod IV folious, carpus and propodus paddle-like [113], carpus and propodus of pereopod IV surrounded by numerous distally plumose setae [114].

The relationships of the genera *Oecidiobranchus*, *Disparella*, *Chelator*, *Prochelator* as well as *Reductosoma* are not resolved (polytomy) in the 80 percent majority rule and the strict consensus tree.

Clade 129 includes the *Oecidiobranchus* species and is characterized by following synapomorphies: body in transverse section highly vaulted, especially in pleotelson [19], madibular palp absent [42], pereonite 1 shorter than pereonite 2 [50], pleotelson vaulted in transverse section [121], branchial chamber and operculum in relation to size of pleotelson small and rounded [123]. *Disparella* (clade 127) is defined by carpus of pereopod I with single composed seta midway [87], propodus of pereopod II ventrally with row of composed setae [110]. Clade 116 consists of species of the genus *Chelator* and is based on pleotelson vaulted in transverse section [121].

Two *Prochelator* species *P. angolensis and P. incomitatus* form a clade (126) based on body anteriorly wide and posteriorly slender [5], pereonite 1 enlarged and clearly bigger (in midsagital length more than two times longer than pereonite 2) [49]. The remaining *Prochelator* species do not from a clade and branch polytom. In the 50 percent majority rule tree these *Prochelator* species form clade 123 based on the apomorphy: carpus and propodus of pereopod V dorsally without rows of natatory setae [116].
