# Supplementary figures and images for: Evolution and phylogeny of the deep-sea isopod families Desmosomatidae Sars, 1897 and Nannoniscidae Hansen, 1916 (Isopoda: Asellota)
Source: Org Divers Evol. 2021 Oct 13;21(4):691–717. doi: 10.1007/s13127-021-00509-9 (PMC8510888; doi:10.1007/s13127-021-00509-9)

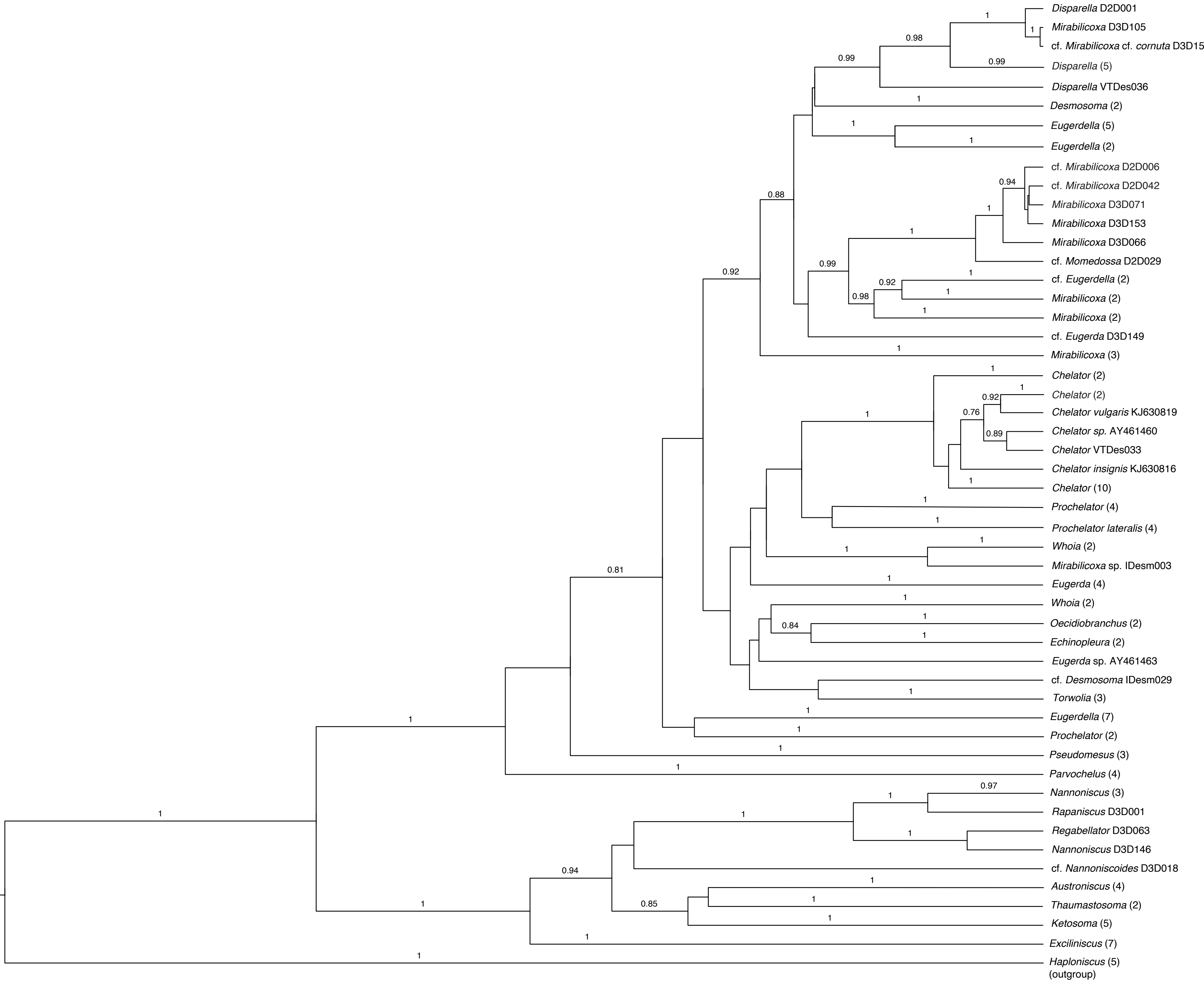

Supplement: Supplementary file 4 — Supplementary file4 (PDF 166 KB). Bayesian phylogenetic tree for 18S. Identical taxa have been collapsed with counts in parentheses. Numbers on branches indicate Bayesian posterior probabilities, shown when greater than 0.75. [file 13127_2021_509_MOESM4_ESM.pdf]

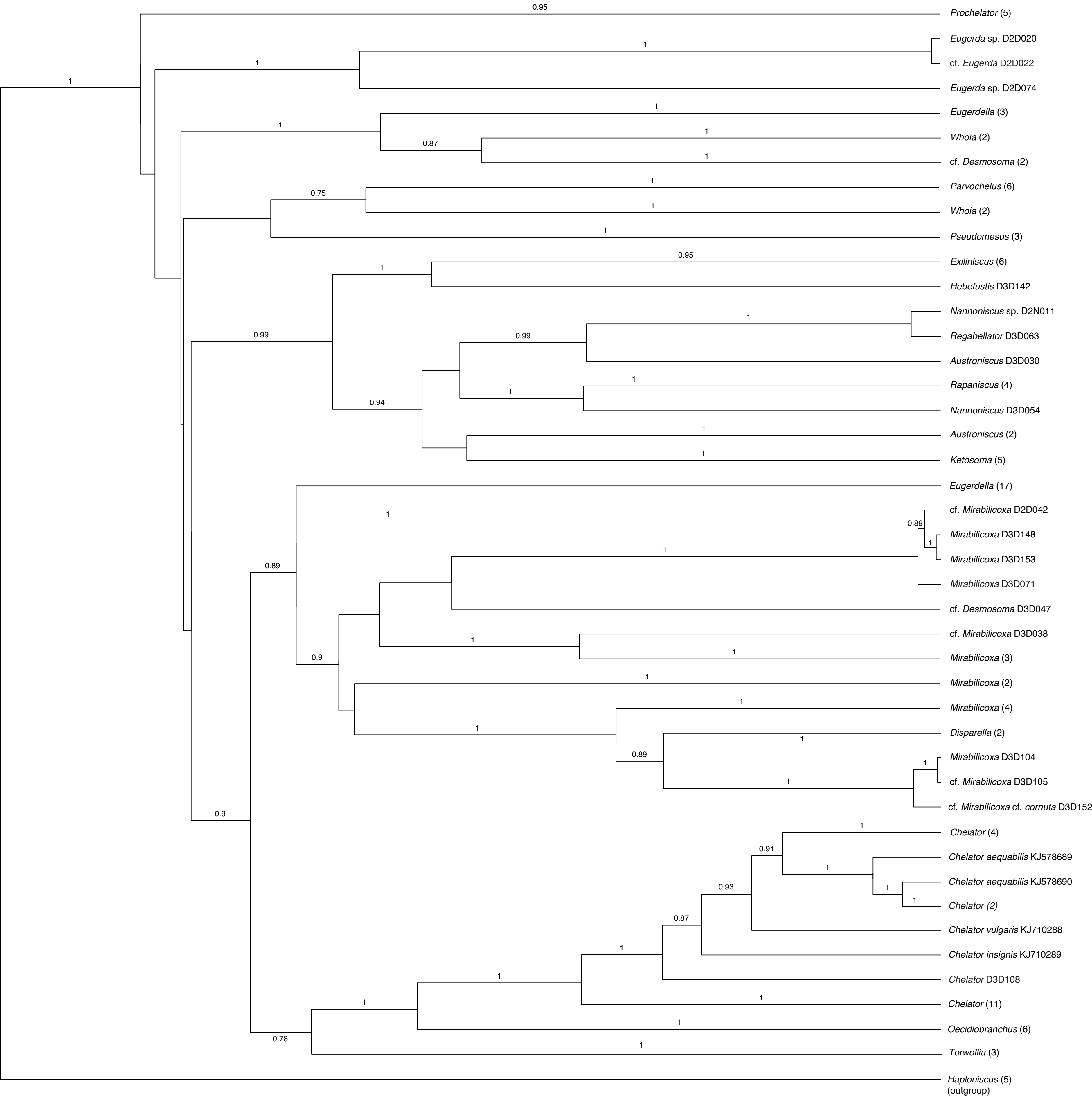

0.1

Supplement: Supplementary file 5 — Supplementary file5 (PDF 162 KB). Bayesian phylogenetic tree for COI. Format and labelling as in Electronic Supplement 4. [file 13127_2021_509_MOESM5_ESM.pdf]

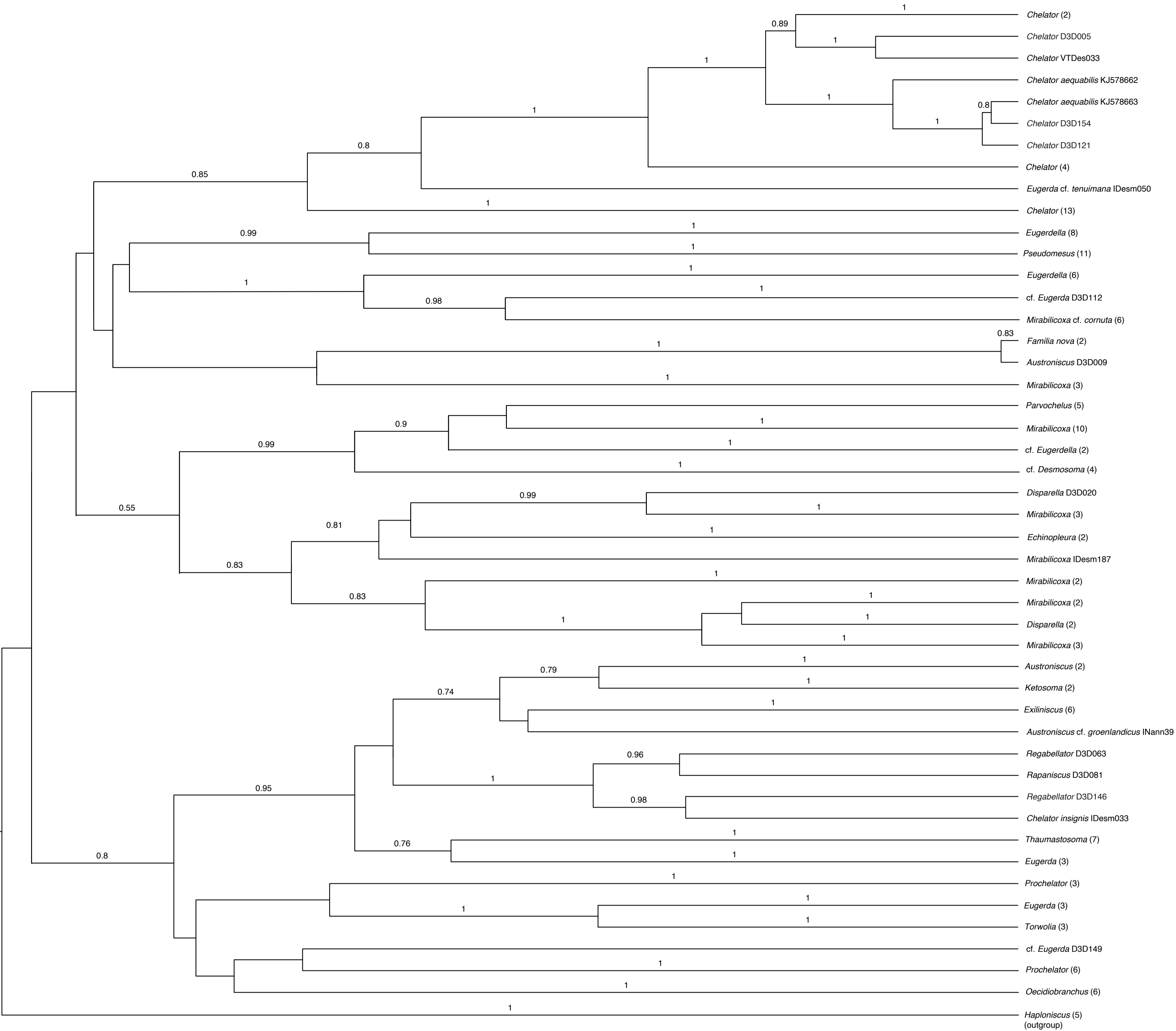

Supplement: Supplementary file 6 — Supplementary file6 (PDF 168 KB). Bayesian phylogenetic tree for 16S. Format and labelling as in Electronic Supplement 4. [file 13127_2021_509_MOESM6_ESM.pdf]

- ARG
- BRA
- CAP
- GUI
- ICE
- IRM
- REY
- SO
- VEM

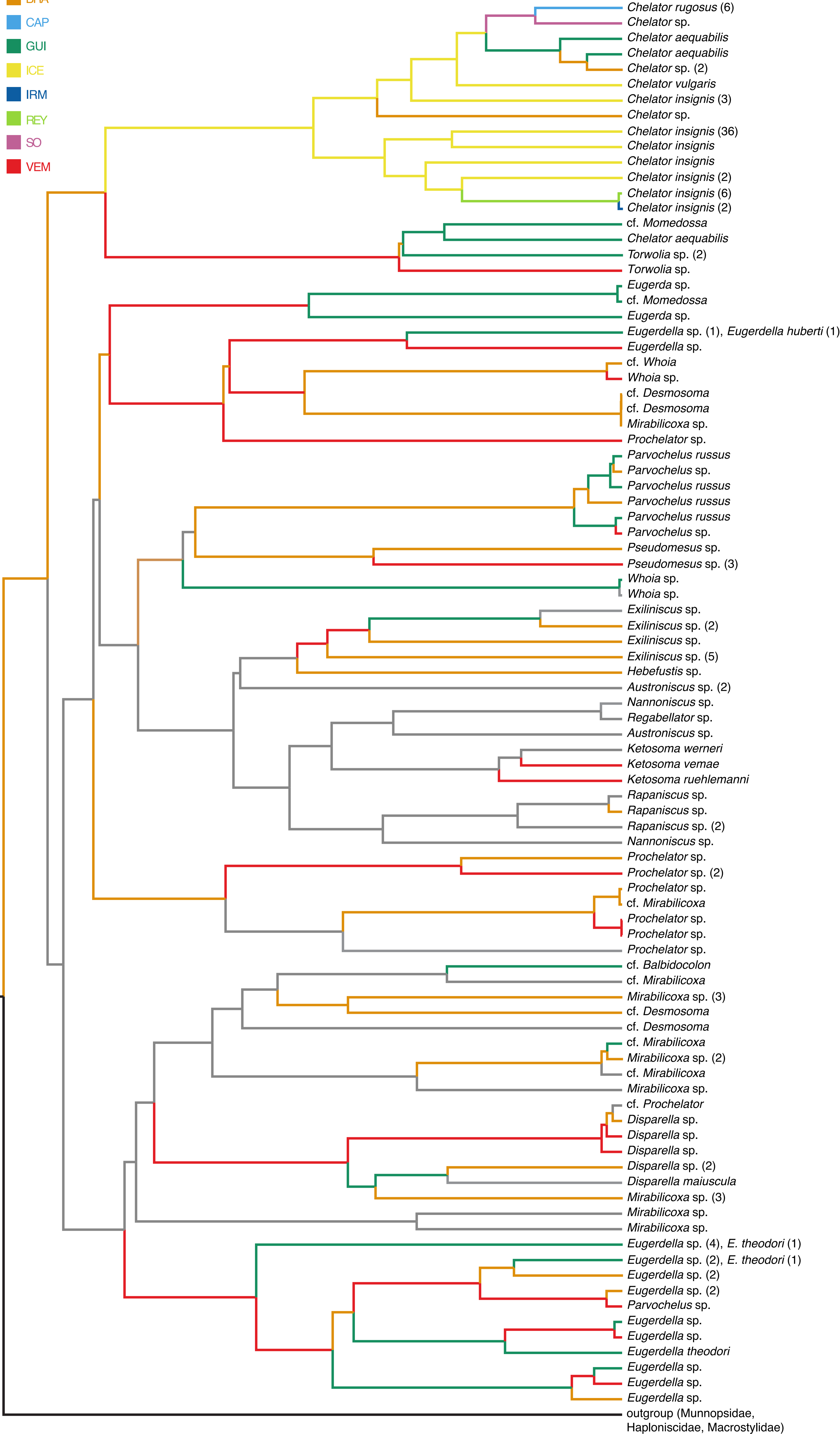

0.09

Supplement: Supplementary file 7 — Supplementary file7 (PDF 175 KB). Phylogenetic tree for COI Bayesian geographical analysis. Tip branch colours indicate oceanic basin where specimens were sampled, and interior indicate colours estimated ancestral location along those branches; see legend at upper left. Taxa have been collapsed where both taxonomy and sampling locality were identical, with counts in parentheses. [file 13127_2021_509_MOESM7_ESM.pdf]

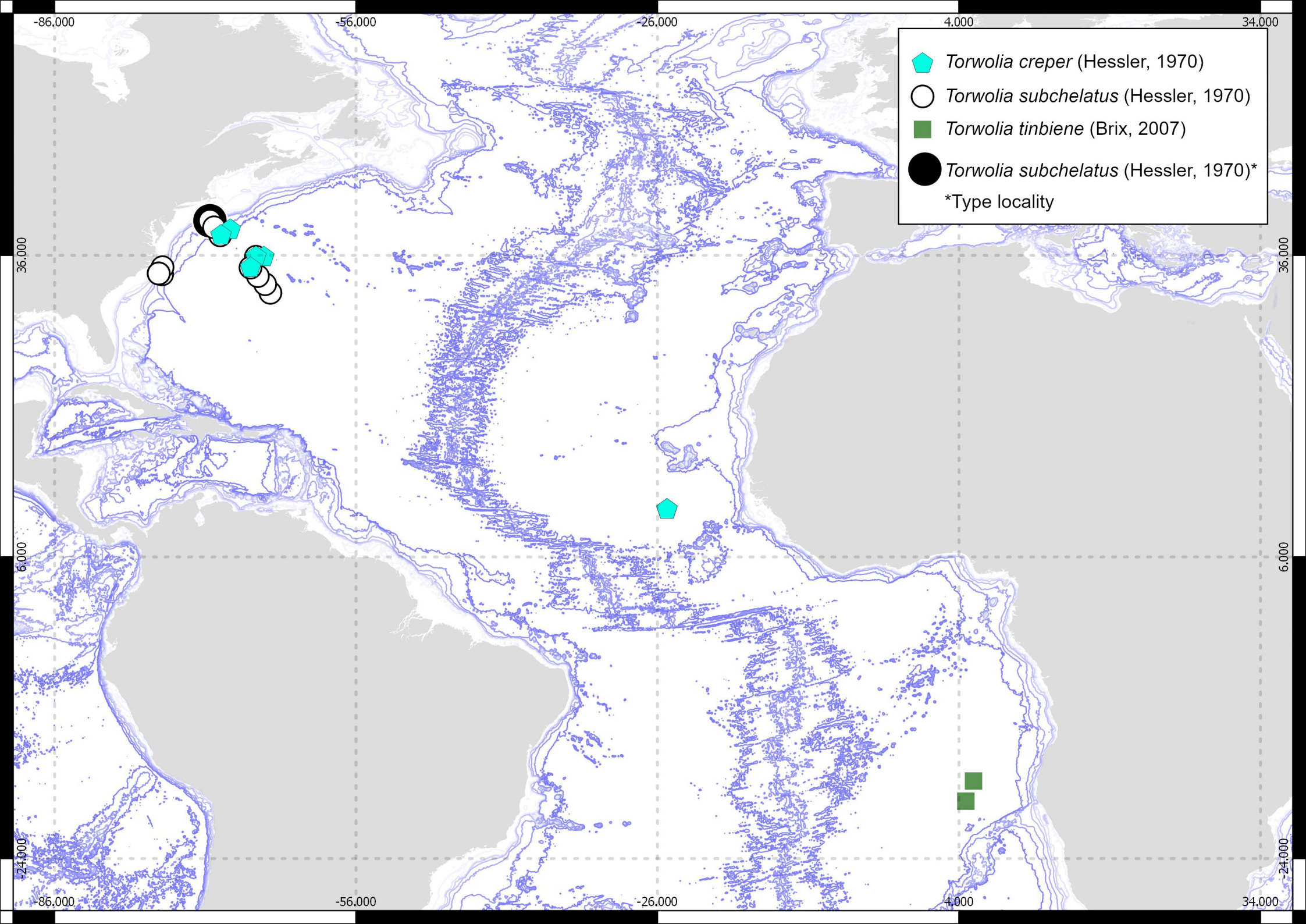

Supplement: Supplementary file 8 — Supplementary file8 (PDF 2 MB). Distribution map for the genus Torwolia Hessler, 1970 including all species described worldwide. [file 13127_2021_509_MOESM8_ESM.pdf]

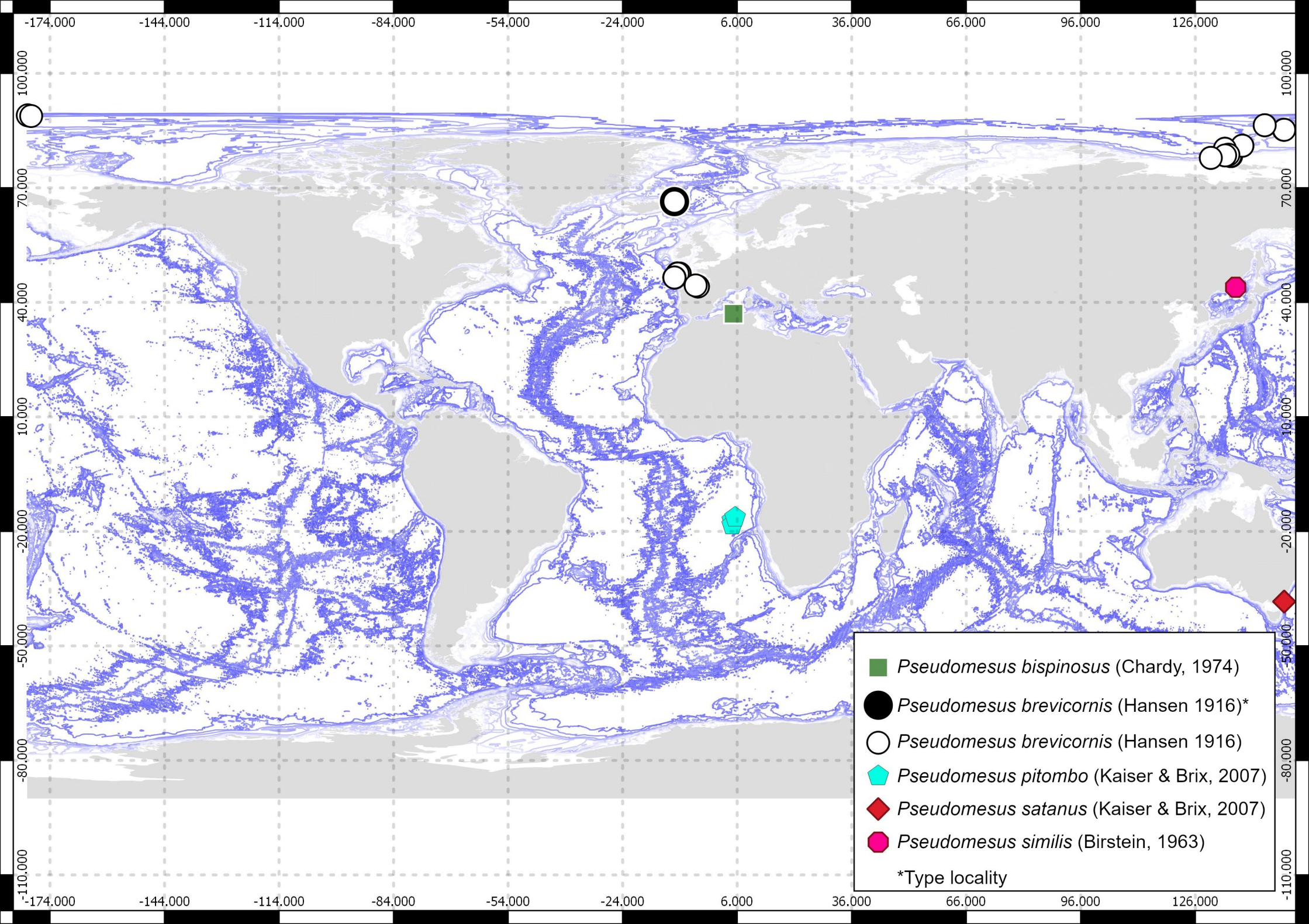

Supplement: Supplementary file 9 — Supplementary file9 (PDF 2 MB). Distribution map for the genus Pseudomesus Hansen, 1916 including all species described worldwide. [file 13127_2021_509_MOESM9_ESM.pdf]

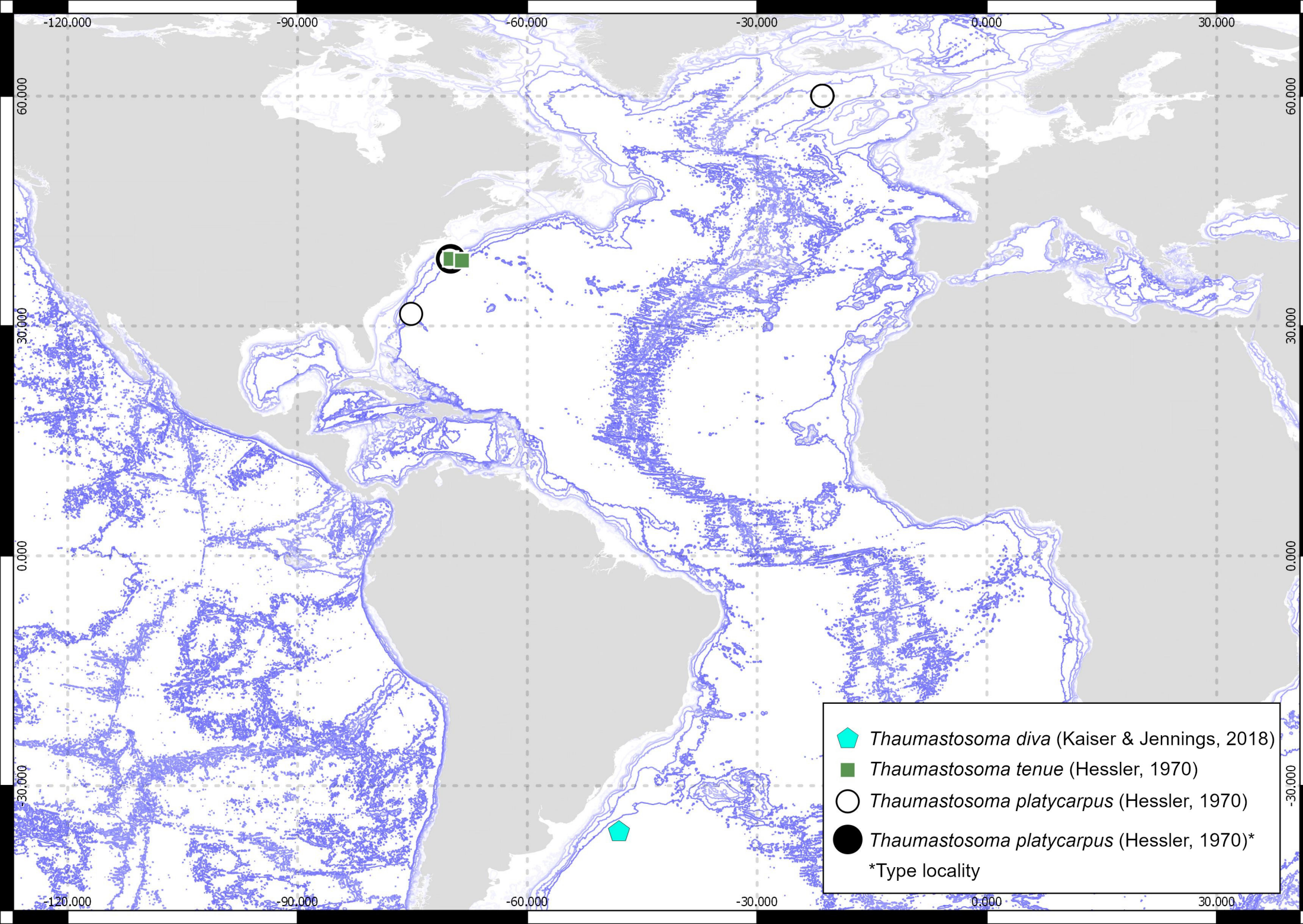

Supplement: Supplementary file 10 — Supplementary file10 (PDF 2 MB). Distribution map for the genus Thaumastosoma Hessler, 1970 including all species described worldwide. [file 13127_2021_509_MOESM10_ESM.pdf]
